# A multiscale model of the regulation of aquaporin 2 recycling

**DOI:** 10.1038/s41540-022-00223-y

**Published:** 2022-05-09

**Authors:** Christoph Leberecht, Michael Schroeder, Dirk Labudde

**Affiliations:** 1 Biotechnology Center (BIOTEC), TU Dresden, Dresden, 01307 Germany; 2 University of Applied Sciences Mittweida, Mittweida, 09648 Germany

**Keywords:** Computer modelling, Signal processing

## Abstract

The response of cells to their environment is driven by a variety of proteins and messenger molecules. In eukaryotes, their distribution and location in the cell are regulated by the vesicular transport system. The transport of aquaporin 2 between membrane and storage region is a crucial part of the water reabsorption in renal principal cells, and its malfunction can lead to Diabetes insipidus. To understand the regulation of this system, we aggregated pathways and mechanisms from literature and derived three models in a hypothesis-driven approach. Furthermore, we combined the models to a single system to gain insight into key regulatory mechanisms of Aquaporin 2 recycling. To achieve this, we developed a multiscale computational framework for the modeling and simulation of cellular systems. The analysis of the system rationalizes that the compartmentalization of cAMP in renal principal cells is a result of the protein kinase A signalosome and can only occur if specific cellular components are observed in conjunction. Endocytotic and exocytotic processes are inherently connected and can be regulated by the same protein kinase A signal.

## Introduction

### Protein kinase A and its regulation by cAMP

cAMP is a ubiquitous second messenger that mediates the intracellular response to extracellular stimuli. After a signal has been recognized by G protein-coupled receptors, adenylyl cyclases (AC) catalyze the conversion of ATP to cAMP. Protein Kinase A (PKA) is activated by cAMP. PKA was one of the earliest kinases to be discovered and was extensively studied ever since^1–3^. Still today, the mechanism of PKA activation by cAMP and its intricate specificity is under ongoing investigation^4–7^. A part of the solution is the assembly of so-called signalosomes in intact cells^8^. A-kinase anchoring proteins (AKAPs) are scaffolds that organize the local and temporal interaction of proteins involved in a signaling cascade^9^. AKAPs are a key component of the cell type-specific response and occur in many isoforms and tissues^10–12^. The dynamic organization of proteins that cooperate in AKAP-based signaling pathways is a basic requisite for the specific processing of signals^13^. It is well known that PKA in its inactive form is a heterotetramer consisting of two regulatory (PKAR) and two catalytic (PKAC) subunits. The regulatory subunits inhibit PKAC in their basal state and dissociate from the catalytic subunits once two molecules of cAMP bind^14^. It was uncovered that the autophosphorylation at serine 144, which reduced the affinity for the regulatory subunit, happens in the absence of cAMP and can lead to a positive feedback loop^15^. The phosphorylated PKAR subunits are rescued by phosphatases (PP) which can further modify the response and signal dynamics^16,17^. Probably the most idiosyncratic properties of PKA activator cAMP is its varying diffusion coefficient^18^ when comparing in vitro and in vivo experiments^19^. Phosphodiesterases (PDE) are a key component when considering the movement and dispersal of cAMP. PDEs are efficiently able to hydrolyze cAMP to AMP and have been shown to restrict its distribution to certain regions of the cell^20^. Nevertheless, when examining experimental and in silico studies^6,21,22^ cAMP diffusion coefficients vary from 5 μm^2^ s^−1^ to 780 μm^2^ s^−1^, and still, some models are using PDE concentrations beyond physiological ranges to restrict cAMP movement accordingly^21^. Latest research has unveiled that a combination of phase separation and cAMP buffering is the root of the phenomenon^6,7^. The different apparent diffusion coefficients are the result of frequent binding and unbinding events of cAMP, such that the fraction of cAMP that is able to move through the cell is low. Additionally, PDEs are able to create nanometer-sized domains of reduced cAMP concentrations that are able to fine-tune the amount of cAMP that is able to bind to relevant PKAR subunits. Taken together, a localized cAMP signal is able to affect a specific subpopulation of cAMP effector proteins depending on the concentration, localization, and composition of the signalosome.

### Aquaporin 2 recycling

**Fig. 1:**
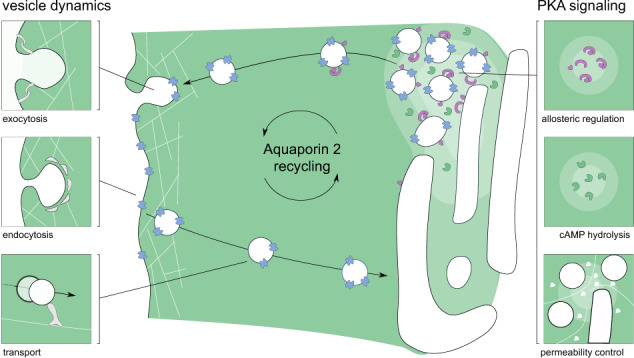
Overview of key mechanisms involved in Aquaporin 2 recycling. Macroscopic phenomena are displayed on the left side of the figure, whereas microscopic phenomena are displayed on the right. AQP2-positive vesicles are transported from the intracellular storage region to the apical membrane upon cAMP signal.

In eukaryotic cells, the distribution of molecular components is managed by the vesicular transport system. Molecular motors transport proteins and small molecule cargo from storage and synthesis sites to areas where they are required for their respective cellular functions^23^. The cytoskeletal network of microtubule and actin filaments is a flexible and dynamic scaffolding system that not only allows for the directed transport but also for regulation and even interference with signaling cascades^24–26^. The redistribution of the water-channel protein aquaporin 2 (AQP2)^27^ from intracellular storage vesicles to the membrane is a prominent example of the complexity of the cellular signaling and transport system. The signaling cascade that triggers the redistribution of AQP2 is initialized by the antidiuretic hormone, arginine vasopressin, that binds to the G protein-coupled receptor V2R. This activation causes the production of cAMP by AC and the subsequent cascade that leads to active PKA, which is able to phosphorylate AQP2 at serine 256^28^. Other phosphorylation sites of AQP2 are known (serine 261, 264, and 269) and there are multiple cellular mechanisms involved to archive AQP2 accumulation at its target site: the apical cell membrane^29–31^. The importance and interplay of the phosphorylation sites involved in this pathway are slowly being unraveled and reviewed elsewhere^32,33^. We would like to highlight the key components of this work. Klussmann et al. discovered that AKAPs are required to translocate AQP2 to the apical membrane^34^ and laid the foundation for the subsequent interest of AKAP as focal points for signal protein localization^8^. Known proteins that act as scaffolds in the AQP2 pathway are AKAP18*δ* (AKAP-7)^11,35^, AKAP220 (AKAP-11)^36,37^, as well as AKAP-Lbc (AKAP-13)^34,38^ although their expression and mode of action seem to be dependent on renal cell type and location in the kidney^39^. Recently, STUB1 was also found to be involved in AQP2 mediating dephosphorylation of AQP2 at serine 261, leading to a decrease in poly-ubiquitination^31^. The scaffolding protein plays a major role in the signaling process, and the absence or presence of key molecules can make or break the signaling process. Which proteins and pathways are affected by a signalosome is vastly dependent on their ability to form localized groups that are able to stay together throughout the signaling process, therefore the AKAP should be the starting point to understanding a signaling cascade. The models build in this work focus on AKAP18*δ* and its involvement in the recycling process. AKAP18*δ* provides binding sites for phosphodiesterase type 4D3 (PDE4)^40,41^, Serine/threonine-protein phosphatase 2B (PP2B)^42^, and of course protein kinase A regulatory subunit II (PKAR)^11,35^ in renal principal cells. PDE4D3 hydrolyzes cAMP to AMP and is, therefore, able to control PKA activation and signal termination. Interestingly, this isoform has an increased catalytic efficiency after phosphorylation at serine 54 by PKA^43^. This configuration allows for a negative feedback loop that was described in a different setup^44^ and will be evaluated in this work. Phosphatase PP2B is able to dephosphorylate AQP2 as well as PKA^42^, which allows the protein to influence the phosphorylation state of both proteins simultaneously. To evaluate the actual phenotypic effect of the signalosome in the whole-cell context, we decided to model the effect of the cascade on exo- and endocytosis systems that regulate the membrane permeability by the accumulation of AQP2. The fact that both transport directions are regulated in response to arginine vasopressin is known through models and experiments^45–48^. How exactly the phosphorylation increases the frequency of exocytosis is not fully understood^49,50^. It seems certain that an intact actin network is required for the transport from storage sites to the apical membrane^51,52^. However, the actin cortex at the apical membrane represents a physical barrier and needs to be “softened" for the vesicle to reach and be incorporated into the membrane^51,53^. Clathrin-mediated endocytosis is able to internalize AQP2-positive vesicles^47,54^, which are transported on microtubules to storage compartments via endosomes^48,55^. AQP2 has become a “model protein" for understanding exocytic and endocytic processes, as well as eukaryotic hormone-regulated signaling mechanisms^33^ (see Fig. 1).

### Models of water permeability regulation in renal principal cells

One of the first models that considered the water permeability of principal cells was presented by Knepper and Nielsen^45^. They considered that the water transporter was in an “active", “inactive", and “reserve" state, and the transition from one state to another requires first-order kinetic reactions. The reaction rates are scaled by the presence of vasopressin and simulated to fit the experimentally measured trajectories. They concluded that vasopressin and by proxy cAMP must regulate both the insertion (activation) and the retrieval (inactivation) of water transporters in order to explain the measurements. In the spirit of this model, describing the phenotype of cell permeability, Fröhlich et al. constructed a more detailed model^56^. The roles of the vasopressin receptor, ACs, PKA, and AQP2 were evaluated. Ordinary differential equations were used to simulate mass action kinetics. Different versions of the model were devised as in silico experiments to assess the importance of the internalization of the vasopressin receptor and the PDE activity, amongst others. Nine reactions were used to describe the most important transitions in the system. They found that either a negative feedback loop during PDE hydrolysis of cAMP or the internalization of VR is required to explain the data. Furthermore, they determined that the parameter for endocytosis had the highest impact on the AQP2 concentration in the membrane. Both models considered the whole cell from vasopressin simulation to AQP2 membrane accumulation, by compromising the mechanistic details of the individual mechanisms. Other models that inspired this work are of more mechanistic nature. Buxbaum and Dudai considered the activation of PKA using two isomorphic cycles^57^. One cycle describes the dephosphorylated form of PKAR and the other describes its phosphorylated version. The addition of cAMP to the system leads to the dissociation of the holoenzyme and the accompanying activation of PKAC. Twelve differential equations were simulated numerically using kinetic parameters from literature, complemented by estimations from the authors. They could recreate experimentally observed trajectories by systematically varying kinetic parameters and evaluating the necessary changes thoroughly. Multiple models exist that consider different cell types and mechanisms of cAMP diffusion^58^. Feinstein et al.^59^ used the Virtual Cell framework to simulate the spread of cAMP in cells, varying multiple physical and biochemical parameters. Diffusion processes and reactions were solved using a finite volume method and differential equations. They conclude that the observed reduction of diffusivity is probably facilitated through high concentrations of cAMP buffers, changes in cytosolic viscosity, and structural impediments. Lastly, we would like to highlight the model of vesicle transport and cytoskeleton by Klann and colleagues^24^. In a general agent-based framework, they model vesicles as well as proteins as agents that are able to move in a 3D environment. Reactions can occur at the membrane surface, the cytoplasm, or inside the vesicle using mass action kinetics solved by a stochastic integration scheme. The resulting vesicle model is able to reproduce vesicle budding, transport, and fusion events determined by the vesicle cargo in a multiscale manner. They acknowledge that the setup is a step towards system-level understanding through a mechanistic approach, and bridging the gap from molecular interactions to cellular phenotype is desirable.

### Motivation and aim

The response of eukaryotic cells to signaling molecules in adjacent tissues is a complex interplay of many molecular systems^60^. However, the integration of all relevant mechanisms in conjunction with their location in the cell into a complete signaling cascade is an intricate undertaking. The modeling process is further complicated by the different scales of time and space. Systems biology is therefore challenged with the integration of mechanistic details and rules from different studies^61^. The modeling of the vesicular transport system is most often tackled by agent-based approaches^24,62,63^ whereas other intracellular transports and signaling phenomena are approached via continuous models^64–66^. Signaling cascades that involve vesicles for intracellular transport include phenomena of both areas. We investigated biological phenomena that are involved in the vesicular recycling in general and in the AQP2 response especially:

- The unique nature of PKA regulation^3^
- Organization of proteins in signalosomes^8^ and biological condensates^67^
- cAMP compartmentalization and its apparent low diffusivity^68^
- Coupling of microscopic and macroscopic signal transduction^69^

We found that all these different aspects are inherently tied together and are key elements to understand the PKA/AQP2 signalosome. In order to address systems that involve both macroscopic and microscopic changes, a hybrid model similar to that of Klann and colleagues^24^ is required. We, therefore, developed an integrated approach that allows for hypothesis-driven modeling of complex signaling pathways. Furthermore, we implemented this approach in a framework that encourages the definition of rule-based reaction- and behavioral systems. We build the models sequentially, starting with the PKA signaling response. Afterwards, the model was extended to include cAMP diffusion and compartmentalization. Finally, endo- and exocytosis, as well as intracellular transport were added. After each step, we evaluated the behavior of each model individually and in conjunction with the previous results. We reviewed literature knowledge of this signaling cascade and found qualitative as well as quantitative building blocks. This allowed us to verify the applicability and parameters of the individual components. Furthermore, we were able to deduce cellular behavior in parts of the model where no literature information could be determined. This knowledge allows suggesting new experiments where research can focus to fill in gaps and approaching whole-cell models in an incremental approach^70^. The ability to encapsulate cellular behavior of different scales into independent modules allowed us to integrate numerous mechanisms and parameters. We devised the most complete model of the vesicular AQP2 transport system to our knowledge, using data from more than 100 published sources. An overview of the integrated aspects can be found in Supplementary Figure 1.

## Results

### Allosteric phosphorylation model

**Fig. 2:**
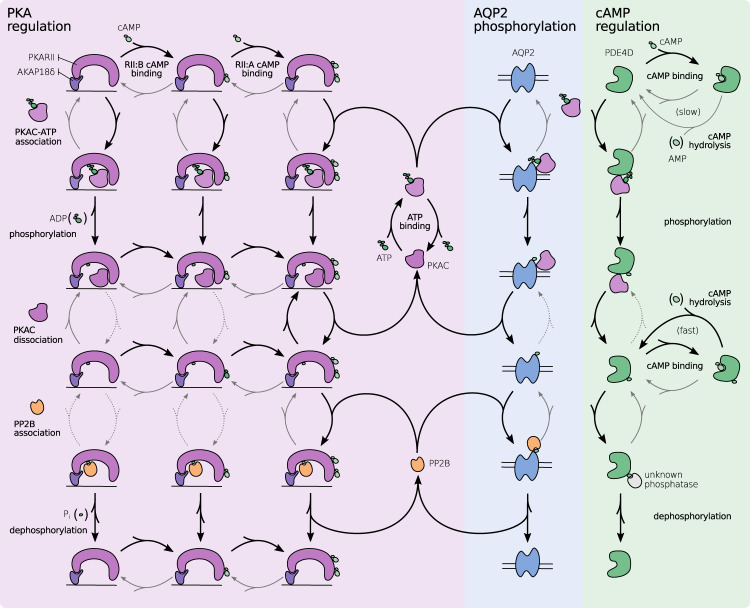
PKA regulation and effect on AQP2 and PDE4 phosphorylation. The network of possible reactions during signal processing as a response to cAMP. Black arrows indicate preferred and fast reactions, gray arrows show slow but still significant reactions, gray and dotted arrows are considered negligible in their frequency. The following reactions in each row from top to bottom: PKA and substrate association, substrate phosphorylation, substrate dissociation, phosphatase and substrate association, and PP2B phosphorylation and release. Substrates are only displayed once and considered implicitly row and column-wise. PKAC is only released upon binding of a second cAMP to the regulatory subunit. A negative feedback loop is present, where the released PKAC phosphorylates PDE4, leading to an increased PDE4 activity which decreases the cAMP concentration. Abbreviations: protein kinase A type II regulatory subunit (PKARII), cAMP binding site A/B at PKARII (RII:A/B), protein kinase A catalytic subunit (PKAC), A-kinase anchoring protein 18 variant *δ* (AKAP18*δ*), Serine/threonine-protein phosphatase 2B (PP2B), aquaporin 2 (AQP2), phosphodiesterase 4D (PDE4D).

The group of Susan Taylor^14,71,72^ systematically explored the mechanisms of PKA activation. New models are already starting to include and explore this mechanistic knowledge^15^. We implemented the most recent model for the activation of PKA and included the subsequent phosphorylation of AQP2 and PDE4 (see Fig. 2). For a detailed description and biological background of the model, see the Supplementary Information Section 1. The most interesting observation in recent years is, that the active subunit of PKA, PKAC, binds to the regulatory subunit PKAR with a high affinity and phosphorylates it. In principal cells, the regulatory subunit that regulated PKA response at vesicles is PKARII*β*^11^. The phosphorylation will prevent the binding of further PKA, but the PKA that phosphorylated the PKAR is trapped in the bound state until two molecules of cAMP bind to PKAR. This leads to an interesting dynamic, where the PKAC/PKAR complex is disbanded, if enough cAMP is available and needs to be dephosphorylated to return to its basal state. Since this model does not include ACs, we modeled a steady cAMP influx to emulate their activation. We explored the reaction parameter space of this phosphorylation model, by observing the phosphorylation ratio of AQP2 and the PKA activity in 5184 different settings for five minutes each.

#### PKA activation is buffered and underlies a positive feedback loop

**Fig. 3:**
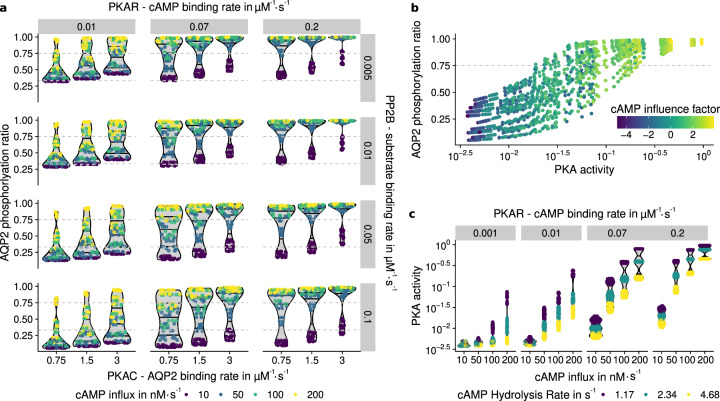
Simulation results of PKA and AQP2 phosphorylation. Simulations with different parameter combinations were run for 5 minutes and the resulting phosphorylation ratios and have been measured. Basal phosphorylation (0.46) and threshold AQP2 phosphorylation ratio (0.75) are shown as gray dashed lines. cAMP influx describes the cAMP concentration entering the system every second produced by adenylyl cyclases. **a** High cAMP influx (indicated by color) is a major determinant for phosphorylation. Low PP2B binding rate (increasing from top to bottom) leads to an increased concentration of PKAC in the simulation and higher phosphorylation rate, whereas a high PKAR-cAMP binding rate (increasing from left to right) leads to a high phosphorylation rate nearly independent of the cAMP influx. Horizontal lines are drawn in the background density estimates at 25%, 50%, and 75% quantiles. **b** cAMP influence factor is calculated by $\log$(cAMP influx ⋅ cAMP binding). High cAMP influence on the simulation leads to high PKA activation and subsequent AQP2 phosphorylation. Physiological parameters for PKA activity should be able to reach AQP2 phosphorylation required (0.75), but still be sensitive to shifts in cAMP concentration. **c** cAMP hydrolysis mediated through PDE4 significantly influences PKA phosphorylation, depending on the cAMP influx and PKAR-cAMP binding rate. Abbreviations: protein kinase A regulatory subunit (PKAR), protein kinase A catalytic subunit (PKAC), serine/threonine-protein phosphatase 2B (PP2B), aquaporin 2 (AQP2), phosphodiesterase 4D (PDE4D).

The simulations show, that PKA activity is highly sensitive to the influence of cAMP (see Fig. 3b). cAMP causes PKAC to be released from its regulatory subunit, and subsequently, free PKAC is able to phosphorylate PKA regulatory subunit (PKAR), reducing its affinity to PKAC^71^. This positive feedback loop increases the amount of unbound PKAC. Since PKAR is available in excess^73^ and two molecules cAMP are required to release PKAC, PKAR is able to buffer some cAMP and the amount of unbound PKAC is initially small. Nevertheless, the buffering of cAMP is not able to significantly alter the process of PKA activation once enough cAMP is available. The process might be required to attenuate volatile cAMP concentration in the cell to inhibit premature activation of the positive feedback loop.

#### Hydrolysis of cAMP decreases PKA activation

Phosphorylation of PDE4 has the possibility to initiate a negative feedback loop by increasing cAMP hydrolysis^43^, subsequently reducing the PKA activation level. In this model and with parameters varying around physiological conditions, a negative feedback loop could be observed. PDE4 activation and hydrolysis rate had a big impact on PKA activity (Fig. 3c), but played a secondary role when considering AQP2 phosphorylation. Further, PKA activity is heavily influenced by the cAMP influx and the PKAR binding rate (see Fig. 3b). About 75% of the AQP2 molecules phosphorylated at S256 are sufficient to trigger the departure of the vesicles to the apical membrane^74^. Therefore, we used this threshold to evaluate whether phosphorylation is sufficient for the propagation of the signal. An influx of 200 nMs^−1^ cAMP resulted in a sufficient phosphorylation ratio in all simulations where cAMP binding rate was 0.07 μM^−1^ s^−1^ or greater.

#### Phosphatase PP2B reverses PKA response

The binding rates of PKAC and PP2B to AQP2 are key factors for AQP2 phosphorylation (see Fig. 3a). High binding rate of PKAC and low binding rate of PP2B lead to an increased AQP2 phosphorylation ratio at similar PKA activity levels. PKAC is able to phosphorylate PKAR, PDE4 and AQP2 in this model and additional targets in vivo. The competitive binding that is possible in this configuration was considered by using two reactions for binding and phosphorylation, essentially trapping a small amount of PKAC in each time step. Decreasing the binding rate of PKAC to AQP2 therefore increases the amount of PKAC available to phosphorylate other components. The variation of the relevant binding affinities was unable to produce an effective difference in PKAC concentration, and has therefore minor impact on PKA behavior. PP2B is indirectly activated by Ca^2+^ via Calmodulin. Since the Calmodulin pathway was not considered in this model, the parameter variation in PP2B binding rate is used as a proxy to determine the influence of Ca^2+^ on the PKA/AQP2 pathway. High PP2B binding rates were able to reduce AQP2 phosphorylation in some models, but high cAMP influx was able to override this effect for nearly all setups (see Fig. 3a).

We demonstrate that the model is able to represent multiple aspects of PKA activation. The excess of PKAR and two cAMP binding sites prevent premature activation. An initial positive feedback loop wherein PKAR phosphorylation reduces PKAC binding leads to a switch-like activation of all phosphorylation targets of PKA. The subsequent negative feedback loop involving PDE4 is able to effectively reduce cAMP concentration and PKA activation. The simulations were performed without spatial components that could prevent cAMP from reaching its destination. In the next models, we wanted to investigate, how cAMP compartmentalization influences the PKA and AQP2 phosphorylation.

### cAMP compartmentalization in the vesicle storage region

**Fig. 4:**
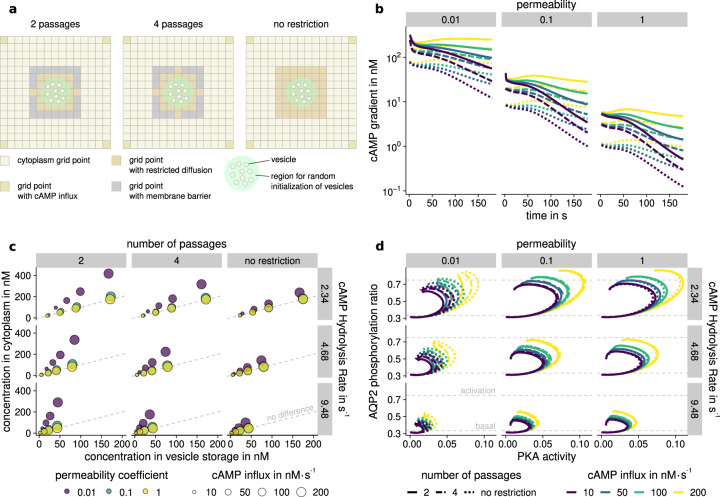
cAMP compartmentalization in different environments. Simulation of cAMP diffusion was performed in different environments and with multiple parameter sets. **a** Schematic representation of restricted environmental setups with and without barriers around a vesicle storage region. **b** cAMP gradient over time, depending on the number of passages (dashed lines) and cAMP influx (color). Simulations without permeability reduction (decrease in diffusivity) are unable to maintain effective gradients of 100 nM or more. **c** Influence of PDE4D hydrolysis rate and a number of passages on concentration in different compartments after five minutes of simulation. Circle size indicates cAMP influx, whereas color shows permeability. Only the combination of diffusive restriction and low permeability was able to create significant differences in concentration, indicated by circles far away from the dashed lines. **d** Shown is the AQP2 phosphorylation ratio and PKA activity development during simulation starting from basal value. After an initial increase, a negative feedback loop first slows and subsequently decreases phosphorylation rates. High PDE4 hydrolysis rate (increasing from top to bottom) leads to lower cAMP concentration in the vesicle region, leading to lower activity. High cAMP influx (yellow lines) allows overcoming the phosphorylation threshold of 0.75. Abbreviations: protein kinase A (PKA), aquaporin 2 (AQP2), phosphodiesterase 4D (PDE4D).

One of the most well known second messengers, cAMP is compartmentalized frequently in signaling pathways^2,75,76^. For this to occur, various chemical and physical prerequisites have to be met^59^. The phenomena that influence the compartmentalization of cAMP can be condensed to three major factors: the hydrolysis of cAMP by PDE, the apparent low diffusivity of the cytoplasm in vivo, and transient or permanent chemical interactions^21,58,76,77^. We modeled and assessed these factors by a variation of kinetic parameters and environmental setup. The reduced diffusivity of cAMP molecules in the cytoplasm can be caused by multiple factors, which we refer to as cytoplasmic permeability or permeability for short. To model this, a base diffusivity of cAMP of 32 μm^2^ s^−1^^22^ is used and scaled in certain regions of the cellular model with the permeability coefficient. Further, the influence of macroscopic objects that block access to the areas where PKA was assessed (see Fig. 4). The hydrolysis of cAMP was explicitly modeled and binding affinities to all relevant components were systematically explored. The detailed model setups and parameter variations are listed in Supplementary Information Section 2. To evaluate the importance of cAMP for the actual regulation of the AQP2 pathway, both, the developing cAMP gradient and the resulting PKA activity need to be considered.

#### cAMP compartmentalization only develops in regions with reduced permeability

The most important factor for the emergence of gradients is permeability (see Fig. 4b). It is possible to observe compartmentalization effects in all simulations. Differences on the scale of 10-fold reduction as suggested by Iancu and colleagues^78^ are only obtained with low permeability and high cAMP hydrolysis rates. Simulations without any obstacles and full permeability were unable to attain a significant cAMP gradient, even when considering highly potent PDE and buffering (see Fig. 4c). With high permeability, the whole system is affected by the cAMP hydrolysis, resulting in a nearly uniform distribution across compartments. Others have also observed these effects in experiments and simulations in neonatal cardiac myocytes^21,79^, dendrites of neurons^77,80^, and other cell types^59,81^. Stephan and colleagues observed cAMP compartmentalization in renal principal cells^82^. Since cAMP is produced at the basolateral membrane and vesicles are stored close to the apical membrane, cAMP needs to travel through the cytoplasm. It seems feasible that a locally reduced cAMP concentration is required to prevent premature activation of PKA as a response to the basal cAMP concentration^19^. After the cellular concentration of cAMP increases due to activation by vasopressin, the actual response pathway is triggered. The required components of the signalosome are all coupled to AKAP18^41,42^.

#### Buffering effects of AKAP bound PKAR play a minor role in compartmentalization

Buffering was proposed to have an effect on cAMP compartmentalization. cAMP buffering is the capacity of proteins and other cAMP binding molecules to temporarily or permanently fixate cAMP rendering it unavailable in the pool of free cAMP. In this setup, buffering is mostly performed by PKAR subunits. Since PKAR is available in excess, it is able to bind a significant amount of cAMP (twice the concentration of PKAR in the system)^73^. PDE4 buffers a negligible amount just before catalysis. As discussed in the phosphorylation model, buffering is able to create short-term reduction of the cAMP and therefore fine-tune and stabilize small fluctuations in the sink. However, binding to PKAR is also required to activate PKAC. Therefore, cAMP buffering at PKAR can not be viewed as a means to generate cAMP gradients that result in a specialized response. PKAR and PDE4 are only a part of the possible binding partners of cAMP. It would be interesting to analyze the specific and unspecific binding of cAMP to gain more insight as to how unspecific buffering contributes to compartmentalization.

#### The creation of cAMP sinks is a delicate balance between multiple factors

PDE4 degrades cAMP in the cell. PDE4 in the PKA signalosome can be activated by PKA, which increases its cAMP hydrolysis rate^43^. A high hydrolysis rate leads to lower cAMP concentrations close to the vesicles. The creation of cAMP sinks is a delicate balance between the amount of cAMP that is produced, the turnover rate of PDE, and the reduced access of cAMP to relevant regions of the cell (see Fig. 4c). Physiologically, increasing the cAMP influx seems inefficient, since large amounts of energy would be required to produce cAMP, only to degrade it moments later. The amount of PDE4 required to decrease cAMP influx without diffusive restriction would far exceed physiological ranges^21^. This can be confirmed by our simulations. The cAMP influx is not sufficient to compete with the degradation by PDE4 for permeabilities of 0.1 or higher. A high diffusive restriction in the vesicle area is an elegant solution to compromise both factors. Fewer cAMP molecules reach the vesicles and whenever the influx exceeds the degradation capacity of PDE, it results in activation of PKA. Other factors gain influence, if the permeability is 0.1 or less. We used the phosphorylation ratio of AQP2 to determine, under which conditions, cAMP compartmentalization is able to influence signaling. We found that phosphorylation levels of AQP2 surpass the 75% threshold, if the cAMP hydrolysis rate is low enough and cAMP influx is sufficiently high (see Fig. 4d).

In conclusion, the prime factor to create effective cAMP compartmentalization was the permeability of the vesicular storage. Hydrolysis of cAMP by PDE4 is able to fine-tune the concentration of cAMP and regulate the signaling response. The explicit buffering modeled in this study did not contribute significantly to sustainable compartmentalization.

### Clathrin-mediated endocytosis model

In the basal state of the cell, vesicles are located in the storage region until they are transported to the membrane for fusion. New vesicles are created at the apical membrane via clathrin-mediated endocytosis, depending on SRC phosphorylation^54,83^. A constantly shifting imbalance of exocytosis and endocytosis is the major driver behind the water reabsorption of principal cells in the kidney. An increased endocytosis shifts the majority of AQP2 to the storage region, whereas increased exocytosis leads to high AQP2 concentrations in the apical membrane. The increase in exocytosis is mediated by the pathways we explored in the previous models. The frequency and regulation of endocytosis is the focal point of the endocytosis model.

#### Endocytosis is mediated by PKA activation and AQP2 concentration

Endocytotic pits, the precursors of clathrin-coated vesicles, emerge spontaneously on the apical membrane surface and the maturation from pit to vesicle is correlated to key cargo molecules^84^. We determined the rate of endocytotic pit formation as well as the cargo collection speed, from the approximate number of AQP2 molecules per vesicle^85^ and parameter variation. Furthermore, we implemented a regulation mechanism involving Non-receptor tyrosine kinase Src (SRC) to model the AQP2 accumulation observed in active cells^83^. The model to evaluate the parameters for this process imitates an apical membrane section with a surface of 1 μm^2^ that contains the expected molecules after cAMP stimulation. Knowledge from the phosphorylation and compartmentalization models was used to constrain the parameters of the endocytosis model. The detailed model setups and parameter variations can be reviewed in Supplementary Information Section 3.

#### Pit emergence and cargo addition are coupled parameters

**Fig. 5:**
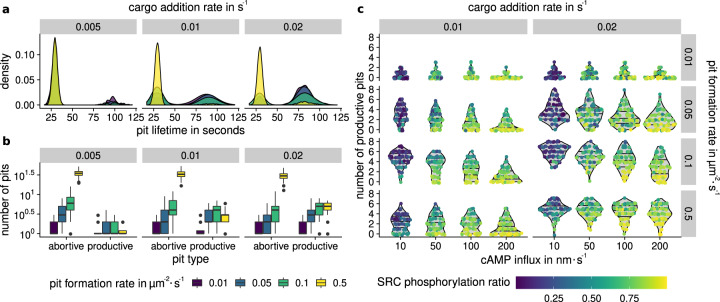
Distribution of abortive and productive endocytotic pits. **a** Lifetime of endocytotic pits across all simulations at varying cargo addition rates. The first peak indicates abortive pits that did not enter the maturation phase, the second peak indicates pits that matured to vesicles. **b** The number of abortive and productive pits after five minutes. High cargo addition rate leads to high number of productive pits. After a substantial amount of AQP2 molecules are transferred to vesicles, no new productive pits are able to form. The central line represents the median, lower and upper box boundaries correspond to the first and third quartiles (the 25th and 75th percentiles). The whiskers extend to the largest/smallest value, no further than 1.5 time the interquartile range. Data beyond the end of the whiskers (outliers) are plotted individually. **c** Number of productive pits at varying parameters, colored by average SRC phosphorylation ratio. Low cAMP influx leads to higher number of productive pits, given a sufficient pit formation rate. Horizontal lines are drawn in the background density estimates at 25%, 50%, and 75% quantiles. High SRC phosphorylation ratio results in fewer productive pits. Abbreviation: non-receptor tyrosine kinase Src (SRC).

A low cargo addition rate results in a high number of abortive pits, since the threshold for successful maturation, cannot be reached in time (see Fig. 5a). Furthermore, high pit formation rates lead to a high number of abortive pits (see Fig. 5b). There is an upper threshold to the number of productive vesicles that are able to form, imposed by the total number of AQP2 in the membrane. After a significant amount of cargo has been transferred to vesicles, no more productive pits are able to develop. If many pits form in parallel, the available cargo molecules are distributed across all pits in the membrane. Hence, not enough cargo can be accumulated in each pit and the number of productive pits remains low.

#### Model of SRC inhibition is able to reduce productive pit count

In this model, the ratio of inhibited and active SRC was used to scale the cargo accumulation rate, such that inhibited SRC leads to an increase in abortive pits. The inhibition of SRC is mediated by phosphorylation of tyrosine 527, which is performed by activated C-terminal SRC kinase (CSK)^86^. CSK itself can be activated by PKA via phosphorylation of Serine 364^87^. Therefore, activation of PKA leads to an inactivation of SRC and subsequently to AQP2 retention in the apical membrane. The influence of SRC phosphorylation can be seen in Fig. 5c. The decrease in effective cargo collection rate resulting from SRC phosphorylation is able to qualitatively reproduce the observations made by Cheung et al.^83^. A high cAMP influx rate is associated with fewer productive pits. Inversely, a high number of productive pits can be observed in systems with a low cAMP influx, as a result of inactive PKA and dephosphorylated SRC.

### Full recycling model

For the recycling model, we use the previous sub-models to set up a spatiotemporal model of the vesicular recycling system of renal principal cells. The model represents a subsection of the cell that includes a vesicular storage region as well as the apical cell membrane. The processes and reactions implemented in this model are detailed and visualized in Supplementary Information Sections 4 and 5.

**Fig. 6:**
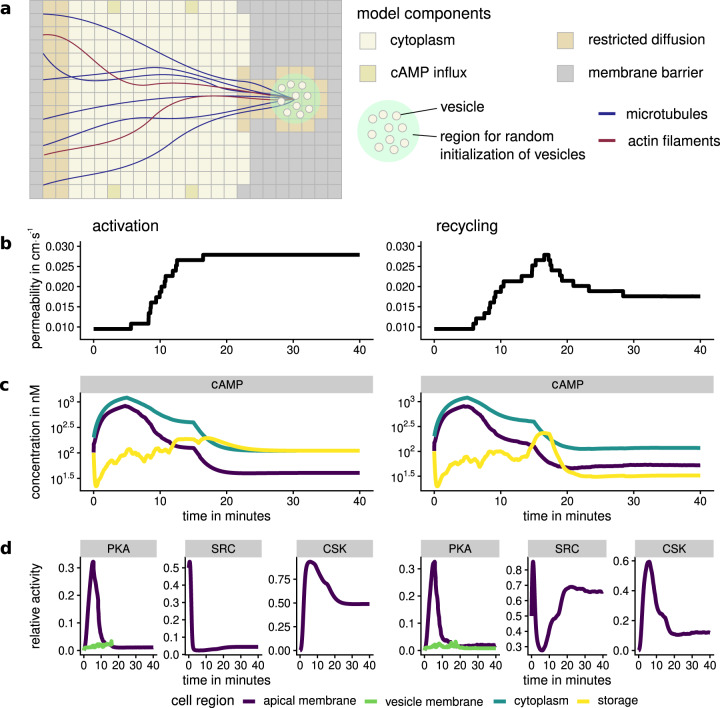
AQP2 transport activation and recycling. Two setups of the AQP2 transport full model showcase 40 mins of activation (left) in opposition to recycling (right) when using accelerated SRC and CSK dephosphorylation. **a** Schematic representation of the environmental setup. The perinuclear storage region contains AQP2-positive vesicles that are transported to the apical membrane along actin filaments. Endocytosis is able to form productive pits in the recycling model, leading to vesicles that are transported to the storage region. **b** Membrane permeability estimated from AQP2 concentration in the apical membrane. Permeability increases and stays high if endocytosis is suppressed. **c** cAMP concentration in different cell regions. cAMP influx decreases during simulation. Free cAMP in the storage region is quickly buffered and able to active PKA, leading to AQP2 phosphorylation and vesicle departure. **d** Relative activity levels of selected phosphorylation targets. PKA shows similar activation behavior in activation and recycling models. Faster reactivation of SRC after inactivation by the PKA/CSK/SRC cascade recovers ability to produce productive pits. Abbreviations: protein kinase A (PKA), aquaporin 2 (AQP2), non-receptor tyrosine kinase Src (SRC), C-terminal SRC kinase (CSK).

As our previous models have shown, diffusion-restricted regions are required to achieve cAMP compartmentalization. Therefore, two regions are defined that reduce the diffusivity of cAMP (see Fig. 6a). Agent-based modeling is used to simulate the dynamic behavior of vesicles. Two exemplary setups are used to evaluate the behavior shown by the model: activation and recycling. The first activation model exhibits behavior close to that of natural principal cells. In the recycling model, SRC and CSK dephosphorylation have been increased, which allows for observing exocytosis as well as endocytosis in action.

Both models exhibit a distinct increase in exocytosis. Vesicles are able to move along actin filaments to the apical region of the cell whenever the phosphorylation threshold is reached. Once the vesicle is in proximity to the apical membrane, fusion commences^88^. The initiation of this cascade is regulated by PKA, as demonstrated in previous results. The discrete increases in permeability (see Fig. 6b) are the result of individual vesicle fusion events. cAMP produced by ACs is stimulated by cAMP influx at nodes indicated in Fig. 6a. In both models, cAMP is created at 400 nMs^−1^ for each of the four cAMP influx grid points for the first 5 minutes and at 200 nMs^−1^ up until 15 minutes. For the rest of the simulation, a basal cAMP influx of 50 nMs^−1^ is assumed. Initially, cAMP concentration in the cytoplasm and at the apical membrane increase steadily (see Fig. 6c), and the amount of active PKA rises in conjunction. cAMP in the storage region fluctuates significantly, driven by movement and departure of vesicles. After an initial decrease where cAMP binds to PKAR and PDE4 in large amounts, the average cAMP concentration increases slowly. In this model, the access to the vesicular storage region is decreased by membranes, whereas the apical membrane is more exposed. The resulting effect is an effectively decreased cAMP hydrolysis whenever a major part of the PDE4 concentration is in the storage region. Less cAMP is able to reach PDE4 and fewer cAMP production is required to keep basal cAMP levels stable. Vice versa, the exposure of PDE4 to cAMP is increased whenever vesicles at the apical membrane, decreasing the cellular cAMP stores faster. This mechanism increases the effectiveness of the negative PDE4 feedback loop already present in the initial phosphorylation model and was not explicitly implemented in the model. This is further underlined by the quick deactivation of PKA in the apical membrane, even with significantly increased cAMP concentration (see Fig. 6d).

In the recycling model, PKA and SRC phosphorylation rise similarly as in the activation model. The increased CSK and SRC dephosphorylation lead to a quick reversal of SRC activity. The increased concentration of AQP2 in the membrane, as well as the reactivation of SRC, lead to an increased endocytosis rate. Vesicles are then transported to the storage region using microtubule-based transport^48^. In the activation model with slower dephosphorylation rates, endocytotic events only produce aborted pits and the concentration of AQP2 in the membrane stays constant.

#### Increase in exocytosis and decrease in endocytosis are entwined

cAMP influences not only the exocytosis of AQP2-positive vesicles, but also their endocytosis. In the basal state of the cell, the majority of AQP2 is kept in storage compartments in the center of the cell. A two-pronged approach ensures that the effort of transportation is effectively utilized. The maturation of endocytotic pits is reliant on the cargo concentration in endocytotic pits, hence an increase in AQP2 in the membrane leads to an increase in endocytotic events. The regulation mechanism in place is modeled by SRC inhibition. The actual quantitative influence of SRC to inhibit pit maturation is speculative. It would be interesting to investigate the actual kinetic influence SRC has on pit maturation and/or vesicle scission experimentally. Other mechanisms have been identified that can contribute to the decrease in internalization^89^, therefore it is unlikely that SRC is the only determinant for the inhibition of endocytosis.

#### Molecular condensates can explain localized responses

The diffusive restriction of cAMP and coherence of all molecular components are requirements for a regulated and distinct signal response. The diffusive reduction falls within a regulated margin: if no reduction is present, cAMP renders PKA always active, even at basal cAMP levels. Since endocytosis is also reliant on indirect cAMP-based activation of SRC kinase, the system transitions to a state where AQP2 concentration in the apical cell membrane is always high. On the other hand, systems with high restriction are slow to respond to signals, since few cAMP molecules are able to reach the cAMP storage region at a time, delaying PKA activation. Furthermore, AQP2 does not remain in the membrane, since endocytotic events occur frequently. The decrease in diffusive restriction would therefore be associated with chronically increased membrane permeability, whereas increased restriction would lead to polyuria. All components associated with the AQP2 response are associated with a PKA signalosome. If any of the components would remain in the storage region or at the apical membrane, they accumulate and alter the signal response over time.

#### Deviations between model and phenotype

The original response of principal cells is mostly measured by the water permeability of the apical membrane^90,91^. Therefore, we converted the concentration of AQP2 to permeability as described in Supplementary Table 15. The majority of the activation happens in the first minutes. This is consistent with the previously observed mode of PKA activation, where a positive feedback loop is present. The measurements of Deen and colleagues^91^ show that permeability doubled after 10 mins and tripled after about 30 mins when compared to the basal rate. We also observe these ratios, but the measured progression in experiments is closer to a linear gradient. This derivation may have multiple potential origins, of which we will address three that seem the most probable to us. The model describes one storage area and subsection of the cell. Measurements in experimental data were not taken from a single cell, but from cell culture. Potentially, multiple storage sites that are triggered at different times can contribute to a more evenly distributed permeability increase. Furthermore, we are starting the simulation with vesicles that contain a uniform number of molecules. It is probable that the vesicular response is altered depending on whether the vesicle was recently recycled or in storage for some time^31^. Additionally, we did not model all the aspects that play a role in the vesicular trafficking, for example, the role of Ca^2+^, the other phosphorylation sites distinct from S256, and different AKAPs have an influence on the recycling and alter the specific response^92^.

## Discussion

We combined differential equations and agent-based modeling to gain insight into the vesicular recycling system of renal principal cells. Both approaches complement each other by modeling different aspects of the vesicular system. Especially, the combination of microscopic and macroscopic aspects of clathrin-mediated endocytosis requires the combination of chemical reactions and agent-based modules. Additionally, it was required to work out a viable model of clathrin-mediated endocytosis, extend existing models of PKA regulation, and estimate parameters to link the involved processes.

### Modeling and simulation approach

Vesicular transport is a key cellular activity responsible for molecular traffic between membrane-enclosed compartments and outer membranes. Vesicles are the cell’s solution to directed transport that was necessary to reap the benefits of eukaryotic size and complexity. It is however difficult to consider the interplay between biochemical reactions and diffusion processes and large membrane-enclosed compartments. The diameter of intracellular vesicles is between 30 nm and 100 nm^93^, on the order of 10 times larger than typical proteins, and even 100 times larger than small molecules^94^. Furthermore, the physicochemical processes occur at different scales of organization. While vesicles diffuse at 0.13 μm2 s^−1 95^ the important second messenger cAMP has a diffusion coefficient of ~32 μm2 s^−1 22^. In order to integrate the different scales of organization in a single model, simulation of the different components has to be addressed with multiscale modeling and simulation systems. The hybrid model devised in this work allows for defining agents as well as reactions in a hypothesis-driven approach. The continuous modules of the model are calculated on a “reservoir"-like grid using mass-action kinetics. Diffusion of chemical entities between the reservoirs is possible. Agents such as vesicles and membranes are added as a second layer to the model. Agents can also contain reservoirs that are able to exchange information and react with the nearby underlying grid. Whenever agents move, the overlapping regions of the agent and grid are calculated and updated. The numerical error resulting from the calculations is observed on a per-reaction basis as well as for the whole system. This allows determining critical reactions that govern the time step size in an adaptive step width approach. The resulting adaptive system considers smaller time steps during critical periods of the simulation and speeds up if numerical errors are negligible. The computational cost, therefore, scales with the fastest reaction in the system (requiring the most time steps to compute to a sufficient accuracy). The computation of one real-time hour of the full recycling model with a single parameter set requires about 100 hours of run time on a desktop computer with an 8-core CPU and 16 GB of RAM. Naturally, the framework presented in this work is not as refined as established approaches, it is however capable of capturing the diverse aspects of vesicular transport and signaling.

### Insights into the AQP2 recycling model

We categorized the major findings resulting from the models into the following three groups: support for previous findings, new insights, and topics that remain to be explored.

#### Support for previous findings

Our model confirms that PKA activity can be tightly regulated by a controlled local association and dissociation of PKAC and PKAR controlled by cAMP as suggested by Zhang et al.^71^. We verified that PKA needs to be tethered to the vesicle and to the other components of the signalosome for it to have a distinct effect^73^. Components that are not bound to the vesicular storage region diffuse into the cell and render the response unspecific. The PKA signalosome plays a role in multiple cellular processes, depending on its composition^8^. In renal principal cells at least AKAP18, PDE4, PKAR, PKAC, and PP2B are essential for the cellular response and the formation of condensates seems an important factor in reducing the permeability of cAMP.

The phosphorylation of PDE4 initiates a negative feedback loop^44^ in order to restore basal PKA activity. It is being debated if the pathway via cAMP and Ca^2+^^96,97^ can be triggered independently and result in comparable AQP2 distributions^83,98,99^. We argue that there must be some degree of overlap between the responses since PP2B is also regulated by Ca^2+^ indirectly and that they modulate the reaction in different complementary ways. The fact that both secondary messengers are connected is known^100^. Nevertheless, two largely uncoupled pathways allow for the regulation on different timescales and/or use cases.

We confirm, that PDE4 concentrations or affinities would need to be beyond physiological ranges^21,81^ also when viewed in conjunction with the PKA pathway in order to be effective for the generation of CAMP gradients. It turned out that cytoplasmic permeability and, as a result, the ability of cAMP to reach PDE in the PKA signalosome, is the most critical component that allows for the buildup of cAMP gradients. Nevertheless, no effect in isolation is able to create CAMP gradients; the interplay of all effects is required to regulate this phenomenon. The permeability required to create gradients was nevertheless higher than expected. While some gradient can be observed for all permeabilities, effective compartmentalization (on the scale of 10-fold diffusivity reduction) was only consistently obtained for permeabilities ≤0.01. Effects that lead to a diffusive slowdown can be the result of the cytoplasmic matrix, cellular crowding, and weak binding interactions^18,101,102^. Whether these factors are able to create another order of magnitude difference in effective permeability is debatable. A promising explanation comes in the form of liquid phase-separated compartments, also known as biological condensates^67^. Biological condensates have the ability to reduce diffusion^103,104^ of the involved components. Molecules that experience weak and strong binding effects tend to cluster together and promote phase separation. These binding effects can be observed for the majority of proteins involved in the phosphorylation cascade^8,41,42,73,105^. Our study supports this view, which challenges the textbook model of local degradation of cAMP as the major driver of compartmentalization. Furthermore, it could explain the substantial difference in cAMP required for the activation of PKA in vivo vs in vitro^19^.

The regulation of either endocytosis or exocytosis has little effect on AQP2 accumulation. Both pathways are self-regulating to an extent, and only the combination of increased exocytosis and decreased endocytosis is able to initiate a distinct response^45^.

#### New insights

The modeling and simulation process unveiled that PKAR excess decreases substrate specificity^106^, simply by reducing the probability of PKAC encountering and engaging with other substrates, which could contribute to the apparent 10-fold reduction of the activation constant^78^. In connection to this, we also found, that different affinity for PKA phosphorylation targets PKAR, PDE4, and AQP2 had no significant impact on the signal response. Using high PP2B activity as a proxy for Calcium influence indicates that Ca^2+^ does not significantly alter the course of the PKA/AQP2 phosphorylation pathway as well. While the PP2B lowered the basal phosphorylation rate of AQP2, it was not able to effectively counteract the PKA response. The buffering effects of PKAR bound to the AKAP complex are negligible for long-term gradient generation and only able to attenuate small fluctuations of cAMP.

All three factors influence the compartmentalization of cAMP slightly differently (see Fig. 4c): The cAMP hydrolysis rate mainly affects the cAMP concentration in the vesicle region. The diffusive reduction maintains the cytoplasmic concentration of cAMP, and the cAMP influx impacts both cytoplasm and storage compartments. This allows for largely independent control of different spatially distinct cAMP sinks in the cell by using different variants and concentrations of the components that are part of the signalosome. As a result, the combination of components in each cell type is able to determine the specificity and diversity of responses. Furthermore, the apparent efficiency of cAMP hydrolysis is decreased whenever PDE4 is in the storage region. The transport of the PKA signalosome in conjunction with the vesicles exposes PDE4 to the cytoplasm and apical membrane, where PDE4 is exposed to higher concentrations of free cAMP. This increases the effectiveness of the negative PKA feedback loop. This activation of the universal kinase PKA leads to a distinct response, whereas the downregulation can be regulated by more specific components such as CSK and SRC.

The cargo addition rate to an endocytotic pit as well as the emergence of new pits needs to be balanced to support the formation of productive pits. The cascade of PKA/CSK/SRC phosphorylation proved to be a suitable model to inhibit AQP2 vesicle endocytosis and subsequent accumulation of AQP2 in the apical membrane.

#### Unresolved phenomena

The influence of the Calmodulin pathway and the concrete role of Ca^2+^ remain elusive. We speculate, that cAMP is responsible for the activation of the PKA-based response and Ca^2+^ is able to counteract it through PP2B, as well as trigger it independently. The modeling of Ca^2+^ would encompass cAMP producing ACs, that is inhibited by Ca^2+^^107,108^, as well as Calmodulin^96,109^, and Myosin^110^. Another pathway involving Short transient receptor potential channel 3^111^ is a promising candidate to explore for apical AQP2 accumulation.

How exactly the apparent low cytoplasmic permeability close to the vesicle storage region is maintained is not fully understood. Biological condensates seem to play a major role in creating these phase separation effects. Very recently, this phenomenon was found to be critical for PKA regulatory subunit RI*α*^5^. Even if in our setup PKARII*β* was unsuitable to efficiently regulate cAMP buffering, the different treatment of cAMP diffusion close to vesicles was necessary and lead to distinct PKA activation.

It is becoming more clear that the isolated observation of components in vitro can be a bad proxy for their actual behavior in vivo^19^. With the concept of biological condensates in mind, the determination of the individual components' rate constants is not enough. It is, therefore, crucial to determine the influence of “signalosome partner proteins" to create reliable models. The models lead to the conclusion that the phenomena of cAMP compartmentalization and PKA activation are tightly coupled and need to be viewed in conjunction.

Even though this model includes major aspects of the AQP2 transport pathway, it is by no means complete. During the modeling process, we encountered processes that provide substance for further research: Experimental evidence is required for the regulation of vesicle maturation during endocytosis in the context of signaling cascades. How does the cargo concentration influence the vesicle maturation process^52^? How do Src^83^ and Sipa1l1^89^ contribute to the membrane accumulation of AQP2? This and other questions can aid the incremental formulation of a whole-cell model of renal principal cells.

## Methods

The modeling and simulation of cellular spatio-temporal systems involve five major aspects:

- The microscopic components,
- the distribution of those components,
- the macroscopic components,
- the location of those components, and
- the definition of their behavior.

**Fig. 7:**
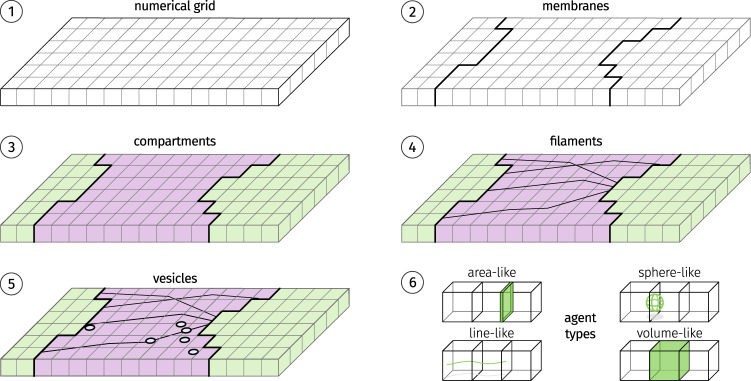
Components of the simulation. First, the simulation space (**1**) is tiled into a regular grid, and used for numerical calculation and spatial indexing. Next, the membrane agents (**2**) determine the compartments (**3**) of the simulation. Each cell of the grid is assigned a concentration of chemical entities. Finally, filaments (**4**) and vesicles (**5**) are placed in the simulation. All agents are implementations of abstract agent types (**6**).

Although, the actual components and their behavior differ depending on the system that is to be modeled, the general nature of the components remains the same throughout all cellular systems. We have developed a framework that abstracts the notion of microscopic and macroscopic components and their interactions. This allows the user to focus on modeling the biological system. The general setup of the simulation system is depicted in Fig. 7.

### Module-based update system

The simulation integrates changes over time that is calculated by independent components called modules. A module can be either concentration-based, displacement-based, or qualitative. Concentration-based modules determine changes in concentrations of chemical entities resulting from reactions and transport processes. Displacement-based modules are used to move agents inside the cell. Qualitative modules can implement rules and algorithms, for example deciding whether a vesicle should be attached to a filament or when and how vesicles fuse with membranes.

**Fig. 8:**
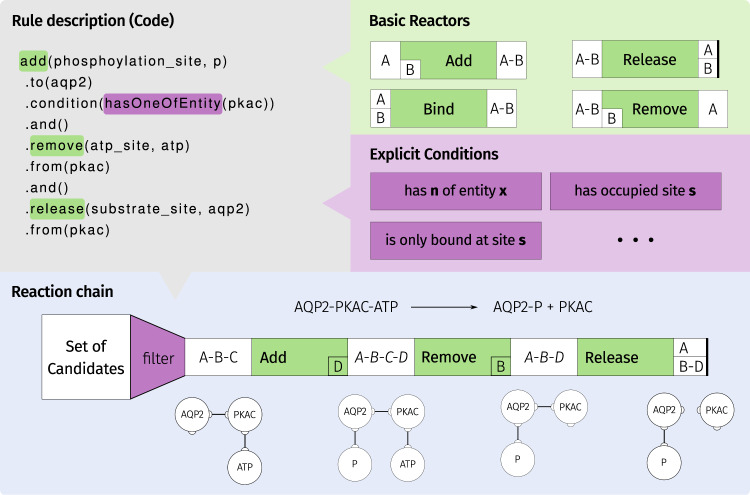
Definition of reaction rules. Reactions can be specified by a description of the reaction process. Basic reactors are concatenated to chains that are processed during the reaction network generation. Additionally, conditions allow for further specification of the reaction process. The network generation process results in all reactions that can occur in the system.

The reactions between chemical entities are defined using a rule-based system. The definition of reactions uses a combination of modification operations: binding, release, addition, or removal. The modification operations basically describe which parts of a chemical entity are added or removed by a reaction. Additionally, criteria can be defined that narrow the amount and kind of chemical entities that are able to react (see Fig. 8). A network generation algorithm determines all possible reactions that can occur with the defined rules and return them to the user for possible refinement. The result of the network generation is a set of ordinary differential equations. Each equation represents an elementary chemical reaction that follows the law of mass action. The behavior of each compartment is determined by these equations that define the concentration change of each chemical entity. Additionally, transport processes use the concentrations of multiple adjacent compartments to determine a change in concentration^112^.

### Timescale optimization and error estimation

The sum of changes for each entity and compartment is determined by the sum of reactions that affect a chemical entity. A local numerical error is calculated on a per-reaction basis. The error is used to decrease or increase the time step, and therefore determines the trade-off between accuracy and speed of the simulation. The evaluation on reaction level allows for the determination of reactions with high numerical error, which in turn allows for their individual optimization until a stable time step is found. After all local errors are acceptable, a total numerical error is calculated that evaluates the change on the chemical entity level.

Displacement-based modules are implemented using a similar approach. The maximal displacement should be small, since abrupt compartment changes for vesicles can lead to instability in the numerical computations. Therefore, the maximal displacement is set to a fraction of the numerical grid step width. A local displacement per module and a total displacement per vesicle are calculated. If the displacement is too large, the time step is decreased, if it is comparably small the time step can be increased. Quantitative modules are able to implement functions that evaluate the error of the function and are equally able to request time step decrease and increase from the simulation.

The modular system in combination with individual error management allows for an extensible framework that enables the simulation of multiscale cellular systems coupling macroscopic and microscopic environments.

### Definition of microscopic components

We use the term chemical entity to refer to chemically distinct species of molecules (according to Systems Biology Markup Language (SBML) terminology). Chemical entities are structured objects that are the source and the result of binding, release, addition, or removal processes performed by chemical reactions. Chemical entities can be represented by a graph structure, where the nodes are also chemical entities and edges are covalent or non-covalent connections between them. The smallest possible chemical entity is a single node that might represent the regulatory subunit of PKA or the messenger molecule cAMP. Using a rule-based definition, chemical entities can be combined to create more complex ones, a process that provides both: the possible reactions and their complex reactants.

The simulation system is subdivided into grid points and further into compartments. The change in concentration *dc*/*dt* is determined by modules, which encapsulate behavior of a certain aspect of the model. Each reaction and transport process that affects chemical entities is encapsulated in a single module. Furthermore, the behavior of macroscopic agents is determined by displacement-based and qualitative modules.

#### Diffusion

The diffusion between compartments is a discretized form of Fick’s Second Law of Diffusion. In the previous work^112^, diffusion was discretized for uniform grid and time steps. Here, the module is adapted to take into account non-uniform reaction spaces and adjustable time steps. Additionally, diffusivity should be reducible in a subregion of the model.

The first of Fick’s laws describe the flow *J* proportional and opposite to a concentration gradient ∂*c*/∂*x*.

1 $$J=-D\frac{\partial c}{\partial x}$$

The diffusion coefficient *D* describes the intensity of the dispersion, due to Brownian motion. The second law describes the spatial and temporal development of a two-dimensional diffusive system,

2 $$\frac{\partial c}{\partial t}=D\left(\frac{{\partial }^{2}c}{\partial {x}^{2}}+\frac{{\partial }^{2}c}{\partial {y}^{2}}\right)=D{\nabla }^{2}c,\quad x\in (0,{L}_{x}),y\in (0,{L}_{y}),t\in (0,T)$$

where *c* is the concentration of a chemical entity at a point (*x*, *y*) and time *t*.

Let

3 $$c(x,y,0)=I(x,y)$$

be the initial-value problem, where the concentration *c*(*x*, *y*, 0) is defined by a prescribed function *I*(*x*, *y*). The domain of the system Ω is of rectangular shape with the following boundary conditions:

4 $$J(0,0,t)=J({L}_{x},0,t)=J(0,{L}_{y},t)=J({L}_{x},{L}_{Y},t)=0\quad \forall t \,>\, 0.$$

The domain is discretized on a uniform Cartesian grid with step width Δ*s*. The set of all grid points is given by

5 $${{\Omega }}:=\{(x,y)\in {{\Omega }}:x/{{\Delta }}s\in {\mathbb{Z}}\ \,{{\mbox{and}}}\,\ y/{{\Delta }}s\in {\mathbb{Z}}\}$$

Let Γ ⊂ Ω be the set of non-diffusible git points, such that *J*(*x*, *y*, *t*) = 0 ∀ (*x*, *y*) ∈ Γ. For each grid point, a restriction coefficient *r*_*i*,*j*_ is defined, which describes the attenuated movement of chemical entities through the region described by the grid point.

Using a forward difference in time and central difference in space Equation (2) is approximated using a five-point stencil:

6 $$(x-{{\Delta }}s,y),(x+{{\Delta }}s,y),(x,y-{{\Delta }}s),(x,y+{{\Delta }}s)\in {{\Omega }}.$$

Moreover, ${c}_{i,j}^{n}$ denotes a mesh function that estimates *c*(*x*_*i*_, *y*_*j*_, *t*_*n*_), such that the discretized function can be written as,

7 $$\frac{{c}_{i,j}^{n+1}-{c}_{i,j}^{n}}{{{\Delta }}t}:=\frac{D}{{{\Delta }}{s}^{2}}\left(\underbrace{{r}_{i-{{\Delta }s,j}} \cdot ({c}_{i-{{\Delta }}s,j}^{n}-{c}_{i,j}^{n})}_{\begin{array}{c}{\rm{left}}\end{array}}+\underbrace{{r}_{i+{{\Delta }s,j}} \cdot ({c}_{i+{{\Delta }}s,j}^{n}-{c}_{i,j}^{n})}_{\begin{array}{c}{\rm{right}}\end{array}}+\underbrace{{r}_{i,j-{{\Delta }s}} \cdot ({c}_{i,j-{{\Delta }}s}^{n}-{c}_{i,j}^{n})}_{\begin{array}{c}{\rm{up}}\end{array}}+\underbrace{{r}_{i,j+{{\Delta }s}} \cdot ({c}_{i,j+{{\Delta }}s}^{n}-{c}_{i,j}^{n})}_{\begin{array}{c}{\rm{down}}\end{array}}\right)$$

and finally

8 $${c}_{i,j}^{n+1}={c}_{i,j}^{n}+\frac{D{{\Delta }}t}{{{\Delta }}{s}^{2}}f({t}_{n},{c}_{i,j}^{n})$$

where $f({t}_{n},{c}_{i,j}^{n})$ denotes the operator that estimates the central difference at ${c}_{i,j}^{n}$ in space and time *t*_*n*_.

#### Reaction kinetics

The dynamic behavior of chemical entities in the system is further determined by chemical reactions. The equations are applied at every grid point, depending on the current concentrations at every time step. The ordinary differential equations are derived from the law of mass action. The law of mass action describes, that the rate of the reaction is directly proportional to the product of the activities of the substrates. The reaction order is defined by the number of concentrations that influence the reaction rate. In general, the resulting reaction is denoted as:

$${{{\rm{A}}}}\mathop{\to }\limits^{{{{\rm{k}}}}}{{{\rm{P}}}}$$

the appropriate reaction rate *v* is described by:

9 $$v=-\frac{1}{a}\cdot \frac{dc(A)}{dt}=\frac{1}{p}\cdot \frac{dc(P)}{dt}=k\cdot c(A)$$

where the stoichiometric coefficients are described by the lowercase letter of the entity and *dc*/*dt* is its change in concentration. Generally, the resulting reaction rate is experimentally determined using a rate constant *k* whose unit is specified by the reaction order. In principle, all chemical reactions are reversible. Many reactions reach a state of equilibrium, where the rate of production of new products is equal to the rate of products degrading to substrates. The following rate reaction scheme describes this kind of reaction:

$${{{\rm{A}}}}\mathop\rightleftharpoons\limits_{{k}_{-1}}^{{k}_{1}}{{{\rm{B}}}}$$

Here, the actual reaction rate is a combination of a forward reaction rate *k*_1_ and a backward reaction rate *k*_−1_. Therefore, the rate equations are as follows:

10 $$v(A)=\frac{dc(A)}{dt}=-{k}_{1}\cdot c(A)+{k}_{-1}\cdot c(B)$$

11 $$v(B)=\frac{dc(B)}{dt}={k}_{1}\cdot c(A)-{k}_{-1}\cdot c(B)$$

where *v*_*A*_ = − *v*_*B*_. Whenever the backward or forward reaction rate is negligibly small, the reaction may be assumed to be irreversible and treated with the kinetics of nth order. A special case for this treatment is the Michaelis–Menten rate equation for enzyme kinetics.

The Michaelis–Menten kinetics assumes that the first step of the reaction of an enzyme and a substrate forms an enzyme-substrate complex according to the law of mass action. Furthermore, the subsequent second reaction is assumed to be effectively irreversible.

$${{{\rm{E}}}}+{{{\rm{A}}}}\mathop\rightleftharpoons\limits_{{k}_{-1}}^{{k}_{1}}{{{\rm{EA}}}}\mathop{\to }\limits^{{k}_{2}}{{{\rm{E}}}}+{{{\rm{P}}}}$$

Historically, most publications on the reaction kinetics of enzymes record the key parameters *V*_max_ or *k*_cat_ and *k*_*m*_ or *k*_*d*_ that are associated to Michaelis-Menten kinetics, since they are relatively easy to measure. Additionally, they can be used for an analytical approximation of the trajectory of the system. For the analytical treatment, the mentioned parameters only apply in certain situations and are not generally valid if some properties of the system do not match the assumptions. The first restriction is that the enzyme concentration *c*(*E*) in the solution is much less than the substrate concentration *C*(*A*):

12 $$c(E)\ll c(A)$$

The corresponding reaction rate of the system can be calculated by

13 $$v=\frac{dc(P)}{dt}=\frac{{k}_{\rm{cat}}\cdot c(E)\cdot c(A)}{{k}_{d}+c(A)}$$

where *k*_cat_ has the properties of a first-order reaction rate and describes the capacity of the enzyme-substrate complex to produce product *P*. The Michaelis constant *k*_*m*_ describes the affinity of the enzyme and the substrate ${k}_{m}=\frac{{k}_{-1}+{k}_{2}}{{k}_{1}}$.

Further, it is assumed that the concentration of the intermediate complex does not change on the timescale of product formation, since all enzymes are bound to a substrate molecule (the result of Assumption (13)). This so-called quasi-steady-state approximation is therefore only valid if:

14 $$\frac{c{(E)}_{0}}{c{(A)}_{0}+{k}_{m}}\ll 1$$

where *c*(*E*)_0_ and *c*(*A*)_0_ are the initial enzyme and substrate concentrations. In situations where neither of the models is applicable, more complex modeling approaches are taken. In general, such reactions are split into smaller sub reactions, which are then solved with reversible reaction kinetics. The downside is that more parameters are required, which are often hard to resolve experimentally. In this work, Michaelis–Menten kinetics are only used if no other alternative could be determined. If applied, the assumptions under which Michaelis–Menten treatment is valid are discussed.

In general, the change in concentration resulting from reaction *r* ∈ *R* and chemical entity *e* ∈ *E* is determined by

15 $${c}_{i,j}^{n+1}={c}_{i,j}^{n}+{{\Delta }}t\mathop{\sum}\limits_{r\in R}f(r,e,{t}_{n},{c}_{i,j}^{n})$$

where $f(r,e,{t}_{n},{c}_{i,j}^{n})$ is the change in concentration resulting from reaction *r* regarding entity *e*, at time *t*_*n*_ and grid point ${c}_{i,j}^{n}$.

#### Numerical error handling

The numerical solution ${\tilde{c}}_{{{\Delta }}s}^{{{\Delta }}t}$ is an approximation of the actual solution *c*. In general, there are two ways to ensure sufficient accuracy of a numerical method:

- Reduce the step sizes Δ*t* and Δ*s* or,
- increase the methods’ convergence order *p*.

The numerical method is of convergence order *p*, if there is a number *H* independent of Δ*t*, such that

16 $$| {\tilde{c}}^{{{\Delta }}t}-c| \le H{{\Delta }}{t}^{p}$$

for small Δ*t*. The constant *H* depends on the actual solution of the problem. A decrease in the time step has a more drastic effect on the accuracy of the solution if *p* is large. If *H* is known, the order of the solution can be determined by evaluating the ratios of the errors between, $c-{\tilde{c}}^{{{\Delta }}t}$ and $c-{\tilde{c}}^{{{\Delta }}t/2}$

17 $${\log }_{2}\left|\frac{{\tilde{c}}^{{{\Delta }}t}-c}{{\tilde{c}}^{{{\Delta }}t/2}-c}\right|=p+{{{\mathcal{O}}}}({{\Delta }}t)$$

A similar approach is used to evaluate the accuracy of methods with unknown *H*. This approach is progressively comparing differences at Δ*t* and Δ*t*/2 with Δ*t*/2 and Δ*t*/4, which also leads to an estimation of the convergence order *p*.

To improve the convergence order, the midpoint method with embedded step width adjustment was implemented. Using an embedded method allows estimating the local truncation error of a single numerical step and provides leverage to control the local error with an adjustment of the step width.

The midpoint method is a simple Runge–Kutta method, a family of methods that increase the accuracy of the solutions by considering an estimated slope at subintervals of the current time step. In general, the consideration of more subintervals increases the convergence order, but results in more function evaluations per time step. The midpoint method is defined as follows:

18 $${c}_{i,j}^{n+1}={c}_{i,j}^{n}+f\left({t}_{n}+\frac{{{\Delta }}t}{2},{c}_{i,j}^{n}+\frac{{{\Delta }}t}{2}f({t}_{n},{c}_{i,j}^{n})\right).$$

The error of the first-order approximation ${{{\Delta }}}^{1}{c}_{i,j}^{{{\Delta }}t}=f({t}_{n},{c}_{i,j}^{n})$ (Euler’s method) can be estimated by comparing it to the second-order approximation ${{{\Delta }}}^{2}{c}_{i,j}^{{{\Delta }}t}=f({t}_{n}+{{\Delta }}t/2,{c}_{i,j}^{n}+{{\Delta }}t/2f({t}_{n},{c}_{i,j}^{n}))$. Since both approximations need to be calculated regardless, no additional function evaluation is necessary. The resulting adaptive Butcher tableau is:19

During simulation, the local truncation error ${e}_{local}^{n+1}$ can be calculated at every step:

20 $${e}_{\rm{local}}^{{{\Delta }}t}={{{\Delta }}}^{1}{c}_{i,j}^{{{\Delta }}t}-{{{\Delta }}}^{2}{c}_{i,j}^{{{\Delta }}t}$$

The concentration at each grid point (*i*, *j*) ∈ Ω is subject to change. This change can be the result of chemical reactions, transport, and diffusion processes. Furthermore, vesicles move across *L*_*x*_ × *L*_*y*_ and carry chemical entities at their surfaces. This changes the concentration of key molecules that are able to react in each grid point and time step, and even introduces reactions that would not occur otherwise.

Concentration-based modules encapsulate the concentration change that results from any process that creates and/or consumes chemical entities. Each module calculates ${{\Delta }}{c}_{i,j}^{{{\Delta }}t}$ for a chemical entity involved in the process. Furthermore, the local error is evaluated for each module individually. Since the changes span different timescales, the error is not evaluated absolutely as described in Equation (21) but relatively:

21 $${\varepsilon }_{\rm{local}}^{{{\Delta }}t}=lo{g}_{10}\left(\left|\frac{{{{\Delta }}}^{1}{c}_{i,j}^{{{\Delta }}t}}{{{{\Delta }}}^{2}{c}_{i,j}^{{{\Delta }}t}}\right|\right)$$

Increasing divergences between the solutions of ${{{\Delta }}}^{1}{c}_{i,j}^{{{\Delta }}t}$ and ${{{\Delta }}}^{2}{c}_{i,j}^{{{\Delta }}t}$ result in values larger than 0. A user-defined threshold confines the accuracy of the solutions. The local error will be calculated for every grid point (*i*, *j*) ∈ Ω and chemical entity involved in the process. After all errors are calculated, the largest local error is determined:

22 $${\varepsilon }_{\rm{local}}^{{{\Delta }}t}(m)=\mathop{\max }\limits_{e\in {E}_{m},(i,j)\in {{\Omega }}}{\varepsilon }_{\rm{local}}^{{{\Delta }}t}(e,i,j)$$

The error of the module *m* ∈ *M* is the maximal error for each grid point (*i*, *j*) ∈ Ω and entity *e* ∈ *E*_*m*_ referenced in the module. A local numerical error is acceptable, if it is smaller than a user defined threshold *τ*_*local*_. Hence, the time step is decreased, if $\exists m({\varepsilon }_{\rm{local}}^{{{\Delta }}t}(m) \,>\, {\tau }_{\rm{local}})$. Further, a module is defined as *critical*, if ${\varepsilon }_{\rm{local}}^{{{\Delta }}t}(m) \,>\, {\tau }_{\rm{local}}\cdot {\theta }_{\rm{local}}$, where *θ*_local_ < 1 is the local tolerance. Whenever $\forall m({\varepsilon }_{\rm{local}}^{{{\Delta }}t}(m)\,<\, {\tau }_{\rm{local}}\cdot {\theta }_{\rm{local}})$ the time step is increased to decrease computation time. A maximal local error of 5% can be achieved by setting the threshold to *τ*_local_ = *log*(0.05). A tolerance *θ*_local_ = 0.5 would result in an increased time step, if the computed local error ${\varepsilon }_{\rm{local}}^{{{\Delta }}t}(m)$ is smaller than 2.5%.

In addition to the local ${\varepsilon }_{\rm{local}}^{{{\Delta }}t}(m)$ error, the total error ${\varepsilon }_{\rm{total}}^{{{\Delta }}t}(m)$ is evaluated. The total error is determined by transferring the previous idea of error calculation. Instead of determining the error based on the module, the total influence of all modules on the concentration of an entity is evaluated. Therefore, the local error is optimized first and the total change in concentration is calculated per entity and grid point.

23 $${{{\Delta }}}_{\rm{total}}{c}_{i,j}^{{{\Delta }}t}(e)=\mathop{\sum}\limits_{m\in M:e\subseteq {E}_{m}}{{\Delta }}{c}_{i,j}^{{{\Delta }}t}(e)$$

Using the reasoning from the midpoint method, a scaffold concentration is calculated that can be used to determine the influence of the time step

24 $${c}_{i,j}^{n+\frac{1}{2}}(e)={c}_{i,j}^{n}(e)+\frac{1}{2}{{{\Delta }}}_{\rm{total}}{c}_{i,j}^{{{\Delta }}t}(e)$$

Henceforth, ${c}_{i,j}^{n+\frac{1}{2}}(e)$ is used to calculate ${c}_{i,j}^{n+1}(e)$ by determining ${{{\Delta }}}_{\rm{total}}{c}_{i,j}^{{{\Delta }}t}(e)$ at ${t}_{n+\frac{1}{2}}$. The resulting total truncation error is

25 $${e}_{\rm{total}}^{{{\Delta }}t}={{{\Delta }}}^{n+1}{c}_{i,j}^{{{\Delta }}t}-{{{\Delta }}}^{n+\frac{1}{2}}{c}_{i,j}^{{{\Delta }}t}$$

The total error is calculated using the same approach as chosen for the local error:

26 $${\varepsilon }_{\rm{total}}^{{{\Delta }}t}=lo{g}_{10}\left(\left|\frac{{c}_{i,j}^{n+1}}{{c}_{i,j}^{n+\frac{1}{2}}}\right|\right)$$

A total numerical error is acceptable, if it is smaller than a user defined threshold *τ*_*total*_. Hence, the time step is decreased, if $\exists m\in M:{\varepsilon }_{\rm{total}}^{{{\Delta }}t}(e) \,>\, {\tau }_{\rm{total}}$. Further, the current time step is defined as critical, if ${\varepsilon }_{\rm{total}}^{{{\Delta }}t}(e) \,>\, {\tau }_{\rm{total}}\cdot {\theta }_{\rm{total}}$, where *θ*_total_ < 1 is the local tolerance. Whenever $\forall m\in M:{\varepsilon }_{\rm{total}}^{{{\Delta }}t}(e) \,<\, {\tau }_{\rm{total}}\cdot {\theta }_{\rm{total}}$ the time step is increased to decrease computation time. This procedure is time-consuming, since four function evaluations are required per time step. Two for the initial computation of the local error and two more for the computation of the total error. In practice, the total error is primarily required at the beginning of the simulations, when there are large differences in concentration that are subject to diffusion or fast reactions. Two approaches were designed to reduce the number of calculations required.

The first approach considers the type of parallelization and optimization of local errors. The computation is parallelized on module level. In each time step, each module computes its local updates and errors individually. If any module encounters, ${\varepsilon }_{\rm{local}}^{{{\Delta }}t}(m) \,>\, {\tau }_{\rm{local}}$ the computation is interrupted. The interrupting module keeps requesting decreases in the time step. With each request, the new ${\varepsilon }_{\rm{local}}^{{{\Delta }}t}(m)$ is calculated, but only for the grid point (*i*, *j*) where the error originally occurred, until ${\varepsilon }_{\rm{local}}^{{{\Delta }}t}(m) \,<\, {\tau }_{\rm{local}}$. The non-interrupting modules clean their previously calculated deltas. After Δ*t* was determined, all modules run again with the decreased time step. Again, if any module is above the error threshold, it is optimized individually. This procedure, prevents unnecessary optimization of non-critical modules and additionally saves time by only optimizing the most critical grid point at a time. This approach is valid if two conditions are satisfied. First, a decrease in time step leads to an increase in accuracy. If the most inaccurate module is optimized, accuracy of the other modules improves as well. Second, the optimization of the most critical region leads to a proportional accuracy gain in less critical regions. If the most critical region is below the error threshold, less critical regions would be as well. To keep both assumptions, the functions implemented by each module need to be continuous and differentiable.

A second concept implemented to decrease calculations per time step is total error skipping. The total error is said to be negligible, if it is smaller than the negligibility threshold *ν*: ${\varepsilon }_{\rm{total}}^{{{\Delta }}t}(e) < \nu \ll {\tau }_{\rm{total}}$, whereby *ν* is small in comparison to *τ*_total_. If this is the case, calculation of the global error will not be performed until the following criteria are met:

- The time step was increased as a result of step width optimization, or
- A number of time steps have been performed since the last total error was calculated.

The calculation of the local error is performed at any time step, regardless of the global error. If the time step decreases during local error calculation, the already small global error would decrease even more and computation is not necessary. Alternatively, if the local error calculation allows for an increased time step, the global error needs to be considered again. As a backup, the total error is additionally computed after a fixed amount of time steps without evaluation. Empirically, it was determined that the global error is primarily relevant for the initial time steps. Concentration changes are rapid in the beginning of the simulation, depending on the initialization of the system. After some equilibrating time, the global error decreases and does not affect the time step. Nevertheless, the negligibility threshold should be chosen with care. In practice, a default value of *ν* ≈ *τ*_total_/10,000 provides a good trade-off between computation time and secured accuracy.

#### Rule-based reaction creation

In complex biochemical systems, the enumeration of all reactions that might occur can be cumbersome at least or impossible at worst. Especially, proteins with multiple post-translational modification sites are hard to model with classical approaches, since a protein with *n* modification sites can exist in 2^*n*^ different states. The interaction of protein complexes with modifications further aggravates the number of possibilities and quickly leads to thousands of chemical entities and reactions^113^. Rule-based modeling allows for the specification of so-called reactive motifs to avoid the enumeration of all possible reactions^114^. Several approaches exist that allow the creation of reaction networks using reaction rules to condense the number of specifications that have to be made to describe the system. A comprehensive collection of tools has been compiled elsewhere^115^. The most important tool from a cell signaling perspective is BioNetGen^114^. The BioNetGen approach allows for the specification of chemical entities, kinetics, and rules in a domain-specific notation, as well as the subsequent simulation of the generated reaction network. Graphs rewriting is used to track connections and states of the involved molecules, with the goal of creating a reaction network with all the required reactions and components^116^. The work of Faeder and colleagues was used as a basis and inspiration for a modified approach. Mainly, two considerations drove the choice for an adaptation.

We wanted to explicitly distinguish between transport and reaction phenomena to improve the modularization of a system. BioNetGen uses states in reaction rules to represent transport between different compartments in a system. The definition of compartments in an explicitly spatial system we used in this study is not captured by the state-based approach. We define reactions strictly as the structural transformation of one entity to another. Transport on the other hand only considers the movement of structurally identical chemical entities and is performed by dedicated modules. The compartmentalization is done in another step of the modeling process, and therefore untangling both levels results in a higher degree of reusability and encourages the design of faithful models. Furthermore, the reaction network becomes less complex since additional compartments do not result in additional rules and generated reactions. This approach requires a more mechanistic approach to modeling, which can be troublesome for poorly understood systems, but also provides the possibility to increase understanding by elevating some complexity.

Another consideration is the definition of binding sites and molecule states. Molecules very rarely have different states, if they have the same structure. Therefore, in our approach, states are also represented as the actual addition or removal of a chemical entity (for example a phosphorylation reaction adds phosphate to a chemical entity). Again, this reduces modeling ambiguity and encourages mechanistic approaches. This leads to more complex graph structures, but removes states and therefore reduces the complexity of the actual representation. Furthermore, it provides flexibility when designing binding sites. During reaction definition, it is possible to specify a binding site, or let the network generation automatically assign a binding site.

In conclusion, the modified rule-based definition of reactions abolishes states of chemical entities, and provides a more flexible way to define and modify systems of rules. Although it is possible to gain computational efficiency by removing states, it comes at the cost of more complex chemical entities that need to be considered.

##### Reaction definition

Reaction rules are combinations of basic reactors in so-called reaction chains. The basic reactors are:

- **ADD**, add a component *B* to another component *A*, resulting in one component *A*-*B*. Only *A* needs to be available as a reactant.
- **BIND**, adds a component *A* to another component *B*, resulting in one component *A*-*B*. Both *A* and *B* need to be available as reactants.
- **REMOVE**, removes a component *B* from another component *A*−*B*, resulting in component *A*. *B* is not a product of the reaction.
- **RELEASE**, splits component *A*−*B* to *A* and *B*. Both *A* and *B* are products of the reaction.

While **ADD**, **BIND**, and **REMOVE** can be concatenated freely, **RELEASE** is always a terminating basic reaction, since there are two resulting products. Before the application of a reactor, a set of implicit and explicit filters is applied to the candidate molecules to determine which candidates participate in the reaction (for a detailed description, see Supplementary Table 16). Additionally, explicit conditions describe hypotheses for the requirements of each reaction to occur. For example, in order to phosphorylate AQP2, PKAC needs to be bound to AQP2. To describe reaction chains, a step-wise builder pattern was employed and implemented. Step-wise builders are a design pattern that allows to shift of some expression validation from run time to compile time. Effectively, the step-wise builder describes a formal grammar for the generation of reactions. The grammar restricts the number of possible combinations of words, provides feedback upon definition, and with modern integrated development environments even guides the process of creation.

##### Entity and network generation

All reaction chains are used to generate a reaction network. The fundamental entity of any reaction is the simple chemical entity (SE). Simple entities *v* ∈ *V* can be combined with complex chemical entities *G*. This is done using a graph representation, where the nodes *v* ∈ *V* are SE that are connected by edges. The connection is only possible if a binding site *S* = (*v*_1_, *v*_2_) is specified. A binding site is a pair of names SE. Only if a binding site (*v*_1_, *v*_2_) exists, an edge can be created between two nodes *v*_1_ and *v*_2_ in the complex entity. The binding site can be referenced by its name in reaction definitions. A SE is considered either a small molecule or a multi-site molecule. Small molecules are considered to have one binding site and can be only bound to one molecule at a time (e.g., ATP). Additionally, a SE can be specified to be membrane-bound. If a reaction involves any membrane-bound entity, the resulting complex entity will be also considered membrane-bound, if not explicitly specified otherwise.

**Fig. 9:**
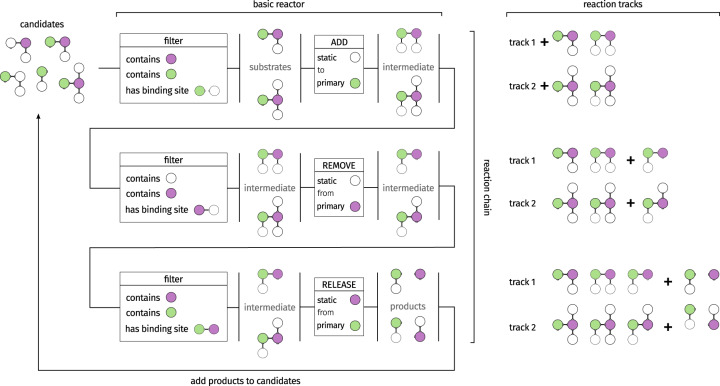
Reaction network generation. Reactions can be specified by a description of the reaction process. Basic reactors are concatenated to chains that are processed during the reaction network generation. Additionally, conditions allow for further specification of the reaction process.

The description of interactions and their modification is described by reaction rules. During network generation, a reaction chain takes from a set of candidate reactants, applies the next basic reaction in a reaction chain, and creates an intermediate product. The elementary “reactors" use the intermediate products and apply additional filter criteria to further specify a reaction. To generate the full reaction, the substrates and intermediate products are gathered in a set of stack data structures, called tracks. A track represents the transformations necessary to produce a specific product. The entities that are on top of the stack are the final products of the reactant, and the entities at the bottom are the original substrates. The products of each reaction are added to the pool of candidates for the next application of the network generation (see Fig. 9 and Supplementary Algorithm 1).

### Definition of macroscopic components

The simulation space represents a pseudo-three-dimensional slice of a biological system with arbitrary width and length, but a fixed height. Initially, the simulation space is compartmentalized into a rectangular mesh to prepare for the subsequent application of finite-difference methods. Hereby, the total area of simulation is segmented into a predefined amount of rows and columns. The tiling is further subdivided to compartments by membranes (see Fig. 7). A membrane can be placed at the border between two grid points. A single compartment is assigned to each grid point that is not bordered by a membrane, whereas two compartments are assigned to grid points adjacent to a membrane. As a consequence, one compartment represents the concentration of entities in the membrane, whereas the other represents the concentration in the remaining space. Each compartment contains a set of chemical entities that are assigned a floating-point number based on their concentration in this space. Each compartment is considered well mixed. The space can be set up using raster images (e.g., PNG files), where each pixel represents a grid point and colors represent different compartments. Areas of the same color are automatically surrounded by a membrane.

Macroscopic entities are modeled as agents that are able to interact with each other and with the reaction spaces (see Fig. 7). The interactions are defined by a set of conditions that have to be met before associated actions are performed. We classified macroscopic entities in the cell into four types: sphere-like, line-like, surface-like, and volume-like based on their relevant dimensionality. We reason that small membrane-enclosed entities such as transport vesicles, lysosomes, and peroxisomes can be categorized by their approximately spherical shape and their ability to move actively or passively through the cell. Membrane-enclosed compartments that are non-spherical and basically stationary due to their size can be represented by area-like agents. Area-like agents are connected two-dimensional regions that delineate compartments, such as the nuclear envelope or the cell membrane. Line-like agents are approximately two-dimensional line segments that represent the different kinds of cytoskeletal filaments. Line-like agents have the ability to grow or shrink and act as scaffold for the active transport of sphere-like agents. Volume-like agents are sections of the cell that are able to represent different environmental conditions. Diffusive reduction in certain areas of the cell was implemented using volume-like agents, but other applications such as different pH values or temperatures are thinkable. Those abstract agent types act as blueprints that are implemented with specific parameters and behaviors based on the application. A vesicle possess a state, a position, a radius, and two compartments. The compartments represent the internal cargo and the membrane surface. The state of a vesicle determines how it is processed by modules. Since vesicles move, they have to change their neighboring compartments dynamically during simulation. This entails that each vesicle is referenced to up to four additional compartments. If a vesicle is on the border between a number of grid cells, the area of the intruding membrane section is calculated and used to scale the exposed concentrations of chemical entities. Apical and perinuclear membranes are implementations of area-like agents, made up of membrane segments. Each segment is represented by an area between two membranes separated grid points. This allows for the calculation of the membrane area per compartment and is for example used to scale the number of reactants available to reactions. Microtubules and actin filaments are line-like agents for the directed transport of vesicles. They are composed of multiple segments and possess a positive and a negative end to indicate a direction. This directionality is used for the transport of vesicles that are attached via molecular motors.

The introduction of macroscopic components requires further compartmentalization of the system to account for chemical entities carried by vesicles or associated with membranes. The subdivision achieved by the numerical treatment of the simulation space during diffusion discretion is reused and further refined. Topological descriptors are introduced, such that each agent has a set of concentrations $c(a,u,s,t)\in {\mathbb{R}}$, if the agent is associated with at least one topological descriptor. The concentration is uniquely identified by the combination of agent *a* ∈ *A*, topological descriptor *u* ∈ *U*, and entity *e* ∈ *E*. Each combination of topological descriptor and agent, defines a new compartment, where reactions are able to apply changes in concentration.

#### Sphere-like agents

are defined by a position *p*, a radius *r*, a state *s* ∈ *S* and two topological descriptors for membrane and cargo. For the context of this work, sphere-like agents have been implemented for vesicles and endocytotic pits.

#### Line-like agents

are defined by a set of positions with *P* = {(*x*_1_, *y*_1_), (*x*_2_, *y*_2_), …, (*x*_*n*_, *y*_*n*_)}, with two consecutive positions (*x*_*i*_, *y*_*i*_), (*x*_*i*+1_, *y*_*i*+1_) representing a line segment. Line-like agents have no topological descriptors and therefore also no compartment with associated chemical entities. Line-like agents are used as guides for displacement-based modules that move vesicles along cytoskeleton filaments. Filaments are initialized using a 2D adaptation of the microtubule growth algorithm as presented by^25^.

#### Surface-like agents

are defined by a set of positions with *P* = {(*x*_1_, *y*_1_), (*x*_2_, *y*_2_), …, (*x*_*n*_, *y*_*n*_)}, with two consecutive positions (*x*_*i*_, *y*_*i*_), (*x*_*i*+1_, *y*_*i*+1_) representing a surface segment. The area of a surface segment can be determined by multiplying the length of the segment (*x*_*i*_, *y*_*i*_), (*x*_*i*+1_, *y*_*i*+1_) with the spatial step width Δ*s*. The spatial representation *r* of a grid point (*x*, *y*) can be represented by a square with vertices (*x* − Δ*s*/2, *y* − Δ*s*/2), (*x* − Δ*s*/2, *y* + Δ*s*/2), (*x* + Δ*s*/2, *y* + Δ*s*/2), (*x* + Δ*s*/2, *y* − Δ*s*/2). Surface segments must be defined at the interface of two neighboring grid points, and therefore along the edges of the spatial representations of grid points. It follows that the set of positions *P* is constrained by the vertices that arise from all grid points. Surface-like agents have one topological descriptor that defines a compartment. Membranes are implementations of surface-like agents.

#### Volume-like agents

Volume-like agents are defined by a set of points *P* = {(*x*_1_, *y*_1_), …, (*x*_*n*_, *y*_*n*_)}. The points define the vertices of a polygon, and two consecutive positions (*x*_*i*_, *y*_*i*_), (*x*_*i*+1_, *y*_*i*+1_) represent a volume border segment. Additionally, the starting point (*x*_1_, *y*_1_) and the last point (*x*_*n*_, *y*_*n*_) are always connected. Volume-like agents specify areas of the simulation system where a distinct set of modules or features should be applied. For example, the cell cortex can be defined as a volume-like agent close to the cell membrane. Modules are used to recognize vesicular movement into the volume, and a specific action for the entering vesicle can be assigned. Volume-like agents define no compartments.

#### Agent behavior

Displacement-based modules are used to change the position of sphere-like agents. A module $m\in {\mathbb{M}}$ is a function applied to an agent *a* ∈ *A* and results in a change in position *m*(*a*, Δ*t*) = Δ*p*(*a*, Δ*t*) scaled to the current time step width Δ*t*. The state and/or the concentration of chemical entities in the compartment associated with the agent *a* can be used to determine the displacement. The next position ${p}_{a}^{n+1}=p(a,{t}_{n+1})$ of the agent *a* ∈ *A* is determined by summation of all displacements, Δ*p*(*a*, Δ*t*) denoted as ${{\Delta }}{p}_{a}^{{{\Delta }}t}$.

27 $${p}_{a}^{n+1}={p}_{a}^{n}+\mathop{\sum}\limits_{m\in {\mathbb{M}}:\parallel m(a,{{\Delta }}t)\parallel > 0}{{\Delta }}{p}_{a}^{{{\Delta }}t}$$

∥ ⋅ ∥ denotes the euclidean norm. The displacement must not be too large, otherwise the simulation could experience numerical instabilities whenever concentrations change rapidly. A similar concept to the numerical error is used to determine if the displacement is appropriate. The total displacement is determined by:

28 $${{{\Delta }}}_{\rm{total}}{p}_{a}^{{{\Delta }}t}=\mathop{\sum}\limits_{m\in {\mathbb{M}}:\parallel m(a,{{\Delta }}t)\parallel > 0}{{\Delta }}{p}_{a}^{{{\Delta }}t}$$

should also not be too small to ensure efficient computation. Hence, a reference distance *d*_ref_ is used to evaluate the displacement. Usually, this reference distance is given as a fraction of the spatial step width Δ*s*.

The deviation of the displacement is calculated by:

29 $$D(a)=lo{g}_{10}\left(\frac{\parallel {{{\Delta }}}_{\rm{total}}{p}_{a}^{{{\Delta }}t}\parallel }{{d}_{ref}}\right)$$

and compared to two thresholds ${\theta }_{\rm{disp}}^{+}$ and ${\theta }_{\rm{disp}}^{-}$. The time step can be decreased, if, $\exists a\in A:D(a) > {\theta }_{\rm{disp}}^{+}$ and increased if $\forall a\in A:D(a) < {\theta }_{\rm{disp}}^{-}$.

The final position *p*(*t* + 1) is only applied if the target position is not already occupied by other agents, and if no membranes need to be crossed to arrive at that position. Three different methods can be used to determine the updated position if any collision should happen: a ballistic reflection, a recalculation (if there is a randomized component to any update, the calculation can be repeated until a valid position is found), or simply discarding the update by setting *p*_*t*+1_ = *p*_*t*_. It was found that the different methods produce only marginally different outcomes^117^ in sparse setups. For this work, the last method was chosen, and collision detection has been implemented as described in Supplementary Algorithm 2. To increase the efficiency of the collision detection algorithm, only local interaction partners are considered. This can be achieved using a spatial indexing approach. A spatial grid has already been created using the numerical grid. This indexing will also be used to reference vesicles to the respective regions in the simulation system and vice versa. Supplementary Algorithm 3 performs indexing and additionally defines, which amount of surface area is associated with each grid point. The association is repeated in each time step, after the displacements have been calculated. The procedure is only valid, if the radius *r* of any vesicle *a* is smaller than the spatial step width Δ*s*.

##### Vesicle diffusion

The diffusion of vesicles is modeled as described by Klann^24^. The position of the vesicle changes scaled by its diffusivity:

30 $${{\Delta }}{p}_{a}^{{{\Delta }}t}=\sqrt{2{D}_{v}}\cdot \overrightarrow{\xi }$$

where the change in position Δ*p*(Δ*t*) is calculated by a random Gaussian vector *ξ* with mean 0 and variance 1. The diffusion coefficient was determined from literature^95^. A volume-like agent can be used to add collision boundaries to the collision detection algorithm and therefore hinder the diffusion of the vesicle.

##### Filament-guided transport

Directed vesicle transport happens at line-like agents. The displacement delta is calculated by:

31 $${{\Delta }}p({{\Delta }}t)={v}_{m}\cdot \hat{u}$$

where *v*_*m*_ is the average velocity of the attached molecular motor and $\hat{u}$ is a unit vector that points in the direction specified by the pulling motor, along the cytoskeleton filament. Whether the vesicle is attached to a filament is determined by a qualitative state-changing module. This module sets the state of the vesicle and the line segment (*x*_*i*_, *y*_*i*_), (*x*_*i*+1_, *y*_*i*+1_) it will be attached to. The calculation of the movement is described in Algorithm 6.

The transport speed scaled by a number of specified molecules present in the coat of the vesicle. The actin boost vesicles experience after they have been scissioned by clathrin-mediated endocytosis^118^ was implemented using directed, unguided transport. In this implementation, the unit vector $\hat{u}$ points orthogonal to the membrane segment the vesicle spawned from. The velocity *v*_*m*_ is calculated by *v*_*m*_ = *v*_*b*_ ⋅ *c*_*s*_, where *s* is the scaling entity (clathrin) and *v*_*b*_ is the base velocity given in [space]/[time] ⋅ [concentration].

##### State-changing modules

Vesicles can change their state in a variety of cases:

1. position is close to an area-like agent,
2. position is close to a line-like agent,
3. position is inside a volume-like agent,
4. concentration of a chemical entity reaches a threshold, and
5. by chance.

These conditions have been implemented as qualitative modules. State-changing modules are initialized with a list of states where the module is applied and the test criteria that specifies the condition that should be met. In cases 1 and 2, the closest segment of the specified agent is determined and the distance between the vesicle membrane and the closest point of the segments is evaluated. In case 3 the centroid of the vesicle is evaluated using the even-odd rule algorithm^119^. In case 4 the concentration currently in the cargo or membrane compartment of the vesicle is evaluated. A state change by chance is also interesting, for example when a vesicle is transported along a cytoskeletal filament, the vesicle can spontaneously detach from the filament. The possibility of a state change happening is given as a frequency and is scaled with the time step. If the probability that the event happens in a single time step is larger than 1 the time step is reduced and recalculated.

#### Endocytosis

The algorithm implemented for endocytosis can be reviewed in Supplementary Algorithm 4. The module manages the creation of clathrin-coated pits, an implementation of sphere-like agents. Furthermore, it manages the creation of clathrin-coated vesicles, after the pit passes a concentration checkpoint and maturation. Initially, a pit formation rate *k*_*p*_ pit formation rate in 1/[time] ⋅ [space]^2^ determines how often clathin-coated pits are spawned. In each successful time step, cargo molecules are added to the pit with a cargo addition rate *k*_*a*_ from associated membranes using a concentration-based module.

The cargo absorption module is a specialized reaction. The addition of cargo to the pit is scaled by inhibiting and catalyzing chemical entities. The actual cargo addition rate is calculated by:

32 $${k}_{c}=\frac{{c}_{{{\mbox{cat}}}}}{{c}_{{{\mbox{cat}}}}+{c}_{{{\mbox{inh}}}}}\cdot {k}_{b},$$

where *c*_cat_ is the concentration of the catalyzing entity, *c*_inh_ is the concentration of the inhibiting entity and *k*_*b*_ base cargo addition rate. It follows that the maximal applicable rate $\max ({k}_{c})={k}_{b}$ and decreasing amounts of the catalytic entity or increasing amounts of the inhibiting entity scale the applicable rate linearly. The amount of cargo that is moved from the membrane-associated with the pit is calculated by ${{\Delta }}{c}_{\,{{\mbox{cargo}}}}^{n+1}={k}_{c}\cdot {c}_{{{\mbox{cargo}}}\,}^{n}$.

The assembling pit exists until a maximal time *t*_*cp*_ that is determined upon vesicle creation. If the pit does not reach the required concentration *c*_*cp*_ of specified cargo entities *E*_*c*_ until this time, the pit is aborted and the concentrations in the pit are moved back to the associated membrane. Otherwise, the pit reaches the required cargo concentration and enters a maturation phase that ends in time *t*_*m*_ with the creation of a new vesicle, the cargo molecules, and a set of predefined additional cargo molecules including the clathrin coat.

#### Fusion

The algorithm implemented for fusion can be reviewed in Supplementary Algorithm 5. Parameters that influence this module are the fusion time *t*_*f*_, attachment distance *d*_*a*_, two chemical entities, one for the Q-SNARE *S*_*Q*_ and one for the R-SNARE *S*_*R*_, as well as the minimal number of snare pairs *n*_*p*_. The fusion time *t*_*f*_ is used to determine the total length of the fusion process until the vesicle fully fuses and transfers its contents. The attachment distance is the minimal distance between the vesicle and membrane that is required to initiate the fusion process. If a vesicle is close enough to any membrane, it is checked if *n*_*p*_ fusion pairs can be formed from the Q-SNARES in the target membrane and R-SNARES in the vesicle membrane.

## Supplementary information

Supplementary Information

## Data Availability

Simulation parameters and setup details are available in Supplementary Information 1. Individual simulation trajectories generated and analyzed during the current study are available from the corresponding author on reasonable request.

## Code availability

Source code of the simulation and modeling framework SiNGA developed for this work is available at: https://github.com/singa-bio/singa. Version 0.7.0 was used to simulate models presented in this work^120^.
