# Supplementary material for: A multiscale model of the regulation of aquaporin 2 recycling
Source: NPJ Syst Biol Appl. 2022 May 9;8:16. doi: 10.1038/s41540-022-00223-y (PMC9085758; doi:10.1038/s41540-022-00223-y)
Supplement: Supplementary file 1 — Supplementary Information [file 41540_2022_223_MOESM1_ESM.pdf]

Supplementary Information

Christoph Leberecht[a,b,*], Michael Schroeder[a], and Dirk Labudde[b]

[a]Biotechnology Center (BIOTEC), TU Dresden, Dresden, 01307, Germany
[b]University of Applied Sciences Mittweida, Mittweida, 09648, Germany
[*]christoph.leberecht@tu-dresden.de

SUPPLEMENTARY INFORMATION

The supplementary information document contains detailed model descriptions including background, details, parameters, and corresponding literature. Additionally, an overview of the modules implemented in this paper, and pseudocode of algorithms used in agent based modules is included.

1 Model Descriptions

Modeling was performed based on literature study. We analyzed cellular processes and their connection in renal principal cells. It was not our goal to reproduce progressions of concentration trajectories exactly. Rather, we qualitatively evaluated the behavior of the system and determined whether it behaves as stated in previous studies. We created models that include the entities that have been proven significant for the system and evaluated their role in it. Furthermore, we took care to keep reactions as elementary as possible and strictly separate transport phenomena. This approach results in models that can be incrementally refined to include other cellular components, while simultaneously reducing the requirements to manually modify existing parts.

Each model is prefaced by a paragraph that explains the biological background that lead to the design decisions, followed by details of the simulation conditions. All modules that were used in the model are listed in the modules section. The type column references the module implementation from the SiNGA framework with a short description. A single module may consist of multiple reactions that have been generated by the same reaction rule. Features, referenced in the subsequent feature table, are parameters that determine the concrete behavior of the module (such as reaction rates). Evidence for modules indicate literature sources where the process itself was described, or applicable reaction equations are given. Chemical entities are represented as graph structures and for the sake of textual display node have been printed in alphabetical order separated by hyphens. Parameters are listed in a similar tabular style. Literature is referenced for each feature. Additionally, the simulated variations are given in the last table of each model subsection.

1.1 PKA activation and AQP2 phosphorylation

Phosphorylation of S256 triggers vesicle departure AQP2 is stored in vesicles close to the nucleus of the cell[1–3] in the basal state of principal cells. AQP2 has multiple phosphorylation sites[4–6], out of which Serine 265 seems to play the most significant role for the exocytosis of the vesicles to the apical membrane[4,7,8]. How exactly S256 increases the frequency of this transport is currently not fully understood[9]. A study suggests that proteins of the myosin family 5 are activated directly by secretory vesicle cargo[10]. Other work indicates that Ca^{2+} oscillations, which are also triggered upon vasopressin recognition, were essential for AQP2 exocytosis[11,12]. Additionally, the ratio between phosphorylated and unphosphorylated AQP2 seems to play a significant role[13]. It was proposed that the phosphorylation of three monomers in a tetrameter is essential for its positioning in the apical membrane[13].

PKA is regulated by allostery The phosphorylation of S256 is introduced by protein kinase A catalytic subunit (PKAC)[7], which in its holoenzyme form is tightly bound to the protein kinase A regulatory subunit II (PKAR)[14]. The regulatory subunit is associated to the A-kinase anchoring protein 18δ (AKAP)[15], which is bound to the AQP2 vesicle membrane[16]. Additionally, AKAP provides binding sites for phosphodiesterase type 4D3 (PDE)[17] and Serine/threonine-protein phosphatase 2B (PP2B)[18]. The actual mechanism of PKA activation and regulation has been subject to lots of research and scientific debate[19]. The current consensus that was modeled is shown in the main manuscript. The regulatory subunit PKAR binds to the complex of PKAC and adenosine triphosphate (ATP)[20] which catalyzes autophosphorylation. PKAR is further able to bind two cyclic adenosine monophosphate (cAMP) molecules, whose binding sites have about the same association rate[21], but so-called site A has a higher dissociation rate ($6.3 \cdot 10^{-2}$ s^{-1}) in comparison to site B ($2.6 \cdot 10^{-6}$ s^{-1})[22]. Additionally, site A is only accessible after site B is occupied[22,23]. Strikingly, the complex of phosphorylated PKAR and PKAC-ADP only dissociates whenever both cAMP binding sites are occupied[23]. Consequently, in the basal state, the overwhelming majority of PKAC is bound to

it's already phosphorylated regulatory subunit[23]. Upon exposure to sufficiently large concentrations of cAMP the catalytic subunit is released and able to phosphorylate further targets. Walker-Gray et al. also found that PKAC stays close to the membrane via myristylation[24], which confines the area of action for PKAC and keeps the response to cAMP very localized. The phosphorylation slows reassociation of PKAC to PKAR even if no cAMP is bound[20]. Dephosphorylation is performed by PP2B[25], the phosphatase associated with AKAP. The concentration of the regulatory subunit of PKA was found to be up to 17-fold higher than the catalytic subunit[24]. This ensures efficient restraining of PKA subunits close to their origin of release.

Simplifications and estimations We have chosen to include the phosphorylation site S256 as the only phosphorylation site of AQP2. The consensus regarding this phosphorylation is currently the largest among all the phosphorylation sites of AQP2[4,8,26,27]. We refer to AQP2 phosphorylated at Serine 256 as AQP2-P. If the ratio of AQP2-P to total AQP2 surpasses 3/4, the vesicle is able to attach to cytoskeletal filaments[13]. The initial concentration of AQP2 was estimated as described in Supplementary Table 15. In short, we used the permeability and area of the cell membrane to determine the number of AQP2 in the cell membrane of resting and activated principal cells. We conclude that about 4800 AQP2 monomers would be present in inactive and, 11400 monomers would be present in active 1.6 μm^2 apical membrane. Furthermore, this requires about 10 vesicles with 650 monomers each[28] to fuse with the apical membrane to reach the measured permeability. The basal AQP2-P to AQP2 ratio was determined to be around 0.46 and was used to calculate the initial amount of AQP2 in vesicles[13].

Since the binding of cAMP at site A exposes the phosphorylation and PKAC binding site[20], we assume that PP2B preferably binds[18] to this open variant. We omitted the explicit modeling of ADP as a product of phosphorylation, as well as phosphate as a product of dephosphorylation, since they are only end products in this model. Furthermore, ATP was not modeled since it is not considered a rate limiting substrate[20,29]. All the components of the AKAP signaling complex are considered membrane bound[16–18,30]. The degradation of cAMP by PDE was modeled explicitly, since its phosphorylation by PKA[31] and its localization at vesicles[17] is critical for this pathway.

Supplementary Table 1. Phosphorylation model modules.

ID	Type	Features	Evidence
M001	Reaction	F01, F02	[32],[20]
	PKA activation: PKAR PKAC binding and phosphorylation		
	PKAC + AKAP−CAMP−PKAR ⇌ AKAP−CAMP−P−PKAC−PKAR		
	PKAC + AKAP−PKAR ⇌ AKAP−P−PKAC−PKAR		
	PKAC + AKAP−CAMP−CAMP−PKAR ⇌ AKAP−CAMP−CAMP−P−PKAC−PKAR		
M002	Reaction	F03	[33],[20]
	PKA activation: PKAC PKAR release		
	AKAP−CAMP−CAMP−P−PKAC−PKAR ⟶ PKAC + AKAP−CAMP−CAMP−P−PKAR		
M003	Reaction	F04, F05	[23]
	PKA activation: PKAR-P PKAC binding		
	PKAC + AKAP−CAMP−P−PKAR ⇌ AKAP−CAMP−P−PKAC−PKAR		
	PKAC + AKAP−CAMP−CAMP−P−PKAR ⇌ AKAP−CAMP−CAMP−P−PKAC−PKAR		
	PKAC + AKAP−P−PKAR ⇌ AKAP−P−PKAC−PKAR		
M004	Reaction	F06, F07	[23],[21],[22]
	PKA activation: PKAR CAMP pocket B binding		
	CAMP + AKAP−PKAR ⇌ AKAP−CAMP−PKAR		
	CAMP + AKAP−P−PKAR ⇌ AKAP−CAMP−P−PKAR		
	CAMP + AKAP−P−PKAC−PKAR ⇌ AKAP−CAMP−P−PKAC−PKAR		
M005	Reaction	F06, F08	[23],[21],[22]
	PKA activation: PKAR CAMP pocket A binding and PKAC release		
	CAMP + AKAP−CAMP−P−PKAC−PKAR ⇌ PKAC + AKAP−CAMP−CAMP−P−PKAR		
M006	Reaction	F06, F08	[23],[21],[22]
	PKA activation: PKAR CAMP pocket A binding		
	CAMP + AKAP−CAMP−P−PKAR ⇌ AKAP−CAMP−CAMP−P−PKAR		
	CAMP + AKAP−CAMP−PKAR ⇌ AKAP−CAMP−CAMP−PKAR		

Supplementary Table 1. Phosphorylation model modules.

ID	Type	Features	Evidence
M007	Reaction	F09, F10	[34,27,35,36]
	PKA phosphorylation: PKAC AQP2 binding		
	$\mathrm{AQP2 + PKAC \rightleftharpoons AQP2-PKAC}$		
M008	Reaction	F03	[27,35,36]
	PKA phosphorylation: PKAC AQP2 phosphorylation and release		
	$\mathrm{AQP2-PKAC \longrightarrow AQP2-P + PKAC}$		
M009	Reaction	F11, F10	[34,37,38]
	PKA phosphorylation: PKAC PDE4 binding		
	$\mathrm{PDE + PKAC \rightleftharpoons PDE-PKAC}$		
M010	Reaction	F03	[34,37,38]
	PKA phosphorylation: PKAC PDE4 phosphorylation and release		
	$\mathrm{PDE-PKAC \longrightarrow P-PDE + PKAC}$		
M011	Reaction	F12, F13	[39,40,25]
	PP2B dephosphorylation: PP2B PKAR binding		
	$\mathrm{AKAP-CAMP-CAMP-P-PKAR + PP2B \rightleftharpoons AKAP-CAMP-CAMP-P-PKAR-PP2B}$		
M012	Reaction	F14	[41,40,25]
	PP2B dephosphorylation: PP2B PKAR dephosphorylation		
	$\mathrm{AKAP-CAMP-CAMP-P-PKAR-PP2B \longrightarrow PP2B + AKAP-CAMP-CAMP-PKAR}$		
M013	Reaction	F12, F13	[39,40,18]
	PP2B dephosphorylation: PP2B AQP2-P binding		
	$\mathrm{AQP2-P + PP2B \rightleftharpoons AQP2-P-PP2B}$		
M014	Reaction	F14	[41,40,18]
	PP2B dephosphorylation: PP2B AQP2-P dephosphorylation		
	$\mathrm{AQP2-P-PP2B \longrightarrow PP2B + AQP2}$		
M015	Reaction	F15, F16	[34,42]
	cAMP regulation: cAMP to AMP catalysis by PDE4		
	$\mathrm{CAMP \xrightarrow{PDE} AMP}$		
M016	Reaction	F17, F18	[34,42]
	cAMP regulation: cAMP to AMP catalysis by PDE4-P		
	$\mathrm{CAMP \xrightarrow{P-PDE} AMP}$		
M017	Reaction	F19	[43]
	cAMP regulation: cAMP influx		
	$\mathrm{\longrightarrow CAMP}$		

Supplementary Table 2. Phosphorylation model quantitative parameters.

ID	Type	Content	Unit	Evidence
F01	SecondOrderForwardsRateConstant	2.1	l/(s·µmol)	[20]
	PKAR binding PKAC			
F02	FirstOrderBackwardsRateConstant	3.0×10^{-4}	1/s	[20]
	PKAR releasing PKAC			

Supplementary Table 2. **Phosphorylation model quantitative parameters.**

ID	Type	Content	Unit	Evidence
F03	FirstOrderForwardsRateConstant<br>*PKAC substrate release after conformation change*	5.0×10^{1}	1/s	[33]
F04	SecondOrderForwardsRateConstant<br>*PKAR-P binding PKAC*	3.8×10^{-2}	l/(s·µmol)	[20]
F05	FirstOrderBackwardsRateConstant<br>*PKAR-P releasing PKAC*	2.6×10^{-4}	1/s	[20]
F06	SecondOrderForwardsRateConstant<br>*cAMP binding to PKAR for both pockets A and B*	5.0×10^{-2}	l/(s·µmol)	[21,40]
F07	FirstOrderBackwardsRateConstant<br>*PKAR releases cAMP from pocket B*	2.6×10^{-6}	1/s	[22,40]
F08	FirstOrderBackwardsRateConstant<br>*PKAR releases cAMP from pocket A*	6.3×10^{-2}	1/s	[22,40]
F09	SecondOrderForwardsRateConstant<br>*PKAC AQP2 binding*	1.5	l/(s·µmol)	[35,36]
F10	FirstOrderBackwardsRateConstant<br>*PKAC substrate release*	7.7×10^{-2}	1/s	[35,36]
F11	SecondOrderForwardsRateConstant<br>*PKAC PDE4 binding*	1.5	l/(s·µmol)	[35,36]
F12	SecondOrderForwardsRateConstant<br>*PP2B substrate binding*	2.5×10^{-2}	l/(s·µmol)	[39,40]
F13	FirstOrderBackwardsRateConstant<br>*PP2B substrate release*	1.0×10^{-2}	1/s	[39,40]
F14	FirstOrderForwardsRateConstant<br>*PP2B substrate dephosphorylation and release*	5.0×10^{-1}	1/s	[41,40]
F15	MichaelisConstant<br>*PDE4 affinity of cAMP*	5.9	µmol/l	[42]
F16	TurnoverNumber<br>*PDE4 turnover of cAMP*	4.5×10^{-2}	1/s	[42]
F17	MichaelisConstant<br>*PDE4-P affinity of cAMP*	1.2	µmol/l	[42]
F18	TurnoverNumber<br>*PDE4-P turnover of cAMP*	2.3	1/s	[42]
F19	ZeroOrderForwardsRateConstant<br>*CAMP influx*	1.0×10^{-2}	µmol/(s·l)	estimation
F20	InitialConcentration<br>*of entity CAMP in cytoplasm*	1.0×10^{-1}	µmol/l	[42]
F21	InitialConcentration<br>*of entity AKAP-P-PKAC-PKAR in vesicular membrane*	2.0×10^{-1}	µmol/l	[24]
F22	InitialConcentration<br>*of entity AKAP-P-PKAR in vesicular membrane*	2.0	µmol/l	[24]

Supplementary Table 2. **Phosphorylation model quantitative parameters.**

ID	Type	Content	Unit	Evidence
F23	InitialConcentration *of entity PDE in vesicular membrane*	2.0×10^{-1}	µmol/l	[42]
F24	InitialConcentration *of entity P-PDE in vesicular membrane*	1.0×10^{-1}	µmol/l	estimation
F25	InitialConcentration *of entity PP2B in vesicular membrane*	2.0×10^{-1}	µmol/l	estimation
F26	InitialConcentration *of entity AQP2 in vesicular membrane*	5.9	µmol/l	[28], [13]
F27	InitialConcentration *of entity AQP2-P vesicular membrane*	2.7	µmol/l	[28], [44]

Supplementary Table 3. **Phosphorylation model variations.**

ID	Values	Unit	Σ
F01	`1.05e+00, 2.10e+00, 4.20e+00`	l/(s·µmol)	3
F06	`1.00e-03, 1.00e-02, 7.00e-02, 2.00e-01`	l/(s·µmol)	4
F09	`7.50e-01, 1.50e+00, 3.00e+00`	l/(s·µmol)	3
F11	`7.50e-01, 1.50e+00, 3.00e+00`	l/(s·µmol)	3
F12	`5.00e-03, 1.00e-02, 5.00e-02, 1.00e-01`	l/(s·µmol)	4
F18	`1.17e+00, 2.34e+00, 4.68e+00`	1/s	3
F19	`1.00e-02, 5.00e-02, 1.00e-01, 2.00e-01`	µmol/(s·l)	4
		Total variations	5184

1.2 cAMP diffusion

cAMP signaling is compartmentalized The amplitude, duration and localization of a response to a signal is determined by several proteins that manage the synthesis and degradation of messenger molecules[45]. The vasopressin response mainly revolves around the second messenger cAMP which activates PKA. The basal average cAMP concentration is about 1 $\mu mol\,L^{-1}$ and the reported concentration to half-maximally activate PKA *in vitro* is about 200 $nmol\,L^{-1}$[42]. This relationship suggests that PKA should be constantly active and unable to respond to signals. However, measurements *in vivo* determined the sensitivity of PKA to be about twenty times lower. Different explanations can be found for this phenomenon[42]. A promising hypothesis for the apparent low sensitivity is the highly regulated cAMP abundance in compartmentalized pools[46–48]. Multiple factors contribute to the compartmentalization of cAMP[43]. First, phosphodiesterases have been shown to control local cAMP concentration for the regulation of PKA[49]. Different isoforms of PDE are varying in localization, specificity, and rate of cyclic nucleotide degradation[42]; the variant that is located close to AQP2 vesicles is PDE4[37]. The work by Stefan et al. shows that AKAP18δ directly interacts with PDE4, tethers it to AQP2 bearing vesicles, and even co-translocates with the vesicles. Interestingly, a phosphorylation at Ser54 by PKA causes an increase in its activity[34]. The concentration of PDE4 in cells is in the same range as catalytic subunits of PKA $\mu mol\,L^{-1}$, further substantiating their co-dependence. Another large factor to achieve efficient compartmentalization of PKA is the reduction of the diffusivity of cAMP. The diffusivity of cAMP in cytoplasm of myocytes was determined to be 32 $cm^2\,s^{-1}$, which is about a magnitude slower than in water[50]. Richards and colleges also determined that the low diffusivity is primarily contributed by tortuosity, which is the obstruction of an objects' path due to physical barriers (such as other molecules, filaments, and organelles) in fluids. Other studies have shown that the location and surrounding of cAMP in the cell has major influence on the diffusivity of cAMP[51,52]. The buffering of cAMP due to binding enzymes also influences the concentration of cAMP that is distributed throughout the cell[52]. Computational and experimental studies have shown that depending on cell type and signaling cascade, the different factors vary in intensity[47,53–55].

Simplifications and estimations PKA and cAMP are known to be involved in fine-tuned spatial signaling complexes[56]. However, it is unclear how PKA is able to maintain a high specificity while being so sensitive to cAMP[42]. Therefore, we wanted to explore the behavior of this signaling in different spatial environments. Two setups were developed that are used to observe cAMP compartmentalization in restricted and unrestricted environments. Ubiquitous phosphodiesterase were modeled implicitly by choosing a low basal cAMP diffusion rate[50]. This accounts for the steady degradation of cAMP in the cytoplasm of the cell. The cAMP gradient was calculated by $[\mathrm{cAMP}]_{\mathrm{basal}} - [\mathrm{cAMP}]_{\mathrm{storage}}$, PKA activity was calculated by $[\mathrm{PKA}]_{\mathrm{free}}/([\mathrm{PKA}]_{\mathrm{free}} + [\mathrm{PKA}]_{\mathrm{bound}})$, similar to the AQP2 phosphorylation ratio $[\mathrm{AQP2\text{-}P}]/([\mathrm{AQP2}] + [\mathrm{AQP2\text{-}P}])$. Buffering is explicitly considered by the PKA regulation mechanism. Additional sources of cAMP buffering were not considered. An area of restricted diffusion was introduced to consider localized reduction of cAMP diffusivity close to vesicles stored in the perinuclear region[37]. The reduced diffusivity can be a result of transitional binding, molecular crowding, or dense cytofilaments[52]. These factors have been combined to a permeability coefficient. Additionally, some regions of the cell are restricted by impassible membrane-enclosed organelles. The diffusive restriction can therefore be altered by increasing the number of obstacles that block access to vesicles. Hence, a spatial setup was designated, where the storage region of vesicles is isolated from the cytoplasm using a membrane-based barrier. The number of passages in the barrier is varied and used to model the influence of different degrees of restricted access. This reduces the total accessible area of originally 3.1 μm^2, to about 0.4, 0.3, and 0.2 μm^2 evenly distributed across 4, 3, and 2 access points, respectively. Total simulation time was 5 minutes.

Supplementary Table 4. **cAMP compartmentalization model modules.**

ID	Type	Features	Evidence
M001	Reaction	F01, F02	[32,20]
	PKA activation: PKAR PKAC binding and phosphorylation		
	PKAC + AKAP−CAMP−PKAR $\rightleftharpoons$ AKAP−CAMP−P−PKAC−PKAR		
	PKAC + AKAP−PKAR $\rightleftharpoons$ AKAP−P−PKAC−PKAR		
	PKAC + AKAP−CAMP−CAMP−PKAR $\rightleftharpoons$ AKAP−CAMP−CAMP−P−PKAC−PKAR		
M002	Reaction	F03	[33,20]
	PKA activation: PKAC PKAR release		
	AKAP−CAMP−CAMP−P−PKAC−PKAR $\longrightarrow$ PKAC + AKAP−CAMP−CAMP−P−PKAR		

Supplementary Table 4. cAMP compartmentalization model modules.

ID	Type	Features	Evidence
M003	Reaction	F04, F05	[23]
	PKA activation: PKAR-P PKAC binding		
	PKAC + AKAP−CAMP−P−PKAR $\rightleftharpoons$ AKAP−CAMP−P−PKAC−PKAR		
	PKAC + AKAP−CAMP−CAMP−P−PKAR $\rightleftharpoons$ AKAP−CAMP−CAMP−P−PKAC−PKAR		
	PKAC + AKAP−P−PKAR $\rightleftharpoons$ AKAP−P−PKAC−PKAR		
M004	Reaction	F06, F07	[23,21,22]
	PKA activation: PKAR CAMP pocket B binding		
	CAMP + AKAP−PKAR $\rightleftharpoons$ AKAP−CAMP−PKAR		
	CAMP + AKAP−P−PKAR $\rightleftharpoons$ AKAP−CAMP−P−PKAR		
	CAMP + AKAP−P−PKAC−PKAR $\rightleftharpoons$ AKAP−CAMP−P−PKAC−PKAR		
M005	Reaction	F06, F08	[23,21,22]
	PKA activation: PKAR CAMP pocket A binding and PKAC release		
	CAMP + AKAP−CAMP−P−PKAC−PKAR $\rightleftharpoons$ PKAC + AKAP−CAMP−CAMP−P−PKAR		
M006	Reaction	F06, F08	[23,21,22]
	PKA activation: PKAR CAMP pocket A binding		
	CAMP + AKAP−CAMP−P−PKAR $\rightleftharpoons$ AKAP−CAMP−CAMP−P−PKAR		
	CAMP + AKAP−CAMP−PKAR $\rightleftharpoons$ AKAP−CAMP−CAMP−PKAR		
M007	Reaction	F09, F10	[34,27,35,36]
	PKA phosphorylation: PKAC AQP2 binding		
	AQP2 + PKAC $\rightleftharpoons$ AQP2 −PKAC		
M008	Reaction	F03	[27,35,36]
	PKA phosphorylation: PKAC AQP2 phosphorylation and release		
	AQP2 −PKAC $\longrightarrow$ AQP2 −P + PKAC		
M009	Reaction	F11, F10	[34,37,38]
	PKA phosphorylation: PKAC PDE4 binding		
	PDE + PKAC $\rightleftharpoons$ PDE−PKAC		
M010	Reaction	F03	[34,37,38]
	PKA phosphorylation: PKAC PDE4 phosphorylation and release		
	PDE−PKAC $\longrightarrow$ P−PDE + PKAC		
M011	Reaction	F12, F13	[39,40,25]
	PP2B dephosphorylation: PP2B PKAR binding		
	AKAP−CAMP−CAMP−P−PKAR + PP2 B $\rightleftharpoons$ AKAP−CAMP−CAMP−P−PKAR−PP2 B		
M012	Reaction	F14	[41,40,25]
	PP2B dephosphorylation: PP2B PKAR dephosphorylation		
	AKAP−CAMP−CAMP−P−PKAR−PP2 B $\longrightarrow$ PP2 B + AKAP−CAMP−CAMP−PKAR		
M013	Reaction	F12, F13	[39,40,18]
	PP2B dephosphorylation: PP2B AQP2-P binding		
	AQP2 −P + PP2 B $\rightleftharpoons$ AQP2 −P−PP2 B		
M014	Reaction	F14	[41,40,18]
	PP2B dephosphorylation: PP2B AQP2-P dephosphorylation		
	AQP2 −P−PP2 B $\longrightarrow$ PP2 B + AQP2		

Supplementary Table 4. **cAMP compartmentalization model modules.**

ID	Type	Features	Evidence
M015	Reaction	F15, F16	[34,42]
	cAMP regulation: cAMP to AMP catalysis by PDE4		
	$\text{CAMP} \xrightarrow{\text{PDE}} \text{AMP}$		
M016	Reaction	F17, F18	[34,42]
	cAMP regulation: cAMP to AMP catalysis by PDE4-P		
	$\text{CAMP} \xrightarrow{\text{P-PDE}} \text{AMP}$		
M017	Reaction	F19	[43]
	cAMP regulation: cAMP influx		
	$\longrightarrow \text{CAMP}$		
M018	Diffusion	F20, F21, F22, F24	[50]
	cAMP cytoplasm diffusion		

Supplementary Table 5. **cAMP compartmentalization quantitative parameters**

ID	Type	Content	Unit	Evidence
F01	SecondOrderForwardsRateConstant	2.1	l/(s·µmol)	[20]
	PKAR binding PKAC			
F02	FirstOrderBackwardsRateConstant	3.0×10^{-4}	1/s	[20]
	PKAR releasing PKAC			
F03	FirstOrderForwardsRateConstant	5.0×10^{1}	1/s	[33]
	PKAC substrate release after conformation change			
F04	SecondOrderForwardsRateConstant	3.8×10^{-2}	l/(s·µmol)	[20]
	PKAR-P binding PKAC			
F05	FirstOrderBackwardsRateConstant	2.6×10^{-4}	1/s	[20]
	PKAR-P releasing PKAC			
F06	SecondOrderForwardsRateConstant	5.0×10^{-2}	l/(s·µmol)	[21,40]
	cAMP binding to PKAR for both pockets A and B			
F07	FirstOrderBackwardsRateConstant	2.6×10^{-6}	1/s	[22,40]
	PKAR releases cAMP from pocket B			
F08	FirstOrderBackwardsRateConstant	6.3×10^{-2}	1/s	[22,40]
	PKAR releases cAMP from pocket A			
F09	SecondOrderForwardsRateConstant	1.5	l/(s·µmol)	[35,36]
	PKAC AQP2 binding			
F10	FirstOrderBackwardsRateConstant	7.7×10^{-2}	1/s	[35,36]
	PKAC substrate release			
F11	SecondOrderForwardsRateConstant	1.5	l/(s·µmol)	[35,36]
	PKAC PDE4 binding			
F12	SecondOrderForwardsRateConstant	2.5×10^{-2}	l/(s·µmol)	[39,40]
	PP2B substrate binding			
F13	FirstOrderBackwardsRateConstant	1.0×10^{-2}	1/s	[39,40]
	PP2B substrate release			

Supplementary Table 5. **cAMP compartmentalization quantitative parameters**

ID	Type	Content	Unit	Evidence
F14	FirstOrderForwardsRateConstant<br>*PP2B substrate dephosphorylation and release*	5.0×10^{-1}	1/s	[41],[40]
F15	TurnoverNumber<br>*PDE4 turnover of cAMP*	4.5×10^{-2}	1/s	[42]
F16	MichaelisConstant<br>*PDE4 affinity of cAMP*	5.9	µmol/l	[42]
F17	TurnoverNumber<br>*PDE4-P turnover of cAMP*	2.3	1/s	[42]
F18	MichaelisConstant<br>*PDE4-P affinity of cAMP*	1.2	µmol/l	[42]
F19	ZeroOrderForwardsRateConstant<br>*CAMP influx*	1.0×10^{-2}	µmol/(s·l)	estimation
F22	Permeability<br>*reduced diffusion of cAMP in storage region*	1.0×10^{-1}		[52]
F23	MembraneDiffusivity<br>*lateral diffusivity of membrane bound entities*	4.3	µm²/s	[57],[58]
F24	ConcentrationDiffusivity<br>*cAMP diffusivity in cytoplasm*	3.2×10^{1}	µm²/s	[50]
F25	SpatialDiffusivity<br>*diffusivity of macroscopic entities*	1.5×10^{-6}	µm²/s	[59],[60]
F26	InitialConcentration<br>*of entity CAMP in cytoplasm*	5.0×10^{-1}	µmol/l	[42]
F27	InitialConcentration<br>*of entity CAMP in restricted region*	1.0×10^{-1}	µmol/l	[42]
F28	InitialConcentration<br>*of entity AKAP-P-PKAC-PKAR in vesicle membrane*	2.0×10^{-1}	µmol/l	[24]
F29	InitialConcentration<br>*of entity AKAP-P-PKAR in vesicle membrane*	2.0	µmol/l	[24]
F30	InitialConcentration<br>*of entity PDE in vesicle membrane*	5.0×10^{-2}	µmol/l	[42]
F31	InitialConcentration<br>*of entity P-PDE in vesicle membrane*	5.0×10^{-2}	µmol/l	estimation
F32	InitialConcentration<br>*of entity PP2B in vesicle membrane*	2.0×10^{-1}	µmol/l	estimation
F33	InitialConcentration<br>*of entity AQP2 in vesicle membrane*	2.0×10^{1}	µmol/l	[28],[13]
F34	InitialConcentration<br>*of entity AQP2-P in vesicle membrane*	9.8	µmol/l	[28],[13]

Supplementary Table 6. **cAMP compartmentalization qualitative parameters**

ID	Type	Content	Evidence
F20	Cargoes	[CAMP]	
	entities that are subject to diffusion		
F21	AffectedSection	cytoplasm	
	section that is affected by diffusion		

Supplementary Table 7. **cAMP compartmentalization variations**

ID	Values	Unit	Σ
F06	`1.00e-02, 5.00e-02, 1.00e-01`	l/(s·µmol)	3
F12	`2.50e-02, 5.00e-02`	l/(s·µmol)	2
F17	`2.34e+00, 4.68e+00, 9.48e+00`	1/s	3
F19	`1.00e-02, 5.00e-02, 1.00e-01, 2.00e-01`	µmol/(s·l)	4
F22	`1.00e-02, 1.00e-01, 1.00e-00`		3
Setups	`restricted (2, 3, and 4 passages), unrestricted`		4
		Total variations	864

1.3 Vesicle endocytosis

AQP2 is retrieved by Clathrin-mediated endocytosis AQP2 is concentrated in clathrin coated pits[61], indicating that clathrin-mediated endocytosis is the preferred mode of internalization and recycling. In the process of clathrin-mediated endocytosis, nucleation points initialize pit formation at the membrane. Specific adapter proteins are added that bind both the cargo and clathrin molecules. It was found that phosphorylation of AQP2 at Serine 269 inhibits the binding of the adapter protein Sipa1l1, blocks endocytosis[62,63], and generally alters interactions with other proteins of the endocytosis machinery[26]. Serine 269 is phosphorylated in response to vesopressin[64] and only found in the apical membrane[6]. Another mechanism that affects vesicle internalization of AQP2 is the phosphorylation of Proto-oncogene tyrosine-protein kinase Src (SRC)[65]. The exact mechanism is not fully understood, but it is speculated that SRC phosphorylation of Dynamin is required for vesicle scission. Its inhibition leads to AQP2 retention[65]. The connection between PKA and SRC can be made via C-terminal Src kinase (CSK)[66]. SRC has two phosphorylation sites: Tyrosine 416 for autophosphorylation and activation of the kinase and Tyrosine 527 for inhibition[67]. PKA phosphorylates CSK at Serine 364, which in turn phosphorylates SRC at 527. Therefore, an activation of PKA in the apical region of the cell would also lead to a decrease in vesicle formation and therefore AQP2 accumulation. Loerke et al. discerned distinct subpopulations of clathrin coated pits that correspond to aborted intermediate pits and vesicle-forming, long-lived pits[68]. They also found that the addition of cargo determined whether a pit would be aborted after a certain time or matured to become a vesicle. After the vesicle is detached from the membrane, it experiences an abrupt lateral displacement[69].

Simplifications and estimations Endocytosis is primarily designed after the model by Loekre at al.[68]. We assume that "seeds" for clathrin-coated pits form randomly on the membrane surface. During their lifetime, clathrin-coated pits successively pass two stages: collection and maturation. During the collection phase, pits gather chemical entities from their surrounding membrane. After a predefined checkpoint time the concentration of a key cargo entities is checked and if it surpasses a threshold value the vesicle enters the maturation stage. If the threshold was not reached, the pit enters the abortion stage and the accumulated chemical entities pass back to the membrane. After maturation time, each pit forms a clathrin-coated vesicle. The endocytotic pit formation rate was converted to a probability, that a vesicle would spawn in a given membrane segment and time step. Loekre and colleagues observed three subpopulations of pits: two short-lived and one long-lived. In this model, early-abortive and late-abortive subpopulations are not distinguished. We considered only the late abortive population, since not enough information on the factors that drive their distinction is available.

In the apical cell membrane, SRC regulates the endocytosis of AQP2 positive vesicles[65]. The exact mechanism is still under speculation, but it is proposed that active SRC is required for the Dynamin mediated scission of pits to form vesicles. Another possibility is that phosphorylation of key cargo proteins inhibits the binding of adaptor proteins such as Sipa1l1[62] or AP2[68]. In this model, the ratio of inhibited SRC kinase and active SRC kinase was used to scale the cargo accumulation rate, such that inhibited SRC kinase leads to an increase in abortive pits. The inhibition of SRC is mediated by a phosphorylation of Tyrosine 527, which is performed by activated CSK[67]. Csk itself can be activated by PKA via phosphorylation of Serine 364[70].

Therefore, an activation of PKA leads to an inactivation of SRC and subsequently to AQP2 retention in the apical membrane. This pathway coincides with the observations of Cheung et al., who found out that an inhibition of SRC is sufficient to cause AQP2 accumulation in the membrane[65]. PKA not only activates the transport from intracellular storage, but also indirectly inhibits AQP2 removal from the membrane. After endocytotic pits have fully matured, they are scissioned and experience an abrupt lateral displacement[69] with an average speed of 57 $\mathrm{nm\,s^{-1}}$ for about 11 s.

Supplementary Table 8. **Endocytosis model modules**

ID	Type	Features	Evidence
M001	Reaction	F01, F02	32,20
	PKA activation: PKAR PKAC binding and phosphorylation		
	PKAC + AKAP−CAMP−PKAR ⇌ AKAP−CAMP−P−PKAC−PKAR		
	PKAC + AKAP−PKAR ⇌ AKAP−P−PKAC−PKAR		
	PKAC + AKAP−CAMP−CAMP−PKAR ⇌ AKAP−CAMP−CAMP−P−PKAC−PKAR		
M002	Reaction	F03	33,20
	PKA activation: PKAC PKAR release		
	AKAP−CAMP−CAMP−P−PKAC−PKAR ⟶ PKAC + AKAP−CAMP−CAMP−P−PKAR		
M003	Reaction	F04, F05	23
	PKA activation: PKAR-P PKAC binding		
	PKAC + AKAP−CAMP−P−PKAR ⇌ AKAP−CAMP−P−PKAC−PKAR		
	PKAC + AKAP−CAMP−CAMP−P−PKAR ⇌ AKAP−CAMP−CAMP−P−PKAC−PKAR		
	PKAC + AKAP−P−PKAR ⇌ AKAP−P−PKAC−PKAR		
M004	Reaction	F06, F07	23,21,22
	PKA activation: PKAR CAMP pocket B binding		
	CAMP + AKAP−PKAR ⇌ AKAP−CAMP−PKAR		
	CAMP + AKAP−P−PKAR ⇌ AKAP−CAMP−P−PKAR		
	CAMP + AKAP−P−PKAC−PKAR ⇌ AKAP−CAMP−P−PKAC−PKAR		
M005	Reaction	F08, F07	23,21,22
	PKA activation: PKAR CAMP pocket A binding and PKAC release		
	CAMP + AKAP−CAMP−P−PKAC−PKAR ⇌ PKAC + AKAP−CAMP−CAMP−P−PKAR		
M006	Reaction	F08, F07	23,21,22
	PKA activation: PKAR CAMP pocket A binding		
	CAMP + AKAP−CAMP−P−PKAR ⇌ AKAP−CAMP−CAMP−P−PKAR		
	CAMP + AKAP−CAMP−PKAR ⇌ AKAP−CAMP−CAMP−PKAR		
M007	Reaction	F09, F10	34,27,35,36
	PKA phosphorylation: PKAC AQP2 binding		
	AQP2 + PKAC ⇌ AQP2 −PKAC		
M008	Reaction	F03	27,35,36
	PKA phosphorylation: PKAC AQP2 phosphorylation and release		
	AQP2 −PKAC ⟶ AQP2 −P + PKAC		
M009	Reaction	F09, F11	34,37,38
	PKA phosphorylation: PKAC PDE4 binding		
	PDE + PKAC ⇌ PDE−PKAC		
M010	Reaction	F03	34,37,38
	PKA phosphorylation: PKAC PDE4 phosphorylation and release		
	PDE−PKAC ⟶ P−PDE + PKAC		

Supplementary Table 8. **Endocytosis model modules**

ID	Type	Features	Evidence
M011	Reaction	F12, F13	39, 40, 25
	PP2B dephosphorylation: PP2B PKAR binding		
	$\mathrm{AKAP{-}CAMP{-}CAMP{-}P{-}PKAR + PP2\,B \rightleftharpoons AKAP{-}CAMP{-}CAMP{-}P{-}PKAR{-}PP2\,B}$		
M012	Reaction	F14	41, 40, 25
	PP2B dephosphorylation: PP2B PKAR dephosphorylation		
	$\mathrm{AKAP{-}CAMP{-}CAMP{-}P{-}PKAR{-}PP2\,B \longrightarrow PP2\,B + AKAP{-}CAMP{-}CAMP{-}PKAR}$		
M013	Reaction	F12, F13	39, 40, 18
	PP2B dephosphorylation: PP2B AQP2-P binding		
	$\mathrm{AQP2\,{-}P + PP2\,B \rightleftharpoons AQP2\,{-}P{-}PP2\,B}$		
M014	Reaction	F14	41, 40, 18
	PP2B dephosphorylation: PP2B AQP2-P dephosphorylation		
	$\mathrm{AQP2\,{-}P{-}PP2\,B \longrightarrow PP2\,B + AQP2}$		
M015	Reaction	F09, F10	65, 70
	Src phosphorylation: PKAC CSK binding		
	$\mathrm{CSK + PKAC \rightleftharpoons CSK{-}PKAC}$		
M016	Reaction	F03	65, 70
	Src phosphorylation: PKAC CSK phosphorylation and release		
	$\mathrm{CSK{-}PKAC \longrightarrow CSK{-}P + PKAC}$		
M017	Reaction	F15, F16	71, 67, 72
	Src phosphorylation: CSK SRC binding		
	$\mathrm{SRC + CSK{-}P \rightleftharpoons CSK{-}P{-}SRC}$		
M018	Reaction	F17	71, 67, 72
	Src phosphorylation: CSK SRC phosphorylation and release		
	$\mathrm{CSK{-}P{-}SRC \longrightarrow P{-}SRC + CSK{-}P}$		
M019	Reaction	F18, F19	34, 42
	cAMP regulation: cAMP to AMP catalysis by PDE4		
	$\mathrm{CAMP \xrightarrow{PDE} AMP}$		
M020	Reaction	F20, F21	34, 42
	cAMP regulation: cAMP to AMP catalysis by PDE4-P		
	$\mathrm{CAMP \xrightarrow{P{-}PDE} AMP}$		
M021	Reaction	F22	43
	cAMP regulation: cAMP influx		
	$\mathrm{\longrightarrow CAMP}$		
M022	EndocytoticPitAbsorption	F23, F24, F25	68
	endocytosis: pit cargo collection		
M023	ClathrinMediatedEndocytosis	F25, F26, F27, F31, F32, F33, F34, F35, F36	68, 73
	endocytosis: aqp2 vesicle endocytosis		

Supplementary Table 9. **Endocytosis model quantitative parameters**

ID	Type	Content	Unit	Evidence
F01	FirstOrderBackwardsRateConstant<br>*PKAR releasing PKAC*	3.0×10^{-4}	1/s	[20]
F02	SecondOrderForwardsRateConstant<br>*PKAR binding PKAC*	2.1	l/(s·µmol)	[20]
F03	FirstOrderForwardsRateConstant<br>*PKAC substrate release after conformation change*	5.0×10^{1}	1/s	[33]
F04	FirstOrderBackwardsRateConstant<br>*PKAR-P releasing PKAC*	2.6×10^{-4}	1/s	[20]
F05	SecondOrderForwardsRateConstant<br>*PKAR-P binding PKAC*	3.8×10^{-2}	l/(s·µmol)	[20]
F06	FirstOrderBackwardsRateConstant<br>*PKAR releases cAMP from pocket B*	2.6×10^{-6}	1/s	[22,40]
F07	SecondOrderForwardsRateConstant<br>*cAMP binding to PKAR for both pockets A and B*	1.0×10^{-2}	l/(s·µmol)	[21,40]
F08	FirstOrderBackwardsRateConstant<br>*PKAR releases cAMP from pocket A*	6.3×10^{-2}	1/s	[22,40]
F09	FirstOrderBackwardsRateConstant<br>*PKAC substrate release*	7.7×10^{-2}	1/s	[35,36]
F10	SecondOrderForwardsRateConstant<br>*PKAC AQP2 binding*	1.5	l/(s·µmol)	[35,36]
F11	SecondOrderForwardsRateConstant<br>*PKAC PDE4 binding*	1.5	l/(s·µmol)	[35,36]
F12	FirstOrderBackwardsRateConstant<br>*PP2B substrate release*	1.0×10^{-2}	1/s	[39,40]
F13	SecondOrderForwardsRateConstant<br>*PP2B substrate binding*	1.0×10^{-2}	l/(s·µmol)	[39,40]
F14	FirstOrderForwardsRateConstant<br>*PP2B substrate dephosphorylation and release*	5.0×10^{-1}	1/s	[41,40]
F15	FirstOrderBackwardsRateConstant<br>*CSK substrate release*	7.0×10^{-1}	1/s	[72]
F16	SecondOrderForwardsRateConstant<br>*CSK substrate binding*	1.0	l/(µmol·s)	[72]
F17	FirstOrderForwardsRateConstant<br>*CSK substrate phosphorylation and release*	2.0	1/s	[72]
F18	MichaelisConstant<br>*PDE4 affinity of cAMP*	5.9	µmol/l	[42]
F19	TurnoverNumber<br>*PDE4 turnover of cAMP*	4.5×10^{-2}	1/s	[42]
F20	MichaelisConstant<br>*PDE4-P affinity of cAMP*	1.2	µmol/l	[42]

Supplementary Table 9. **Endocytosis model quantitative parameters**

ID	Type	Content	Unit	Evidence
F21	TurnoverNumber<br>*PDE4-P turnover of cAMP*	2.3	1/s	[42]
F22	ZeroOrderForwardsRateConstant<br>*CAMP influx*	3.0×10^{-1}	μmol/(s·l)	estimation
F24	CargoAdditionRate<br>*rate at which relevant cargo is added to the pit*	4.0×10^{-2}	1/s	estimation
F26	VesicleRadius<br>*radius of AQP2 positive vesicles*	5.0×10^{-2}	μm	[69]
F28	InitialConcentration<br>*of entity CLA in vesicle membrane*	1.0×10^{-1}	μmol/l	[69]
F29	InitialConcentration<br>*of entity DYN in vesicle membrane*	8.3×10^{-3}	μmol/l	[74]
F30	InitialConcentration<br>*of entity MYO in vesicle membrane*	1.7×10^{-2}	μmol/l	[75]
F31	PitFormationRate<br>*rate at which new pits form*	5.0×10^{-2}	1/(s·μm²)	[69]
F33	EndocytosisCheckpointConcentration<br>*a pit matures, if this number of cargo molecules is reached*	6.5×10^{2}	molecules	[68]
F34	EndocytosisCheckpointTime<br>*time after pit formation that determines if a pit matures*	3.0×10^{1}	s	[68]
F36	MaturationTime<br>*the average time of the endocytotic maturation process*	7.0×10^{1}	s	[68]
F37	MembraneDiffusivity<br>*lateral diffusivity of membrane bound entities*	4.3	μm²/s	[57,58]
F38	ConcentrationDiffusivity<br>*cAMP diffusivity in cytoplasm*	3.2×10^{1}	μm²/s	[50]
F39	InitialConcentration<br>*of entity CAMP in cytoplasm*	5.0×10^{-1}	μmol/l	[42]
F40	InitialConcentration<br>*of entity AQP2 in apical plasma membrane*	5.0×10^{-1}	μmol/l	[28,13]
F41	InitialConcentration<br>*of entity AQP2-P in apical plasma membrane*	1.0×10^{1}	μmol/l	[28,44]
F42	InitialConcentration<br>*of entity AKAP-P-PKAC-PKAR in apical plasma membrane*	1.9×10^{-1}	μmol/l	[24]
F43	InitialConcentration<br>*of entity PKAC in apical plasma membrane*	1.0×10^{-2}	μmol/l	[42,24]
F44	InitialConcentration<br>*of entity AKAP-CAMP-P-PKAR in apical plasma membrane*	2.0	μmol/l	[24]
F45	InitialConcentration<br>*of entity PDE in apical plasma membrane*	1.0×10^{-1}	μmol/l	estimation

Supplementary Table 9. **Endocytosis model quantitative parameters**

ID	Type	Content	Unit	Evidence
F46	InitialConcentration *of entity P-PDE in apical plasma membrane*	2.0×10^{-1}	µmol/l	estimation
F47	InitialConcentration *of entity PP2B in apical plasma membrane*	2.0×10^{-1}	µmol/l	estimation
F48	InitialConcentration *of entity CSK in apical plasma membrane*	2.0×10^{-1}	µmol/l	estimation
F49	InitialConcentration *of entity SRC in apical plasma membrane*	1.0×10^{-1}	µmol/l	estimation
F50	InitialConcentration *of entity P-SRC in apical plasma membrane*	1.0×10^{-1}	µmol/l	estimation

Supplementary Table 10. **Endocytosis model qualitative parameters**

ID	Type	Content	Evidence
F23	ScalingEntities *the entities that influence cargo addition rate*	[SRC, P-SRC]	estimation
F25	Cargoes *other cargoes*	all membrane bound entities	estimation
F27	InitialConcentrations	F28, F29, F30	estimation
F32	AffectedRegion *region where endocytotic pits are able to from*	apical inner plasma membrane (GO:0016324)	estimation
F35	PrimaryCargoes *the entities that are the relevant cargo for this vesicle*	all entities in complex with AQP2	[62]

Supplementary Table 11. **Endocytosis model variations**

ID	Values	Unit	Σ
F16	`4.00e+00, 8.00e+00, 1.60e+01`	l/(µmol·s)	3
F17	`1.00e+00, 2.00e+00, 4.00e+00`	1/s	3
F22	`1.00e-02, 5.00e-02, 1.00e-01, 2.00e-01`	µmol/(s·l)	4
F24	`2.50e-03, 5.00e-03, 1.00e-02, 2.00e-02`	1/s	4
F31	`1.00e-02, 5.00e-02, 1.00e-01, 5.00e-01`	1/(s·µm²)	4
		Total variations	576

1.4 Recycling Model

The recycling model includes the previously refined models in addition to interstellar transport mechanisms to allow vesicle to move between storage compartments and membrane.

AQP2 vesicles translocate to the apical membrane Actin filaments influence the vasopressin response at multiple points during the signaling cascade. In the unattached state, vesicles are able to diffuse into the cytoplasm[76]. Myosin Vb motor and the Rab11-FIP2 adapter protein facilitate the attachment of AQP2 vesicles to filaments and their transport to the apical membrane[75,77]. The average speed of the vesicle during transportation is 710 nm s^{-1}[78]. Upon arrival at the actin cortex, the vesicle is tethered to the actin cortex[10]. Here, myosin II seems to manage transport at and through the cortex[79]. Another study has found that the actin cortex is able to regulate the passage of the AQP2 vesicle[80]. The phosphorylation at Serine 256 seems to decrease the stability and therefore increase permeability of the actin cortex.

Vesicle fusion is mediated by SNARE proteins Vesicle fusion is mediated by a complex array of molecular machinery. A critical component are SNARE proteins[81]. SNARE components mediate fusion through spontaneous connection, one part of the complex resides in the target membrane and acts as an adapter. The vesicle contains the remaining part of the complex that connects to the adapter part, tethering it to the membrane. In the case of AQP2 vesicles, the fusion is accomplished by the interaction of VAMP2 and VAMP3[82, 83] at the vesicle membrane and Syntaxin 3[84] as well as SNAP23 located in the apical membrane[85]. The work of Donovan and Bretscher compiles a timeline for the exocytosis of secretory vesicles[86]. Vesicles remained attached for 18 seconds before vesicle and target membrane were connected.

Vesicle transport at microtubules The transport from the membrane back to the perinuclear region is accomplished by microtubules[2] and dynein[87]. Rab11, potentially in complex with FIP3[88], mediates interaction with the dynein light intermediate chain 1, creating a complex for directed transport to the centrosomal region. The speed of this movement is about $800\,\mathrm{nm\,s^{-1}}$[89]. In current theory, early endosomes arise from the fusion of primary endocytotic vesicles (such as the AQP2 bearing vesicles) while trafficking across the cell on microtubules to the perinuclear region[90]. The sorting and reconstitution of vesicle cargo is an important step to be pondered. It was suggested that dephosphorylation of S256 occurs during vesicular routing[91].

Simplifications and estimations Vesicles are triggered to leave the storage region after AQP2 concentration passes a threshold. These vesicles will be attached to actin filaments[75, 79], if they have the required myosin transporter complex in their membrane and the vesicle is in close proximity to the actin filament. Subsequently, directed vesicle transport is initiated, leading the vesicle along the actin filament. The filaments are generated using an adapted version of the filament growth algorithm by[92]. After the vesicles traversed the distance from storage to apical membrane, they are tethered to the actin cortex[76, 93]. The actin cortex was modeled by adding a thin volume-like agent in front of the membrane. Whenever a vesicle enters this volume-like agent, its state changes, and it is allowed to diffuse throughout the area with reduced diffusivity. This process of actin remodeling was not explicitly modeled. It would be interesting to inspect the actin cortex more closely, to evaluate the possible connections of tropomyosin[80], SRC kinase[94] and Myosin-V[10]. The process requires the correct SNARE molecules to be present in the apical membrane and the vesicle membrane[81]. The corresponding SNARE molecules form a complex, and the vesicle is tethered to its current position at the membrane for an average of 18 seconds[86]. The completion of the fusion process leads to the addition of the chemical entities from the vesicular membrane compartment to the apical membrane compartment. After endocytosis, vesicles are propelled into the cytoplasm, where they attach to microtubules, if they are close enough and contain the protein machinery to do so. Upon arrival in the centrosomal region, they detach from the actin filament. For the purposes of this model, we omitted the sorting in early endosomes and recycling endosomes. We evaluated that this step is not as crucial in the current model, since only one cargo molecule (AQP2) was modeled. Nevertheless, this would be an interesting interface to evaluate the effect multiple cargo molecules and temporary storage compartments have on the recycling of vesicles.

As a spatial setup, we used a 23 x 15 grid with a width of 7.6 µm and a height of 5 µm. Two membranes were defined that represent the apical cell membrane and a membrane separating a vesicular storage region. The system was initialized with total 14 vesicles in the storage region, 7 actin filaments and 14 microtubules. Two regions of restricted diffusion were defined for the storage region and the cellular cortex close to the apical membrane. The system was simulated for a total of 60 minutes.

Supplementary Table 12. Recycling model modules

ID	Type	Features	Evidence
M001	Reaction	F001, F002	[32, 20]
	PKA activation: PKAR PKAC binding and phosphorylation		
	PKAC + AKAP−CAMP−PKAR ⇌ AKAP−CAMP−P−PKAC−PKAR		
	PKAC + AKAP−PKAR ⇌ AKAP−P−PKAC−PKAR		
	PKAC + AKAP−CAMP−CAMP−PKAR ⇌ AKAP−CAMP−CAMP−P−PKAC−PKAR		
M002	Reaction	F003	[33, 20]
	PKA activation: PKAC PKAR release		
	AKAP−CAMP−CAMP−P−PKAC−PKAR → PKAC + AKAP−CAMP−CAMP−P−PKAR		
M003	Reaction	F004, F005	[23]
	PKA activation: PKAR-P PKAC binding		
	PKAC + AKAP−CAMP−P−PKAR ⇌ AKAP−CAMP−P−PKAC−PKAR		
	PKAC + AKAP−CAMP−CAMP−P−PKAR ⇌ AKAP−CAMP−CAMP−P−PKAC−PKAR		
	PKAC + AKAP−P−PKAR ⇌ AKAP−P−PKAC−PKAR		

M004 Reaction F006, F007 [23,21,22]

PKA activation: PKAR CAMP pocket B binding

CAMP + AKAP−PKAR ⇌ AKAP−CAMP−PKAR

CAMP + AKAP−P−PKAR ⇌ AKAP−CAMP−P−PKAR

CAMP + AKAP−P−PKAC−PKAR ⇌ AKAP−CAMP−P−PKAC−PKAR

M005 Reaction F008, F007 [23,21,22]

PKA activation: PKAR CAMP pocket A binding and PKAC release

CAMP + AKAP−CAMP−P−PKAC−PKAR ⇌ PKAC + AKAP−CAMP−CAMP−P−PKAR

M006 Reaction F008, F007 [23,21,22]

PKA activation: PKAR CAMP pocket A binding

CAMP + AKAP−CAMP−P−PKAR ⇌ AKAP−CAMP−CAMP−P−PKAR

CAMP + AKAP−CAMP−PKAR ⇌ AKAP−CAMP−CAMP−PKAR

M007 Reaction F009, F010 [34,27,35,36]

PKA phosphorylation: PKAC AQP2 binding

AQP2 + PKAC ⇌ AQP2 −PKAC

M008 Reaction F003 [27,35,36]

PKA phosphorylation: PKAC AQP2 phosphorylation and release

AQP2 −PKAC ⟶ AQP2 −P + PKAC

M009 Reaction F009, F011 [34,37,38]

PKA phosphorylation: PKAC PDE4 binding

PDE + PKAC ⇌ PDE−PKAC

M010 Reaction F003 [34,37,38]

PKA phosphorylation: PKAC PDE4 phosphorylation and release

PDE−PKAC ⟶ P−PDE + PKAC

M011 Reaction F012, F013 [39,40,25]

PP2B dephosphorylation: PP2B PKAR binding

AKAP−CAMP−CAMP−P−PKAR + PP2 B ⇌ AKAP−CAMP−CAMP−P−PKAR−PP2 B

M012 Reaction F014 [41,40,25]

PP2B dephosphorylation: PP2B PKAR dephosphorylation

AKAP−CAMP−CAMP−P−PKAR−PP2 B ⟶ PP2 B + AKAP−CAMP−CAMP−PKAR

M013 Reaction F012, F013 [39,40,18]

PP2B dephosphorylation: PP2B AQP2-P binding

AQP2 −P + PP2 B ⇌ AQP2 −P−PP2 B

M014 Reaction F014 [41,40,18]

PP2B dephosphorylation: PP2B AQP2-P dephosphorylation

AQP2 −P−PP2 B ⟶ PP2 B + AQP2

M015 Reaction F009, F010 [65,70]

Src phosphorylation: PKAC CSK binding

CSK + PKAC ⇌ CSK−PKAC

M016 Reaction F003 [65,70]

Src phosphorylation: PKAC CSK phosphorylation and release

CSK−PKAC ⟶ CSK−P + PKAC

M017 Reaction F015, F016 [71,67,72]

Src phosphorylation: CSK SRC binding

SRC + CSK−P ⇌ CSK−P−SRC

M018	Reaction<br>*Src phosphorylation: dephosphorylation of CSK*<br>$\mathrm{CSK{-}P} \longrightarrow \mathrm{CSK}$	F017	estimation
M019	Reaction<br>*Src phosphorylation: CSK SRC phosphorylation and release*<br>$\mathrm{CSK{-}P{-}SRC} \longrightarrow \mathrm{P{-}SRC} + \mathrm{CSK{-}P}$	F018	[71,67,72]
M020	Reaction<br>*Src phosphorylation: dephosphorylation of SRC*<br>$\mathrm{P{-}SRC} \longrightarrow \mathrm{SRC}$	F019	estimation
M021	Reaction<br>*cAMP regulation: cAMP to AMP catalysis by PDE4*<br>$\mathrm{CAMP} \xrightarrow{\mathrm{PDE}} \mathrm{AMP}$	F020, F021	[34,42]
M022	Reaction<br>*cAMP regulation: cAMP to AMP catalysis by PDE4-P*<br>$\mathrm{CAMP} \xrightarrow{\mathrm{P{-}PDE}} \mathrm{AMP}$	F022, F023	[34,42]
M023	Reaction<br>*PKA phosphorylation: PDE4 dephosphorylation*<br>$\mathrm{P{-}PDE} \longrightarrow \mathrm{PDE}$	F024	[41,39,40]
M024	Reaction<br>*cAMP regulation: cAMP influx active*<br>$\longrightarrow \mathrm{CAMP}$	F025	[43]
M025	Reaction<br>*cAMP regulation: cAMP influx basal*<br>$\longrightarrow \mathrm{CAMP}$	F026	[43]
M026	Diffusion<br>*cAMP cytoplasm diffusion*	F027, F028, F029	[37,50]
M027	LateralMembraneDiffusion<br>*lateral membrane diffusion*	F030, F031	[58]
M028	VesicleConfinedDiffusion<br>*storage: prevent vesicles from leaving storage region*	F032, F033	[93,95]
M029	VolumeLikeAgentContainment<br>*storage: set state of endocytotic vesicles upon entering storage*	F032, F034, F033	[95]
M030	ConcentrationStateChange<br>*storage: trigger vesicle departure by aqp2 phosphorylation*	F035, F036, F037, F038	[4,13,7]
M031	VesicleCytoplasmDiffusion<br>*exocytosis: vesicle diffusion*		[59,96]
M032	LineLikeAgentAttachment<br>*exocytosis: attach vesicle to actin filament*	F039, F040, F041, F042	[75]
M033	VesicleTransport<br>*exocytosis: transport vesicle along actin filament*	F043, F044	[75]
M034	VolumeLikeAgentContainment<br>*exocytosis: confine tethered diffusion to cortex*	F045, F046, F047	[79,10,80]
M035	VesicleConfinedDiffusion<br>*exocytosis: vesicles entering cortex are tethered*	F045, F047	[79,10,80]

M036	VesicleFusion<br>*exocytosis: vesicle fusion*	F048, F049, F050, F051, F052	[86,82]
M037	Reaction<br>*endocytosis: clathrin release from vesicle surface*<br>CLA $\longrightarrow$	F053	[69]
M038	EndocytoticPitAbsorption<br>*endocytosis: pit cargo collection*	F054, F055, F056	[68]
M039	ClathrinMediatedEndocytosis<br>*endocytosis: aqp2 vesicle endocytosis*	F057, F058, F059, F060, F054, F061, F065, F066, F067	[68,73]
M040	EndocytosisActinBoost<br>*endocytosis: aqp2 vesicle boost*	F068, F069	[69,73]
M041	LineLikeAgentAttachment<br>*endocytosis: microtubule attachment*	F070, F071, F072, F073	[87]
M042	VesicleTransport<br>*endocytosis: microtubule based transport*	F074, F075	[89,87]

Supplementary Table 13. Recycling model quantitative parameters

ID	Type	Content	Unit	Evidence
F001	FirstOrderBackwardsRateConstant<br>*PKAR releasing PKAC*	3.0×10^{-4}	1/s	[20]
F002	SecondOrderForwardsRateConstant<br>*PKAR binding PKAC*	2.1	l/(s·µmol)	[20]
F003	FirstOrderForwardsRateConstant<br>*PKAC substrate release after conformation change*	5.0×10^{1}	1/s	[33]
F004	FirstOrderBackwardsRateConstant<br>*PKAR-P releasing PKAC*	2.6×10^{-4}	1/s	[20]
F005	SecondOrderForwardsRateConstant<br>*PKAR-P binding PKAC*	3.8×10^{-2}	l/(s·µmol)	[20]
F006	FirstOrderBackwardsRateConstant<br>*PKAR releases cAMP from pocket B*	2.6×10^{-6}	1/s	[22,40]
F007	SecondOrderForwardsRateConstant<br>*cAMP binding to PKAR for both pockets A and B*	1.0×10^{-2}	l/(s·µmol)	[21,40]
F008	FirstOrderBackwardsRateConstant<br>*PKAR releases cAMP from pocket A*	6.3×10^{-2}	1/s	[22,40]
F009	FirstOrderBackwardsRateConstant<br>*PKAC substrate release*	7.7×10^{-2}	1/s	[35,36]
F010	SecondOrderForwardsRateConstant<br>*PKAC AQP2 binding*	1.5	l/(s·µmol)	[35,36]
F011	SecondOrderForwardsRateConstant<br>*PKAC PDE4 binding*	1.5	l/(s·µmol)	[35,36]
F012	FirstOrderBackwardsRateConstant<br>*PP2B substrate release*	1.0×10^{-2}	1/s	[39,40]

Supplementary Table 13. Recycling model quantitative parameters

ID	Type	Content	Unit	Evidence
F013	SecondOrderForwardsRateConstant<br>*PP2B substrate binding*	1.0×10^{-2}	l/(s·µmol)	[39],[40]
F014	FirstOrderForwardsRateConstant<br>*PP2B substrate dephosphorylation and release*	5.0×10^{-1}	1/s	[41],[40]
F015	FirstOrderBackwardsRateConstant<br>*CSK substrate release*	7.0×10^{-1}	1/s	[72]
F016	SecondOrderForwardsRateConstant<br>*CSK substrate binding*	1.0	l/(µmol·s)	[72]
F017	FirstOrderForwardsRateConstant<br>*CSK dephosphorylation (constant)*	2.0×10^{-1}	1/s	estimation
F018	FirstOrderForwardsRateConstant<br>*CSK substrate phosphorylation and release*	2.0	1/s	[72]
F019	FirstOrderForwardsRateConstant<br>*SRC dephosphorylation (constant)*	1.0×10^{-1}	1/s	estimation
F020	MichaelisConstant<br>*PDE4 affinity of cAMP*	5.9	µmol/l	[42]
F021	TurnoverNumber<br>*PDE4 turnover of cAMP*	4.5×10^{-2}	1/s	[42]
F022	MichaelisConstant<br>*PDE4-P affinity of cAMP*	1.2	µmol/l	[42]
F023	TurnoverNumber<br>*PDE4-P turnover of cAMP*	2.3	1/s	[42]
F024	FirstOrderForwardsRateConstant<br>*PDE dephosphorylation*	1.0×10^{-1}	1/s	estimation
F025	ZeroOrderForwardsRateConstant<br>*CAMP influx*	3.0×10^{-1}	µmol/(s·l)	estimation
F026	ZeroOrderForwardsRateConstant<br>*CAMP influx*	2.0×10^{-2}	µmol/(s·l)	estimation
F028	Ratio<br>*reduced diffusion of cAMP in storage region*	1.0×10^{-3}		[52]
F038	Ratio<br>*of AQP2-P to AQP2 on the vesicle surface, triggering exocytosis*	1.3		[13]
F040	AttachmentDistance<br>*distance from the vesicle surface to the filament*	5.0×10^{-2}	µm	[97]
F044	MotorMovementVelocity<br>*average velocity of myosin driven transport*	7.1×10^{-1}	µm/s	[78]
F048	AttachmentDistance<br>*distance from the vesicle surface to fusion membrane*	5.0×10^{-2}	µm	[98]
F051	FusionTime<br>*average time per fusion event*	1.8×10^{1}	s	[86]

Supplementary Table 13. Recycling model quantitative parameters

ID	Type	Content	Unit	Evidence
F052	SNAREFusionPairs<br>*average number of snares involved in a fusion event*	8.0		[99]
F053	ZeroOrderForwardsRateConstant<br>*vesicle uncoating rate*	2.4×10^{-1}	µmol/(s·l)	[69]
F055	CargoAdditionRate<br>*rate at which relevant cargo is added to the pit*	4.0×10^{-2}	1/s	estimation
F058	VesicleRadius<br>*radius of AQP2 positive vesicles*	5.0×10^{-2}	µm	[69]
F059	PitFormationRate<br>*rate at which new pits form*	5.0×10^{-2}	1/(s·µm²)	[69]
F060	EndocytosisCheckpointTime<br>*time after pit formation that determines if a pit matures*	3.0×10^{1}	s	[68]
F062	InitialConcentration<br>*of entity CLA in vesicle membrane*	2.7	µmol/l	[69]
F063	InitialConcentration<br>*of entity DYN in vesicle membrane*	2.2×10^{-1}	µmol/l	[74]
F064	InitialConcentration<br>*of entity MYO in vesicle membrane*	4.5×10^{-1}	µmol/l	[75]
F066	EndocytosisCheckpointConcentration<br>*a pit matures, if this concentration is reached*	2.9×10^{1}	µmol/l	[68]
F067	MaturationTime<br>*the average time of the endocytotic maturation process*	7.0×10^{1}	s	[68]
F069	ActinBoostVelocity<br>*the average velocity of the anterograde displacement after endocytosis*	5.0×10^{-2}	µm/s	[69]
F071	AttachmentDistance<br>*distance from the vesicle surface to the filament*	7.0×10^{-2}	µm	[100], [97]
F075	MotorMovementVelocity<br>*average velocity of dynein driven transport*	8.0×10^{-1}	µm/s	[89]
F076	MembraneDiffusivity<br>*lateral diffusivity of membrane bound entities*	4.3	µm²/s	[57], [58]
F077	ConcentrationDiffusivity<br>*cAMP diffusivity in cytoplasm*	3.2×10^{1}	µm²/s	[50]
F078	PixelDiffusivity<br>*diffusivity of macroscopic entities*	1.5×10^{-6}	µm²/s	[59], [60]
F079	InitialConcentration<br>*of entity CAMP in cytoplasm*	2.0×10^{-1}	µmol/l	[42]
F080	InitialConcentration<br>*of entity CAMP in cytoplasm*	1.0×10^{-1}	µmol/l	[42]
F081	InitialConcentration<br>*of entity CAMP in cytoplasm*	1.0×10^{-1}	µmol/l	[42]

Supplementary Table 13. **Recycling model quantitative parameters**

ID	Type	Content	Unit	Evidence
F082	InitialConcentration *of entity AKAP-P-PKAC-PKAR in vesicle membrane*	2.0×10^{-1}	µmol/l	[24]
F083	InitialConcentration *of entity AKAP-P-PKAR in vesicle membrane*	2.0	µmol/l	[24]
F084	InitialConcentration *of entity PDE in vesicle membrane*	1.5×10^{-1}	µmol/l	[42]
F085	InitialConcentration *of entity P-PDE in vesicle membrane*	1.5×10^{-1}	µmol/l	estimation
F086	InitialConcentration *of entity PP2B in vesicle membrane*	2.0×10^{-1}	µmol/l	estimation
F087	InitialConcentration *of entity AQP2 in vesicle membrane*	2.0×10^{1}	µmol/l	[28],[13]
F088	InitialConcentration *of entity AQP2-P in vesicle membrane*	9.8	µmol/l	[28],[13]
F089	InitialConcentration *of entity VAMP2 in vesicle membrane*	6.7×10^{-1}	µmol/l	[101]
F090	InitialConcentration *of entity AKAP-P-PKAC-PKAR in apical plasma membrane*	1.0×10^{-1}	µmol/l	[24]
F091	InitialConcentration *of entity AKAP-P-PKAR in apical plasma membrane*	1.0	µmol/l	[24]
F092	InitialConcentration *of entity PDE in apical plasma membrane*	5.0×10^{-2}	µmol/l	[42]
F093	InitialConcentration *of entity P-PDE in apical plasma membrane*	2.5×10^{-2}	µmol/l	estimation
F094	InitialConcentration *of entity PP2B in apical plasma membrane*	1.0×10^{-1}	µmol/l	estimation
F095	InitialConcentration *of entity AQP2-P in apical plasma membrane*	1.4×10^{1}	µmol/l	[28],[44]
F096	InitialConcentration *of entity SNAP23-STX3 in apical plasma membrane*	1.0	µmol/l	[85]
F097	InitialConcentration *of entity SRC in apical plasma membrane*	1.0×10^{-1}	µmol/l	estimation
F098	InitialConcentration *of entity P-SRC in apical plasma membrane*	1.0×10^{-1}	µmol/l	estimation
F099	InitialConcentration *of entity CSK in apical plasma membrane*	2.0×10^{-1}	µmol/l	estimation

Supplementary Table 14. **Recycling model qualitative parameters**

ID	Type	Content	Evidence
F027	Cargoes	[CAMP]	estimation
	entities that are subject to diffusion		
F029	AffectedSection	cytoplasm	estimation
	section that is affected by diffusion		
F030	Cargoes	all membrane bound entities	estimation
	entities affected by lateral membrane diffusion		
F031	AffectedRegion	apical inner plasma membrane (GO:0016324)	estimation
	region affected by lateral membrane diffusion		
F032	AppliedVesicleState	`STORAGE`	estimation
	vesicle state set, if conditions are met		
F033	ContainmentRegion	perinuclear region of cytoplasm (GO:0048471)	[13,27]
	region, where vesicles are contained during storage		
F034	BlackListVesicleStates	`[MICROTUBULE, ACTIN, EXOCYTOSIS]`	estimation
	vesicles in these states are ignored during module execution		
F035	AppliedVesicleState	`EXOCYTOSIS`	estimation
	vesicle state set, if conditions are met		
F036	Cargoes	[AQP2-P, AQP2]	[13]
	entities that regulate the departure of the vesicle from the storage region		
F037	RequiredVesicleState	`STORAGE`	estimation
	vesicle state set, if conditions are met		
F039	AttachedFilament	ACTIN	[75]
	agent that facilitates movement		
F041	AttachedMotor	MYO	[75]
	entity that facilitates movement		
F042	MotorPullDirection	+	[75]
	direction of the vesicle movement relative to the filament		
F043	AppliedVesicleState	`ACTIN`	estimation
	vesicle state set, if conditions are met		
F045	AppliedVesicleState	`TETHERED`	estimation
	vesicle state set, if conditions are met		
F046	BlackListVesicleStates	`[TETHERED, PROPELLED, FUSION]`	estimation
	vesicles in these states are ignored during module execution		
F047	ContainmentRegion	fusion (GO:0005938)	[93,80]
	region, where vesicles are tethered before fusion		
F049	MatchingQSnares	[SNAP23-STX3]	[85,84]
	required snares in membrane		
F050	MatchingRSnares	[VAMP2]	[82,83]
	required snares in vesicle		
F054	Cargoes	all entities part of the signalosome	estimation
	other cargoes		

Supplementary Table 14. **Recycling model qualitative parameters**

ID	Type	Content	Evidence
F056	ScalingEntities	[SRC, P-SRC]	estimation
	the entities that influence cargo addition rate		
F057	PrimaryCargoes	all entities in complex with AQP2	[62]
	the entities that are the relevant cargo for this vesicle		
F061	InitialConcentrations	F062, F063, F064	estimation
F065	AffectedRegion	apical inner plasma membrane (GO:0016324)	estimation
	region where endocytotic pits are able to from		
F068	BoostMediatingEntity	CLA	estimation
	the entity the determines if a vesicle is displaced and how fast		
F070	AttachedFilament	MICROTUBULE	[2]
	agent that facilitates movement		
F072	AttachedMotor	DYN	[87]
	entity that facilitates movement		
F073	MotorPullDirection	-	[87]
	direction of the vesicle movement relative to the filament		
F074	AppliedVesicleState	`MICROTUBULE`	estimation
	vesicle state set, if conditions are met		

2 Supplementary Tables

Supplementary Table 15. Aquaporin 2 concentration estimation. Literature values were used to estimate the number/concentration of AQP2 molecules in the apical and vesicle membrane in the inactive and active state of the cell.

Value	Inactive state	Active state	Unit	Evidence
total membrane permeability	0.0095	0.0226	cm/s	44
permeability of a single monomer	3.3×10^{-14}		cm^3/s	28
AQP2 monomers in the membrane	2879	6848	monomers/μm^2	
total vesicle permeability	0.03		cm/s	28
AQP2 monomers per vesicle surface	9091		monomers/μm^2	
radius vesicle	0.05		µm	
surface vesicle	0.03		μm^2	
AQP2 monomers per vesicle	650		monomers	28
nodes	15			
node scale	0.33		µm	
membrane area per node	0.11		μm^2	
total simulation area	1.66		μm^2	
AQP2 monomers in membrane	4797.98	11 414.14	monomers	
number of vesicles	7.38	17.56	vesicles	
monomers from inactive to active	6616		monomers	
fusions from inactive to active	10.18		vesicles	
total apical surface area	360		μm^2	102
fraction of simulation	216		times	
total AQP2 in membrane	1.04×10^6	2.47×10^6	monomers	
total number of vesicles	1594.41	3793.01	vesicles	
monomers from inactive to active	1.43×10^6		monomers	
vesicles from inactive to active	2198.60		vesicles	
**Concentration in membrane node**				
volume per node	0.0370		μm^3	
[AQP2] node	5.31×10^{-22}	1.26×10^{-21}	mol	
[AQP2] node	14.34	34.12	µmol/l	
membrane proteins per surface	50.000		1/μm^2	103
fraction of aquaporin of total	5.76	13.70	%	
**Concentration in vesicle**				
[AQP2] vesicle	1.08×10^{-21}		mol	
[AQP2] vesicle	29.14		µmol/l	
fraction of aquaporin of total		41.38	%	
[AQP2-P] fold increase	2.00			5
phosphorylation ratio (AQP2-P/AQP2)	0.46	1.70		13
[AQP2] vesicle	19.96	10.78	µmol/l	
[AQP2-P] vesicle	9.18	18.36	µmol/l	

Supplementary Table 16. Description of the modification operations and their applied implicit conditions The *entities* row describes whether candidates are evaluated during network generation or if predefined entities are used. The *conditions* row describes implicit filter criteria that are used to determine if an entity is a candidate. The *modifications* row describes the creation and modification of the candidate graphs to get to the product graphs.

BIND

entities	primary	secondary
conditions	has primary entity	secondary entity
	has unoccupied binding site for secondary	has unoccupied binding site for primary
modifications	create new graph from copy of first and second candidate	
	determine nodes with unoccupied binding sites in both candidates	
	add edge between previously determined nodes	

ADD

entities	primary	static
conditions	has pr<imary entity	predefined
	has unoccupied binding site for static	
modifications	create new graph from copy of first and static candidate	
	determine nodes with unoccupied binding sites in both candidates	
	add edge between previously determined nodes	

RELEASE

entities	primary	static
conditions	has primary entity	predefined
	has secondary entity	
	has occupied binding site for static	
modifications	create two new graphs from copy of candidate	
	remove edge for binding site	
	from one graph remove subgraph including the primary entity	
	from one graph remove subgraph including the secondary entity	

REMOVE

entities	primary	static
conditions	has primary entity	predefined
	has secondary entity	
	has occupied binding site for static	
modifications	create new graph from copy of candidate	
	remove edge for binding site	
	remove nodes that are in one subgraph with the static entity	

3 Supplementary Figures

Supplementary Figure 1: Model Overview The next page contains an overview of the processes and reactions in SBGN notation (Process Description). Microscale and reaction based processes and entities are colored blue, whereas macroscopic and agent based processes and entities are colored green. Chemical entities are annotated with a state that indicates whether the entity is membrane-bound (m) or in the cytoplasm (c). Phosphorylation of chemical entities is annotated with a lowercase "p". Agent-based entities are annotated with the agent state. The two binding sites of PKAR are annotated with A and B. Movement of chemical entities or vesicles is indicated as a perturbing agent (depicted by a modified hexagonal shape having two opposite concave face). The direction of the movement is annotated to the agent. Compartments are indicated with large rectangles with rounded corners. Contextually, entities and processes are grouped to their respective context in the AQP2 recycling pathway.

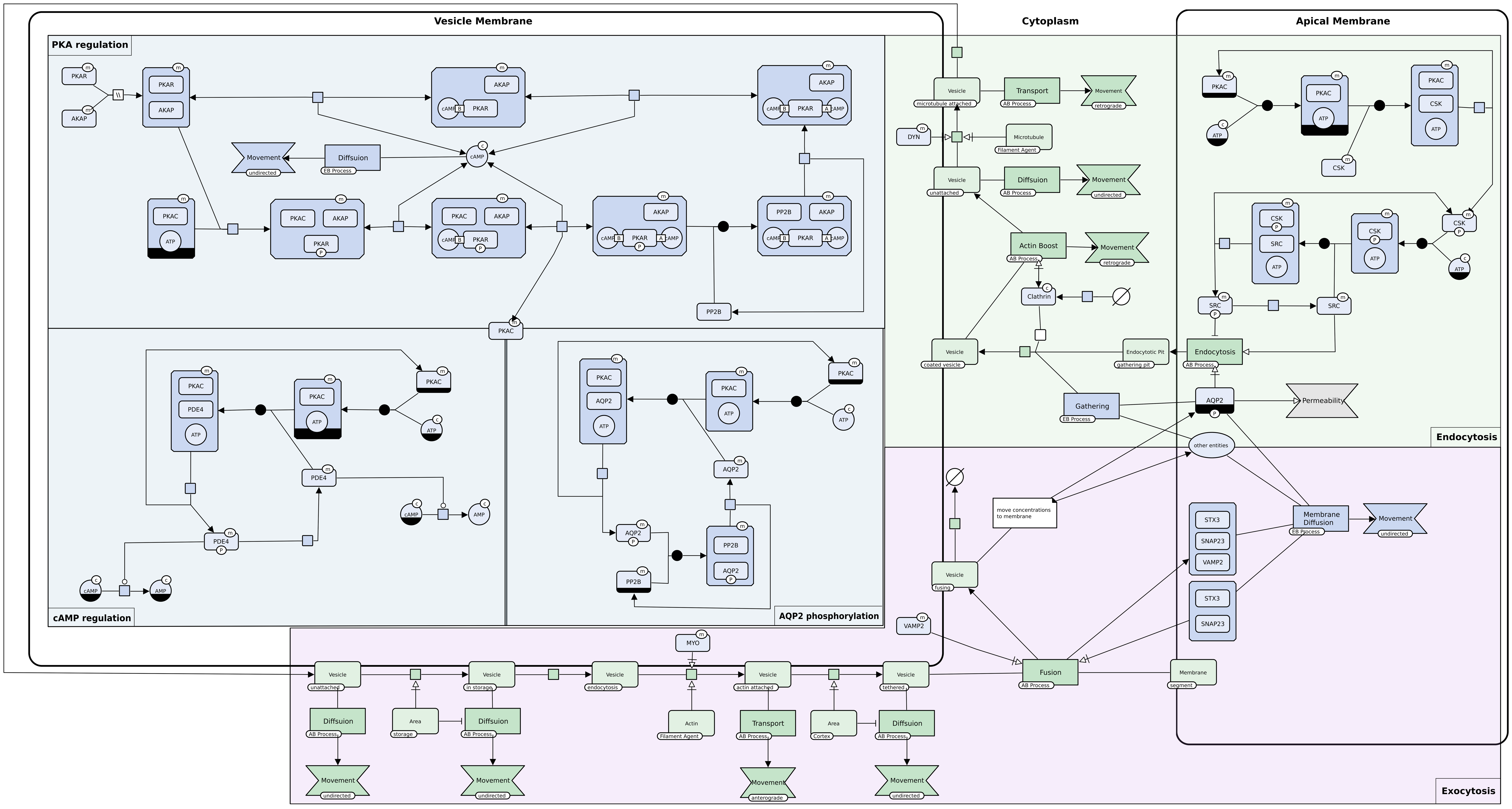

Vesicle Membrane
Cytoplasm
Apical Membrane
PKA regulation
cAMP regulation
AQP2 phosphorylation
Endocytosis
Exocytosis
PKAR
AKAP
PKAC
ATP
cAMP
PP2B
Movement
undirected
Diffsuion
EB Process
PDE4
AMP
AQP2
Vesicle
microtubule attached
Transport
AB Process
retrograde
DYN
Microtubule
Filament Agent
unattached
Actin Boost
Clathrin
coated vesicle
Endocytotic Pit
gathering pit
Gathering
CSK
SRC
Endocytosis
Permeability
other entities
move concentrations to membrane
STX3
SNAP23
VAMP2
Membrane Diffusion
fusing
Fusion
Membrane
segment
MYO
in storage
endocytosis
actin attached
tethered
Area
storage
Cortex
Actin
anterograde

4 Supplementary Algorithms

Supplementary Algorithm 1: Reaction network generation. Process reaction chains that are generated from reaction rules. Assigns possible binding sites to complex entities. Furthermore, generates possible individual reactions from reaction chains. Repeats until no new reactions are generated.

```
Input: All reaction chains ℂ, All initial simple chemical entities E
▷ determine possible binding sites
B : E ↦ S∗, a mapping for each chemical entity e to a set of binding sites S ∈ S∗
foreach reaction chain C in the set of reaction chains ℂ do
    foreach basic reactor c in the reaction chains C do
        if c involves binding site s in conjunction with e then
            add s to B for entity e

▷ create initial chemical entities
foreach entity e in the set entites E do
    S ← the set of binding sites associated to e from B
    G ← a new graph representing the complex entity e
    add e as a node to G
    foreach binding site s in the set binding sites S do
        add s to e

▷ generate reactions
stable ← false
repeat
    n_p ← previous number of reactions
    ▷ process each reaction chain
    foreach reaction chain C in the set of reaction chains ℂ do
        T ← a new, empty set of reaction stacks
        foreach basic reactor c in the reaction chains C do
            collect candidate entites that pass all filters
            apply the specified reaction
            foreach possible reaction r, consisting of substrates s and products p do
                if no track t ∈ T has top most element equal to s then
                    create a new track t
                    push s to track t
                    push p to track t
                else
                    push p to track t
            candidate entities ← all distinct products from all reactions
    n_c ← current number of reactions
    if n_p = n_c then
        stable ← true
until stable
```

Supplementary Algorithm 2: Collision detection and position updates. Sphere-like agents are moved if they do not collide with other sphere-like agents, membrane segments, or the simulation border.

Input: all sphere-like agents A, all membranes M, the simulation extend L_x and L_y

foreach *sphere-like agent a* **in** *the set of sphere-like agents A* **do**
  ▷ determine positions for t_{n+1}
  $p_a^{n+1} = p_a^n + \sum_{m \in \mathbb{M}} \Delta p_a^{\Delta t}$

foreach *sphere-like agent* a_1 **in** *the set of sphere-like agents V* **do**
  ▷ check collision with other sphere-like agents
  $r_1 \leftarrow$ radius r of a_1
  foreach *sphere-like agent* a_2 **in** *the set of sphere-like agents A* **do**
    if $a_1 \neq a_2$ **then**
      $r_2 \leftarrow$ radius r of a_2
      $d \leftarrow d_R(p_{a_1}^{n+1}, p_{a_2}^{n+1})$ ▷ distance between both agents
      if $d < r_1 + r_2$ **then**
        ▷ radii of both agents intersect
        $p_{a_1}^{n+1} \leftarrow p_{a_1}^n$
        break

  ▷ check collision with membrane segments m
  foreach *membrane segment m* **in** *in membrane M* **do**
    $l_a \leftarrow p_{a_1}^{n+1} - p_{a_1}^n$ ▷ line segment between p_{a-1}^{n+1} and $p_{a_1}^n$
    $l_m \leftarrow m_{end} - m_{start}$ ▷ membrane segment between points m_{start} and m_{end}
    $i_a \leftarrow (m_{start} - p_t^{a_1}) \times \frac{l_m}{l_a \times l_m}$
    $i_m \leftarrow (p_t^{a_1} - m_{start}) \times \frac{l_a}{l_a \times l_m}$
    if $(l_m \times l_a \neq 0) \wedge (0 \leq i_a \leq 1) \wedge (0 \leq i_m \leq 1)$ **then**
      ▷ constructed line segments intersect
      $p_{a_1}^{n+1} \leftarrow p_{a_1}^n$
      break

  ▷ check collision with simulation border
  $x_p \leftarrow p_{a_1}^{n+1} x$
  $y_p \leftarrow p_{a_1}^{n+1} y$
  if $(0 \leq x_p \leq L_x) \wedge (0 \leq y_p \leq L_y)$ **then**
    ▷ no collisions detected, keep new position
  else
    ▷ outside of the simulation box
    $p_{a_1}^{n+1} \leftarrow p_{a_1}^n$

Supplementary Algorithm 3: Vesicle indexing and surface area assignment. Determines whether a sphere intersects with the spatial representation of a grid point and calculates the surface area that is considered inside each box.

Input: all vesicles V, All grid points Ω

foreach *vesicle* v **in** *the set of vesicles* V **do**
  foreach *grid point* (x,y) **in** *set of grid points* Ω **do**
    $R \leftarrow (x-\frac{\Delta s}{2}, y-\frac{\Delta s}{2}), (x-\frac{\Delta s}{2}, y+\frac{\Delta s}{2}), (x+\frac{\Delta s}{2}, y+\frac{\Delta s}{2}), (x+\frac{\Delta s}{2}, y-\frac{\Delta s}{2})$
    if *vesicle position* p_v **is inside** R **then**
      foreach *vertex* q **in** *rectangle* R **do**
        calculate distance $d(p,q)$ between vesicle v and vertex q
        if *distance* $d(p,q) <$ *vesicle radius* r_v **then**
          ▷ `the sphere intersects four polygons`
          calculate projection $p(q,v)$ of q onto sphere surface of v
          determine vertical intersections $q_N, q_S \leftarrow (q_x, p(q,v)_y, 0)$
          determine horizontal intersections $q_E, q_W \leftarrow (p(q,v)_x, q_y, 0)$
          determine top intersection $q_T \leftarrow (q_x, q_y, p(q,v)_z)$
          setup spherical triangle T_{NE} for q_N, q_E, q_T
          setup spherical triangle T_{NW} for q_N, q_W, q_T
          setup spherical triangle T_{SE} for q_S, q_E, q_T
          setup spherical triangle T_{SW} for q_S, q_W, q_T
          calculate surfaces of the spherical trianges
          associate surface areas with respective grid points to v
          continue with next vesicle
      ▷ `(else) check if` *c* `intersects with exactly two regions`
      calculate total surface of sphere s_v
      foreach *edge* e **in** R **do**
        calculate intersections I between vesicle v and edge e
        if $|I| = 2$ **then**
          calculate height h of the sphere cap defined by $i_1, i_2 \in I$
          calculate surface s_{cap} of the sphere cap
          associate surface area $s_v - s_{cap}$ to current grid point
          associate surface area s_{cap} to neighboring grid point
          continue with next vesicle
      ▷ `(else) vesicle is fully contained`
      associate surface area s_v to current grid point

Supplementary Algorithm 4: Clathrin-mediated endocytosis. Random pits are initiated at membrane segments. If threshold concentrations are reached, vesicle undergo a maturation phase before movable vesicles are created in their place.

$P_a^{\times}$ pits that are marked for assembly
P_a pits that currently assembling
$P_m^{\times}$ pits that are marked for maturation
P_m pits that currently maturing
$P_c^{\times}$ pits that are marked for cancellation
$P_d^{\times}$ pits that are turned into departing vesicles
▷ `Determine if new` *`aspiring pits`* `will form during this time interval.`
foreach *membrane segment m* **in** *in membrane M* **do**
    $l_m \leftarrow (x_i, y_i), (x_{i+1}, y_{i+1})$ ▷ `the line segment associated to` m
    $a_m \leftarrow \|l_m\| \cdot \Delta s$ ▷ `the mebrane area assigned to` m
    $P(m) = k_p \cdot a_m \cdot \Delta t$
    ξ is an evenly distributed random value between 0 and 1
    if $\xi \leq P(m)$ **then**
        create new pit p
        assign random spawn site on membrane segment m to pit p
        assign pit radius to pit p
        assign checkpoint time $t + t_c$ to pit p
        move pit to $P_a^{\times}$
▷ `check checkpoints for assembling pits`
foreach *pit* p_a **in** P_a **do**
    ▷ `sum concentrations of relevant cargo`
    if *cargo concentration of* $p_a \geq$ *threshod concentration* **then**
        move p_a to $P_m^{\times}$
    if *checkpoint time of* $p_a \leq$ *current simulation time* **then**
        move p_a to $P_c^{\times}$
▷ `check if maturing pits are ready for departure`
foreach *pit* p_m **in** P_m **do**
    if *target maturation time* $\leq$ *current simulation time* **then**
        move p_m to $P_d^{\times}$
▷ `apply changes`
if *timestep was accepted* **then**
    foreach *pit* p_a **in** $P_a^{\times}$ **do**
        move p_a to P_a
    foreach *pit* p_m **in** $P_m^{\times}$ **do**
        move p_m to P_m
    foreach *pit* p_d **in** $P_d^{\times}$ **do**
        create vesicle at pit spawn site
        move concentrations from pit to vesicle
    clear $P_c^{\times}$
else
    clear $P_a^{\times}$, clear $P_m^{\times}$, clear $P_d^{\times}$
    foreach *pit* p_d **in** $P_d^{\times}$ **do**
        move p_d back to P_m
    foreach *pit* p_c **in** $P_c^{\times}$ **do**
        move p_c back to P_m

Supplementary Algorithm 5: Vesicle fusion. If a vesicle comes close to a membrane segment, availability of the correct SNARE molecules is determined. After the vesicle is tethered to the membrane for a certain amount of time, the vesicle is removed and chemical entities move to the target membrane segment.

$V_t^{\times}$ vesicles that are marked for tethering
V_t vesicles that are currently tethered
$V_f^{\times}$ vesicles that are marked for fusion
▷ check targeted fusion times
foreach *vesicle* v_t **in** V_t **do**
    if *target fusion time* $\leq$ *current simulation time* **then**
        move v_t to $V_f^{\times}$
▷ check whether new vesicle can start fusion
foreach *vesicle* v **in** V **do**
    if *vesicle is not in an fusion ready state* **then**
        continue with next vesicle
    $M \leftarrow$ membrane segments referenced to associated nodes of v
    foreach *membrane segment* m **in** M **do**
        ▷ check distance
        $d_{mv} \leftarrow$ distance between membrane segment m and vesicle v
        if $d_{mv} \leq d_a$ **then**
            $n_m \leftarrow$ number of molecules of Q-SNARES in the target membrane
            $n_v \leftarrow$ number of molecules of R-SNAES in the vesicle membrane
            if $n_m \geq n_p$ *and* $n_v \geq n_p$ **then**
                move v to $V_t^{\times}$
                continue with next vesicle
▷ apply changes
if *timestep was accepted* **then**
    foreach *vesicle* v_f **in** $V_f^{\times}$ **do**
        add all chemical enitites in vesicle membrane to target membrane
        add all cargo molecules to compartment opposite of the membrane
    clear $V_f^{\times}$
    foreach *vesicle* v_t **in** $V_t^{\times}$ **do**
        determine targeted fusion time of v_h by adding t_f to current time
        add n_p complexes of Q- and R-SNARES to target membrane
        remove n_p entities of Q- and R-SNARES from vesicle membrane
        move v_h to V_t

Supplementary Algorithm 6: Sphere-like agent movement along line-like agents defined by a target direction.

Input: An sphere-like agent SLA attachted to a line-like agent LLA
Output: The displacement $\Delta p_{\text{SLA}}^{\Delta t}$ of SLA

$p_{\text{LLA}}^{i} \leftarrow$ the position of the line-like agent's segment i the vesicle is attached to
$p_{\text{SLA}} \leftarrow$ the position of the sphere-like agent
if *target direction = MINUS* **then**
  if $p_{LLA}^{i} = p_{LLA}^{0}$ **then**
    ▷ `this is the first segment`
    return $\Delta p_{\text{SLA}}^{\Delta t} \leftarrow (0,0)$
  else
    $\hat{u} \leftarrow \frac{p_{\text{LLA}}^{i-1} - p_{\text{SLA}}}{\|p_{\text{LLA}}^{i-1} - p_{\text{SLA}}\|}$
else
  if $p_{LLA}^{i} = p_{LLA}^{n}$ **then**
    ▷ `this is the last segment`
    return $\Delta p_{\text{SLA}}^{\Delta t} \leftarrow (0,0)$
  else
    $\hat{u} \leftarrow \frac{p_{\text{LLA}}^{i+1} - p_{\text{SLA}}}{\|p_{\text{LLA}}^{i+1} - p_{\text{SLA}}\|}$
return $\Delta p_{\text{SLA}}^{\Delta t} \leftarrow v_m \cdot \hat{u}$

References

1. Tajika, Y. *et al.* Aquaporin-2 is retrieved to the apical storage compartment via early endosomes and phosphatidylinositol 3-kinase-dependent pathway. *Endocrinology* **145**, 4375–4383, DOI: 10.1210/en.2004-0073 (2004). Initial vesicle positioning.
2. Vossenkämper, A. *et al.* Microtubules are needed for the perinuclear positioning of aquaporin-2 after its endocytic retrieval in renal principal cells. *Am. J. Physiol. Physiol.* **293**, C1129–C1138, DOI: 10.1152/ajpcell.00628.2006 (2007). Initial vesicle positioning microtubules for transport after endocytosis.
3. Takata, K., Matsuzaki, T., Tajika, Y., Ablimit, A. & Hasegawa, T. Localization and trafficking of aquaporin 2 in the kidney. *Histochem. cell biology* **130**, 197–209, DOI: 10.1007/s00418-008-0457-0 (2008). Initial vesicle positioning.
4. Fushimi, K., Sasaki, S. & Marumo, F. Phosphorylation of serine 256 is required for cAMP-dependent regulatory exocytosis of the aquaporin-2 water channel. *J. Biol. Chem.* **272**, 14800–14804, DOI: 10.1074/jbc.272.23.14800 (1997).
5. Hoffert, J. D. *et al.* Dynamics of aquaporin-2 serine-261 phosphorylation in response to short-term vasopressin treatment in collecting duct. *Am. J. Physiol. Physiol.* **292**, F691–F700, DOI: 10.1152/ajprenal.00284.2006 (2007).
6. Moeller, H. B., Knepper, M. A. & Fenton, R. A. Serine 269 phosphorylated aquaporin-2 is targeted to the apical membrane of collecting duct principal cells. *Kidney Int.* **75**, 295–303, DOI: 10.1038/ki.2008.505 (2009).
7. Katsura, T., Gustafson, C. E., Ausiello, D. A. & Brown, D. Protein kinase a phosphorylation is involved in regulated exocytosis of aquaporin-2 in transfected LLC-PK1 cells. *Am. J. Physiol. Physiol.* **272**, F816–F822, DOI: 10.1152/ajprenal.1997.272.6.f816 (1997).
8. Brown, D. The ins and outs of aquaporin-2 trafficking. *Am. J. Physiol. Physiol.* **284**, F893–F901, DOI: 10.1152/ajprenal.00387.2002 (2003).
9. Noda, Y. & Sasaki, S. Regulation of aquaporin-2 trafficking and its binding protein complex. *Biochimica et Biophys. Acta (BBA) - Biomembr.* **1758**, 1117–1125, DOI: 10.1016/j.bbamem.2006.03.004 (2006).
10. Donovan, K. W. & Bretscher, A. Myosin-v is activated by binding secretory cargo and released in coordination with rab/exocyst function. *Dev. Cell* **23**, 769–781, DOI: 10.1016/j.devcel.2012.09.001 (2012).
11. Chou, C.-L. *et al.* Regulation of aquaporin-2 trafficking by vasopressin in the renal collecting duct roles of ryanodine-sensitive ca2+ stores and calmodulin. *J. Biol. Chem.* **275**, 36839–36846, DOI: 10.1074/jbc.m005552200 (2000).
12. Yip, K.-P. Coupling of vasopressin-induced intracellular ca2+ mobilization and apical exocytosis in perfused rat kidney collecting duct. *The J. Physiol.* **538**, 891–899, DOI: 10.1113/jphysiol.2001.012606 (2002).

13. Kamsteeg, E., Heijnen, I., van Os, C. & Deen, P. The subcellular localization of an aquaporin-2 tetramer depends on the stoichiometry of phosphorylated and nonphosphorylated monomers. *The J. Cell Biol.* **151**, 919–930, DOI: 10.1083/jcb.151.4.919 (2000). Ratio of aqp2 phosphorylation.

14. Klussmann, E. & Rosenthal, W. Role and identification of protein kinase a anchoring proteins in vasopressin-mediated aquaporin-2 translocation. *Kidney Int.* **60**, 446–449, DOI: 10.1046/j.1523-1755.2001.060002446.x (2001).

15. Henn, V. *et al.* Identification of a novel a-kinase anchoring protein 18 isoform and evidence for its role in the vasopressin-induced aquaporin-2 shuttle in renal principal cells. *J. Biol. Chem.* **279**, 26654–26665, DOI: 10.1074/jbc.m312835200 (2004). Akap18d in renal principal cells akap18 is on the same vesicles as aqp2 and pka akap18d also travels to the membrane upon avp signal.

16. Horner, A., Goetz, F., Tampé, R., Klussmann, E. & Pohl, P. Mechanism for targeting the a-kinase anchoring protein AKAP18δ to the membrane. *J. Biol. Chem.* **287**, 42495–42501, DOI: 10.1074/jbc.m112.414946 (2012). Membrane association of akap18 despite having no tm anchor.

17. McSorley, T. *et al.* Spatial organisation of AKAP18 and PDE4 isoforms in renal collecting duct principal cells. *Eur. J. Cell Biol.* **85**, 673–678, DOI: 10.1016/j.ejcb.2006.01.005 (2006). Akap18d binds to pde4 connection to aqp2 in collectiong duct.

18. Jo, I. *et al.* AQP2 is a substrate for endogenous PP2b activity within an inner medullary AKAP-signaling complex. *Am. J. Physiol. Physiol.* **281**, F958–F965, DOI: 10.1152/ajprenal.2001.281.5.f958 (2001).

19. Smith, F. D. & Scott, J. D. Protein kinase a activation: Something new under the sun? *The J. Cell Biol.* **217**, 1895–1897, DOI: 10.1083/jcb.201805011 (2018).

20. Zhang, P. *et al.* Single turnover autophosphorylation cycle of the PKA RIIβ holoenzyme. *PLOS Biol.* **13**, e1002192, DOI: 10.1371/journal.pbio.1002192 (2015). PKAR2b binds PKAC-ATP PKAC-ATP phosphorylates PKAR2b, but PKAC is not released PKAC is only relaes upon second CAMP binding kinetics for PKAC binding with phospho- and nonphospho forms of PKAR2b.

21. Øgreid, D. & Døskeland, S. O. The kinetics of association of cyclic AMP to the two types of binding sites associated with protein kinase II from bovine myocardium. *FEBS Lett.* **129**, 287–292, DOI: 10.1016/0014-5793(81)80185-8 (1981). PKAR2 CAMP binding sites A and B have similar association rates.

22. Øgreid, D. & Døskeland, S. O. Activation of protein kinase isoenzymes under near physiological conditions. *FEBS Lett.* **150**, 161–166, DOI: 10.1016/0014-5793(82)81326-4 (1982). PKAR2 CAMP binding sites A and B have different dissociation rate constants kd site A is only phosphorylated when site b is occupied.

23. Zhang, P. *et al.* Structure and allostery of the PKA RII tetrameric holoenzyme. *Science* **335**, 712–716, DOI: 10.1126/science.1213979 (2012). Differences in CAMP binding kineitcs in PKAR2b in tetrameic composition allostery kinetics pathway of PKAC activation PKAR2b is phosphorylated in basal state.

24. Walker-Gray, R., Stengel, F. & Gold, M. G. Mechanisms for restraining cAMP-dependent protein kinase revealed by subunit quantitation and cross-linking approaches. *Proc. Natl. Acad. Sci.* **114**, 10414–10419, DOI: 10.1073/pnas.1701782114 (2017). Concentrations of PKAC and PKAR in different cell types PKAC is tethered to the membrane via myristylation.

25. Blumenthal, D. K., Takio, K., Hansen, R. S. & Krebs, E. G. Dephosphorylation of camp-dependent protein kinase regulatory subunit (type ii) by calmodulin-dependent protein phosphatase. determinants of substrate specificity. *J. Biol. Chem.* **261**, 8140–8145 (1986). PP2B dephosphorylates PKAR2 at a similar rate to other good substrates.

26. Moeller, H. B., Praetorius, J., Rutzler, M. R. & Fenton, R. A. Phosphorylation of aquaporin-2 regulates its endocytosis and protein-protein interactions. *Proc. Natl. Acad. Sci.* **107**, 424–429, DOI: 10.1073/pnas.0910683107 (2009).

27. Nejsum, L. N., Zelenina, M., Aperia, A., Frøkiær, J. & Nielsen, S. Bidirectional regulation of AQP2 trafficking and recycling: involvement of AQP2-s256 phosphorylation. *Am. J. Physiol. Physiol.* **288**, F930–F938, DOI: 10.1152/ajprenal.00291.2004 (2005).

28. Yang, B. & Verkman, A. Water and glycerol permeabilities of aquaporins 1–5 and mip determined quantitatively by expression of epitope-tagged constructs in xenopus oocytes. *J. Biol. Chem.* **272**, 16140–16146, DOI: 10.1074/jbc.272.26.16140 (1997).

29. Lindskog, M., Kim, M., Wikström, M. A., Blackwell, K. T. & Kotaleski, J. H. Transient calcium and dopamine increase PKA activity and DARPP-32 phosphorylation. *PLoS Comput. Biol.* **2**, e119, DOI: 10.1371/journal.pcbi.0020119 (2006).

30. Zhang, P. *et al.* An isoform-specific myristylation switch targets type II PKA holoenzymes to membranes. *Structure* **23**, 1563–1572, DOI: 10.1016/j.str.2015.07.007 (2015). PKAC is tethered to the membrane via myristylation PKAR2 is tethered to the membrane via myristylation, but not PKAR1.

31. Houslay, M. D. & Adams, D. R. PDE4 cAMP phosphodiesterases: modular enzymes that orchestrate signalling cross-talk, desensitization and compartmentalization. *Biochem. J.* **370**, 1–18, DOI: 10.1042/bj20021698 (2003).

32. Cheng, X., Phelps, C. & Taylor, S. S. Differential binding of cAMP-dependent protein kinase regulatory subunit isoforms iα and IIβ to the catalytic subunit. *J. Biol. Chem.* **276**, 4102–4108, DOI: 10.1074/jbc.m006447200 (2000). Binding kinetics PKAR2b and PKAC ka and kd.

33. Shaffer, J. & Adams, J. A. An ATP-linked structural change in protein kinase a precedes phosphoryl transfer under physiological magnesium concentrations†. *Biochemistry* **38**, 5572–5581, DOI: 10.1021/bi982768q (1999).

34. Sette, C. & Conti, M. Phosphorylation and activation of a cAMP-specific phosphodiesterase by the cAMP-dependent protein kinase. *J. Biol. Chem.* **271**, 16526–16534, DOI: 10.1074/jbc.271.28.16526 (1996).

35. Zimmermann, B., Schweinsberg, S., Drewianka, S. & Herberg, F. W. Effect of metal ions on high-affinity binding of pseudosubstrate inhibitors to PKA. *Biochem. J.* **413**, 93–101, DOI: 10.1042/bj20071665 (2008). Pseudosubstrate binding pka ka and kd.

36. Manschwetus, J. T. *et al.* A stapled peptide mimic of the pseudosubstrate inhibitor PKI inhibits protein kinase a. *Molecules* **24**, 1567, DOI: 10.3390/molecules24081567 (2019). Pseudosubstrate binding pka ka and kd.

37. Stefan, E. *et al.* Compartmentalization of cAMP-dependent signaling by phosphodiesterase-4d is involved in the regulation of vasopressin-mediated water reabsorption in renal principal cells. *J. Am. Soc. Nephrol.* **18**, 199–212, DOI: 10.1681/asn.2006020132 (2006).

38. Taskén, K. A. *et al.* Phosphodiesterase 4d and protein kinase a type II constitute a signaling unit in the centrosomal area. *J. Biol. Chem.* **276**, 21999–22002, DOI: 10.1074/jbc.c000911200 (2001).

39. Shacter, E., Chock, P. B. & Stadtman, E. Regulation through phosphorylation/dephosphorylation cascade systems. *J. Biol. Chem.* **259**, 12252–12259 (1984).

40. Buxbaum, J. D. & Dudai, Y. A quantitative model for the kinetics of camp-dependent protein kinase (type ii) activity. long-term activation of the kinase and its possible relevance to learning and memory. *J. Biol. Chem.* **264**, 9344–9351 (1989). Lots of kinetic data for pka phosphorylation, camp binding, subunit binding, and dephosphorylation.

41. Xiang, B. *et al.* The catalytically active domain in the a subunit of calcineurin. *Biol. Chem.* **384**, DOI: 10.1515/bc.2003.158 (2003).

42. Koschinski, A. & Zaccolo, M. Activation of PKA in cell requires higher concentration of cAMP than in vitro: implications for compartmentalization of cAMP signalling. *Sci. Reports* **7**, DOI: 10.1038/s41598-017-13021-y (2017).

43. Feinstein, W. P., Zhu, B., Leavesley, S. J., Sayner, S. L. & Rich, T. C. Assessment of cellular mechanisms contributing to cAMP compartmentalization in pulmonary microvascular endothelial cells. *Am. J. Physiol. Physiol.* **302**, C839–C852, DOI: 10.1152/ajpcell.00361.2011 (2012).

44. Deen, P. *et al.* Aquaporin-2 transfection of madin-darby canine kidney cells reconstitutes vasopressin-regulated transcellular osmotic water transport. *J. Am. Soc. Nephrol.* **8**, 1493–1501 (1997).

45. Beavo, J. A. & Brunton, L. L. Cyclic nucleotide research — still expanding after half a century. *Nat. Rev. Mol. Cell Biol.* **3**, 710–717, DOI: 10.1038/nrm911 (2002).

46. Brunton, L., Hayes, J. & Mayer, S. Functional compartmentation of cyclic amp and protein kinase in heart. *Adv. cyclic nucleotide research* **14**, 391–397 (1981).

47. Iancu, R. V., Jones, S. W. & Harvey, R. D. Compartmentation of cAMP signaling in cardiac myocytes: A computational study. *Biophys. J.* **92**, 3317–3331, DOI: 10.1529/biophysj.106.095356 (2007).

48. Tasken, K. & Aandahl, E. M. Localized effects of camp mediated by distinct routes of protein kinase a. *Physiol. reviews* **84**, 137–167, DOI: 10.1152/physrev.00021.2003 (2004).

49. Conti, M., Mika, D. & Richter, W. Cyclic AMP compartments and signaling specificity: Role of cyclic nucleotide phosphodiesterases. *The J. Gen. Physiol.* **143**, 29–38, DOI: 10.1085/jgp.201311083 (2013).

50. Richards, M. *et al.* Intracellular tortuosity underlies slow cAMP diffusion in adult ventricular myocytes. *Cardiovasc. Res.* **110**, 395–407, DOI: 10.1093/cvr/cvw080 (2016).

51. Xin, W. *et al.* Estimating the magnitude of near-membrane PDE4 activity in living cells. *Am. J. Physiol. Physiol.* **309**, C415–C424, DOI: 10.1152/ajpcell.00090.2015 (2015).

52. Agarwal, S. R., Clancy, C. E. & Harvey, R. D. Mechanisms restricting diffusion of intracellular cAMP. *Sci. Reports* **6**, DOI: 10.1038/srep19577 (2016).

53. Yang, P.-C. *et al.* A computational modeling and simulation approach to investigate mechanisms of subcellular cAMP compartmentation. *PLOS Comput. Biol.* **12**, e1005005, DOI: 10.1371/journal.pcbi.1005005 (2016).

54. Chen, W., Levine, H. & Rappel, W.-J. Compartmentalization of second messengers in neurons: A mathematical analysis. *Phys. Rev. E* **80**, DOI: 10.1103/physreve.80.041901 (2009).

55. Saucerman, J. J., Greenwald, E. C. & Polanowska-Grabowska, R. Mechanisms of cyclic AMP compartmentation revealed by computational models. *The J. Gen. Physiol.* **143**, 39–48, DOI: 10.1085/jgp.201311044 (2013).

56. Torres-Quesada, O., Mayrhofer, J. E. & Stefan, E. The many faces of compartmentalized PKA signalosomes. *Cell. Signal.* **37**, 1–11, DOI: 10.1016/j.cellsig.2017.05.012 (2017).

57. Saffman, P. G. & Delbruck, M. Brownian motion in biological membranes. *Proc. Natl. Acad. Sci.* **72**, 3111–3113, DOI: 10.1073/pnas.72.8.3111 (1975).

58. Ramadurai, S. *et al.* Lateral diffusion of membrane proteins. *J. Am. Chem. Soc.* **131**, 12650–12656, DOI: 10.1021/ja902853g (2009).

59. Rothman, J. S., Kocsis, L., Herzog, E., Nusser, Z. & Silver, R. A. Physical determinants of vesicle mobility and supply at a central synapse. *eLife* **5**, DOI: 10.7554/elife.15133 (2016).

60. Holt, M., Cooke, A., Neef, A. & Lagnado, L. High mobility of vesicles supports continuous exocytosis at a ribbon synapse. *Curr. Biol.* **14**, 173–183, DOI: 10.1016/j.cub.2003.12.053 (2004).

61. Sun, T.-X. *et al.* Aquaporin-2 localization in clathrin-coated pits: inhibition of endocytosis by dominant-negative dynamin. *Am. J. Physiol. Physiol.* **282**, F998–F1011, DOI: 10.1152/ajprenal.00257.2001 (2002).

62. Wang, P.-J. *et al.* Vasopressin-induced serine 269 phosphorylation reduces sipa1l1 (signal-induced proliferation-associated 1 like 1)-mediated aquaporin-2 endocytosis. *J. Biol. Chem.* **292**, 7984–7993, DOI: 10.1074/jbc.m117.779611 (2017).

63. Shimizu, K. *et al.* Phosphorylation and dephosphorylation of aquaporin-2 at serine 269 and its subcellular distribution during vasopressin-induced exocytosis and subsequent endocytosis in the rat kidney. *Arch. Histol. Cytol.* **77**, 25–38, DOI: 10.1679/aohc.77.25 (2017).

64. Hoffert, J. D. *et al.* Vasopressin-stimulated increase in phosphorylation at ser269 potentiates plasma membrane retention of aquaporin-2. *J. Biol. Chem.* **283**, 24617–24627, DOI: 10.1074/jbc.m803074200 (2008).

65. Cheung, P. W., Terlouw, A., Janssen, S. A., Brown, D. & Bouley, R. Inhibition of non-receptor tyrosine kinase src induces phosphoserine 256-independent aquaporin-2 membrane accumulation. *The J. Physiol.* DOI: 10.1113/jp277024 (2018).

66. Okada, M. & Nakagawa, H. A protein tyrosine kinase involved in regulation of pp60c-src function. *J. Biol. Chem.* **264**, 20886–20893 (1989).

67. Roskoski, R. Src kinase regulation by phosphorylation and dephosphorylation. *Biochem. Biophys. Res. Commun.* **331**, 1–14, DOI: 10.1016/j.bbrc.2005.03.012 (2005).

68. Loerke, D. *et al.* Cargo and dynamin regulate clathrin-coated pit maturation. *PLoS Biol.* **7**, e1000057, DOI: 10.1371/journal.pbio.1000057 (2009).

69. Ehrlich, M. *et al.* Endocytosis by random initiation and stabilization of clathrin-coated pits. *Cell* **118**, 591–605, DOI: 10.1016/j.cell.2004.08.017 (2004).

70. Yaqub, S. *et al.* Activation of c-terminal src kinase (csk) by phosphorylation at serine-364 depends on the csk-src homology 3 domain. *Biochem. J.* **372**, 271–278, DOI: 10.1042/bj20030021 (2003).

71. Roskoski, R. Src protein–tyrosine kinase structure and regulation. *Biochem. Biophys. Res. Commun.* **324**, 1155–1164, DOI: 10.1016/j.bbrc.2004.09.171 (2004).

72. Lieser, S. A., Shaffer, J. & Adams, J. A. Src tail phosphorylation is limited by structural changes in the regulatory tyrosine kinase csk. *J. Biol. Chem.* **281**, 38004–38012, DOI: 10.1074/jbc.m607824200 (2006).

73. Merrifield, C. J., Feldman, M. E., Wan, L. & Almers, W. Imaging actin and dynamin recruitment during invagination of single clathrin-coated pits. *Nat. Cell Biol.* **4**, 691–698, DOI: 10.1038/ncb837 (2002).

74. Hendricks, A. G. *et al.* Motor coordination via a tug-of-war mechanism drives bidirectional vesicle transport. *Curr. Biol.* **20**, 697–702, DOI: 10.1016/j.cub.2010.02.058 (2010).

75. Nedvetsky, P. I. *et al.* A role of myosin vb and rab11-FIP2 in the aquaporin-2 shuttle. *Traffic* **8**, 110–123, DOI: 10.1111/j.1600-0854.2006.00508.x (2006).

76. Lang, T. *et al.* Role of actin cortex in the subplasmalemmal transport of secretory granules in pc-12 cells. *Biophys. journal* **78**, 2863–2877, DOI: 10.1016/S0006-3495(00)76828-7 (2000).

77. Noda, Y., Horikawa, S., Katayama, Y. & Sasaki, S. Identification of a multiprotein motor complex binding to water channel aquaporin-2. *Biochem. Biophys. Res. Commun.* **330**, 1041–1047, DOI: 10.1016/j.bbrc.2005.03.079 (2005).

78. Pierobon, P. *et al.* Velocity, processivity, and individual steps of single myosin v molecules in live cells. *Biophys. J.* **96**, 4268–4275, DOI: 10.1016/j.bpj.2009.02.045 (2009).

79. Chou, C.-L. *et al.* Non-muscle myosin II and myosin light chain kinase are downstream targets for vasopressin signaling in the renal collecting duct. *J. Biol. Chem.* **279**, 49026–49035, DOI: 10.1074/jbc.m408565200 (2004).

80. Noda, Y. *et al.* Reciprocal interaction with g-actin and tropomyosin is essential for aquaporin-2 trafficking. *The J. cell biology* **182**, 587–601, DOI: 10.1083/jcb.200709177 (2008).

81. Jahn, R. & Scheller, R. H. SNAREs — engines for membrane fusion. *Nat. Rev. Mol. Cell Biol.* **7**, 631–643, DOI: 10.1038/nrm2002 (2006).

82. Franki, N., Macaluso, F., Gao, Y. & Hays, R. M. Vesicle fusion proteins in rat inner medullary collecting duct and amphibian bladder. *Am. J. Physiol. Physiol.* **268**, C792–C797, DOI: 10.1152/ajpcell.1995.268.3.c792 (1995).

83. Nielsen, S. *et al.* Expression of VAMP-2-like protein in kidney collecting duct intracellular vesicles. colocalization with aquaporin-2 water channels. *J. Clin. Investig.* **96**, 1834–1844, DOI: 10.1172/jci118229 (1995).

84. Low, S. H. *et al.* Differential localization of syntaxin isoforms in polarized madin-darby canine kidney cells. *Mol. Biol. Cell* **7**, 2007–2018, DOI: 10.1091/mbc.7.12.2007 (1996).

85. Mistry, A. C. *et al.* Syntaxin specificity of aquaporins in the inner medullary collecting duct. *Am. J. Physiol. Physiol.* **297**, F292–F300, DOI: 10.1152/ajprenal.00196.2009 (2009).

86. Donovan, K. W. & Bretscher, A. Tracking individual secretory vesicles during exocytosis reveals an ordered and regulated process. *J. Cell Biol.* **210**, 181–189, DOI: 10.1083/jcb.201501118 (2015).

87. Marples, D. *et al.* Dynein and dynactin colocalize with AQP2 water channels in intracellular vesicles from kidney collecting duct. *Am. J. Physiol. Physiol.* **274**, F384–F394, DOI: 10.1152/ajprenal.1998.274.2.f384 (1998).

88. Horgan, C. P., Hanscom, S. R., Jolly, R. S., Futter, C. E. & McCaffrey, M. W. Rab11-FIP3 links the rab11 GTPase and cytoplasmic dynein to mediate transport to the endosomal-recycling compartment. *J. Cell Sci.* **123**, 181–191, DOI: 10.1242/jcs.052670 (2009).

89. Toba, S., Watanabe, T. M., Yamaguchi-Okimoto, L., Toyoshima, Y. Y. & Higuchi, H. Overlapping hand-over-hand mechanism of single molecular motility of cytoplasmic dynein. *Proc. Natl. Acad. Sci.* **103**, 5741–5745, DOI: 10.1073/pnas.0508511103 (2006).

90. Lakadamyali, M., Rust, M. J. & Zhuang, X. Ligands for clathrin-mediated endocytosis are differentially sorted into distinct populations of early endosomes. *Cell* **124**, 997–1009, DOI: 10.1016/j.cell.2005.12.038 (2006).

91. Gooch, J. L. Loss of calcineurin a results in altered trafficking of AQP2 and in nephrogenic diabetes insipidus. *J. Cell Sci.* **119**, 2468–2476, DOI: 10.1242/jcs.02971 (2006).

92. Mirabet, V. *et al.* The self-organization of plant microtubules inside the cell volume yields their cortical localization, stable alignment, and sensitivity to external cues. *PLoS computational biology* **14**, e1006011, DOI: 10.1371/journal.pcbi.1006011 (2018).

93. Noda, Y. & Sasaki, S. The role of actin remodeling in the trafficking of intracellular vesicles, transporters, and channels: focusing on aquaporin-2. *Pflügers Arch. - Eur. J. Physiol.* **456**, 737–745, DOI: 10.1007/s00424-007-0404-2 (2007).

94. Bach, C. T., Murray, R. Z., Owen, D., Gaus, K. & O'Neill, G. M. Tropomyosin tm5nm1 spatially restricts src kinase activity through perturbation of rab11 vesicle trafficking. *Mol. Cell. Biol.* **34**, 4436–4446, DOI: 10.1128/mcb.00796-14 (2014).

95. Rizzoli, S. O. Synaptic vesicle recycling: steps and principles. *The EMBO J.* **33**, 788–822, DOI: 10.1002/embj.201386357 (2014).

96. Klann, M. & Koeppl, H. Spatial simulations in systems biology: from molecules to cells. *Int. journal molecular sciences* **13**, 7798–7827, DOI: 10.3390/ijms13067798 (2012).

97. Vale, R. D. The molecular motor toolbox for intracellular transport. *Cell* **112**, 467–480, DOI: 10.1016/s0092-8674(03)00111-9 (2003).

98. Ernst, J. A. & Brunger, A. T. High resolution structure, stability, and synaptotagmin binding of a truncated neuronal SNARE complex. *J. Biol. Chem.* **278**, 8630–8636, DOI: 10.1074/jbc.m211889200 (2002).

99. Karatekin, E. *et al.* A fast, single-vesicle fusion assay mimics physiological SNARE requirements. *Proc. Natl. Acad. Sci.* **107**, 3517–3521, DOI: 10.1073/pnas.0914723107 (2010).

100. Burgess, S. A., Walker, M. L., Sakakibara, H., Knight, P. J. & Oiwa, K. Dynein structure and power stroke. *Nature* **421**, 715–718, DOI: 10.1038/nature01377 (2003).

101. Nielsen, S. & Agre, P. The aquaporin family of water channels in kidney. *Kidney international* **48**, 1057–1068, DOI: 10.1038/ki.1995.389 (1995).

102. Strange, K. & Spring, K. R. Cell membrane water permeability of rabbit cortical collecting duct. *The J. membrane biology* **96**, 27–43, DOI: 10.1007/bf01869332 (1987).

103. Quinn, P. Density of newly synthesized plasma membrane proteins in intracellular membranes II. biochemical studies. *The J. Cell Biol.* **98**, 2142–2147, DOI: 10.1083/jcb.98.6.2142 (1984).
